# Optimal metacognitive decision strategies in signal detection theory

**DOI:** 10.3758/s13423-024-02510-7

**Published:** 2024-11-18

**Authors:** Brian Maniscalco, Lucie Charles, Megan A. K. Peters

**Affiliations:** 1 Department of Cognitive Sciences, University of California Irvine, Irvine, CA 92697 USA; 2 Institute of Cognitive Neuroscience, University College London, Alexandra House, 17 Queen Square, London, WC1N 3AZ UK; 3 School of Biological and Behavioural Sciences, Queen Mary, University of London, London, United Kingdom

**Keywords:** Metacognition, Decision-making, Signal detection theory, Computational modelling

## Abstract

Signal detection theory (SDT) has long provided the field of psychology with a simple but powerful model of how observers make decisions under uncertainty. SDT can distinguish sensitivity from response bias and characterize optimal decision strategies. Whereas classical SDT pertains to “type 1” judgments about the world, recent work has extended SDT to quantify sensitivity for metacognitive or “type 2” judgments about one’s own type 1 processing, e.g. confidence ratings. Here we further advance the application of SDT to the study of metacognition by providing a formal account of normative metacognitive decision strategies – i.e., type 2 (confidence) criterion setting – for ideal observers. Optimality is always defined relative to a given objective. We use SDT to derive formulae for optimal type 2 criteria under four distinct objectives: maximizing type 2 accuracy, maximizing type 2 reward, calibrating confidence to accuracy, and maximizing the difference between type 2 hit rate and false alarm rate. Where applicable, we consider these optimization contexts alongside their type 1 counterparts (e.g. maximizing type 1 accuracy) to deepen understanding. We examine the different strategies implied by these formulae and further consider how optimal type 2 criterion setting differs when metacognitive sensitivity deviates from SDT expectation. The theoretical framework provided here can be used to better understand the metacognitive decision strategies of real observers. Possible applications include characterizing observers’ spontaneously chosen metacognitive decision strategies, assessing their ability to fine-tune metacognitive decision strategies to optimize a given outcome when instructed, determining over- or under-confidence relative to an optimal standard, and more. This framework opens new avenues for enriching our understanding of metacognition.

**Supplementary information:**

The online version contains supplementary material available at 10.3758/s13423-024-02510-7.

## Introduction

Signal detection theory (SDT) has long provided the field of psychology with a simple but powerful model of how observers make decisions under uncertainty (Green & Swets, 1966; Macmillan & Creelman, 2004). Central to SDT is the distinction between sensitivity and criterion setting. Sensitivity corresponds to an observer’s overall ability to distinguish different states of the world (e.g. the visual sensitivity of detecting the presence or absence of a visual event, the memory sensitivity of discerning whether a stimulus has been encountered before or not, etc.). Criterion setting corresponds to the decision-making strategy the observer uses to convert graded and potentially ambiguous internal evidence into a definite classification (e.g. deciding to report that a visual event did indeed occur, in spite of uncertainty about its occurrence). Whereas sensitivity is constrained by various external and internal factors (e.g. in a visual task, the size, duration, and contrast of the stimulus, the observer’s visual acuity and available attentional and motivational resources, etc.), criterion setting is largely under the observer’s control and can be altered depending on task demands. For instance, the observer may choose to be more liberal or conservative in reporting stimulus presence depending on his or her internal bias, or what is most appropriate to the current context.

The SDT framework can be used not only to *describe* an observer’s response strategy, but also to determine a *normative* strategy for how the observer *should* set their decision criterion depending on their goals and priorities. If the observer is trying to optimize a particular outcome measure, such as reward or accuracy, it is then possible to compute how they should set their criterion, given their particular level of sensitivity and the decision-making context. For instance, suppose the observer is performing a visual task in which they must determine on every trial whether a stimulus was presented on the left or right side of fixation. If the stimulus is considerably more likely to appear on the left and the observer wishes to maximize their probability of making correct decisions, then it is optimal for them to adopt a criterion biased towards left responses. Indeed, a criterion that requires relatively stringent standards for reporting “right” – essentially giving “left” responses the benefit of the doubt and only reporting “right” when evidence for right is particularly strong – will lead to more correct responses. The exact level of conservativeness in reporting “right” needed to optimize accuracy can be computed from the odds of stimulus location and the observer’s sensitivity, as discussed below. The *actual* and *optimal* criteria can then be compared to one another in order to assess to what extent the observer’s decision-making strategy deviates from the optimal strategy for maximizing accuracy.

Importantly, optimality can only ever be assessed with respect to a definite outcome measure or loss function, and the optimal strategy is therefore likely to differ for different outcome measures. We should thus never speak of “optimality” in absolute terms, but only speak of “optimality *for* X”. For instance, in the example above, the observer chooses a criterion setting strategy that optimizes accuracy. Specifically, because stimuli frequently occur on the left side of fixation, the observer optimizes accuracy by choosing to be liberal in responding “left.” However, suppose we introduce rewards for correct responses such that the observer is now very heavily rewarded for making correct “right” responses, but only lightly rewarded for correct “left” responses. The observer may now be less interested in optimizing for accuracy than in optimizing for expected reward. Indeed, if the asymmetry in rewards is strong enough, then the optimal strategy for maximizing reward may be to be liberal in producing “right” responses, even despite the fact that stimuli more frequently occur on the left. In other words, what is optimal for one outcome measure is not necessarily optimal for another.

In addition to discriminating states of the world, the observer may also discriminate their own internal states. For instance, the observer may wish to determine not only whether the stimulus was on the left or right (a “type 1” judgment about the world), but also whether the subjective experience of the stimulus was clear or vivid, or whether the left/right decision can be endorsed with high confidence (both being “type 2” or “metacognitive” judgments about the nature of the observer’s internal perceptual processing)^1^. The SDT framework can be extended to type 2 decision-making (Galvin et al., 2003; Maniscalco & Lau, 2012, 2014). In type 2 SDT, sensitivity corresponds to how well an observer’s type 2 reports (e.g. confidence or subjective clarity) discriminate between correct and incorrect type 1 decisions about the world, and criterion setting refers to the observer’s strategy for producing reports of high confidence (or clarity, etc.). Type 2 sensitivity is constrained by various factors (including type 1 sensitivity) and extensive literature has explored how to measure it in a meaningful way (Barrett et al., 2013; Fleming & Daw, 2017; Fleming & Lau, 2014). Importantly, although type 2 criterion setting is a central aspect of modelling confidence judgments using signal detection theory, it remains less studied and poorly understood (Sherman et al., 2018). In particular, it remains unclear how the demands of the decision-making context affect the confidence criterion-setting strategy for both ideal^2^ and actual observers (Fleming & Dolan, 2010; Lebreton et al., 2018; Locke et al., 2020), and to what extent actual observers’ strategies resemble ideal strategies for optimizing various outcome measures of metacognitive performance.

As with type 1 decisions, it is possible to mathematically characterize the *normative* type 2 criterion-setting strategy an observer should adopt in order to optimize some outcome measure that depends on the confidence report. These normative strategies can then be compared to an observer’s actual type 2 criterion setting behavior to characterize to what extent the observer’s strategy deviates from optimality (Fleming & Dolan, 2010; Lebreton et al., 2018; Locke et al., 2020). Crucially however, normative type 2 criterion setting has not yet been formally characterized in the literature. Thus, in the present paper we explore type 2 criterion setting strategies for optimizing different type 2 outcome measures. We consider four main strategies for determining the position of confidence criteria: (1) maximize the accuracy of confidence reports in discriminating correct from incorrect type 1 responses; (2) maximize the reward obtained from confidence report according to the experimental reward schedule; (3) calibrate confidence so that high confidence reports are associated with a minimal level of choice accuracy (e.g. only report “high confidence” when the choice is at least 80% likely to be correct); or (4) maximize the difference between the proportion of high confidence reports for correct vs incorrect type 1 responses. For each strategy, we provide equations defining the optimal type 2 criteria (with full derivations provided in Supplementary Material S2) and give visual intuitions for how these optimal criteria change with various factors such as task performance, stimulus priors, reward contingencies, etc. Where applicable, we present these equations and their derivations side-by-side with their type 1 analogues, as it is instructive to compare and contrast optimal criterion setting in the type 1 and type 2 cases. Finally, we also use simulations to explore how optimal type 2 criterion setting changes under conditions of suboptimal metacognitive sensitivity, i.e. when an observer’s confidence fails to optimally track type 1 accuracy on a trial-by-trial basis according to SDT. This work can inform future research that seeks to characterize type 2 criterion setting relative to optimality for various kinds of type 2 outcome measures.

## Optimizing criterion setting for different tasks and goals

**Fig. 1:**
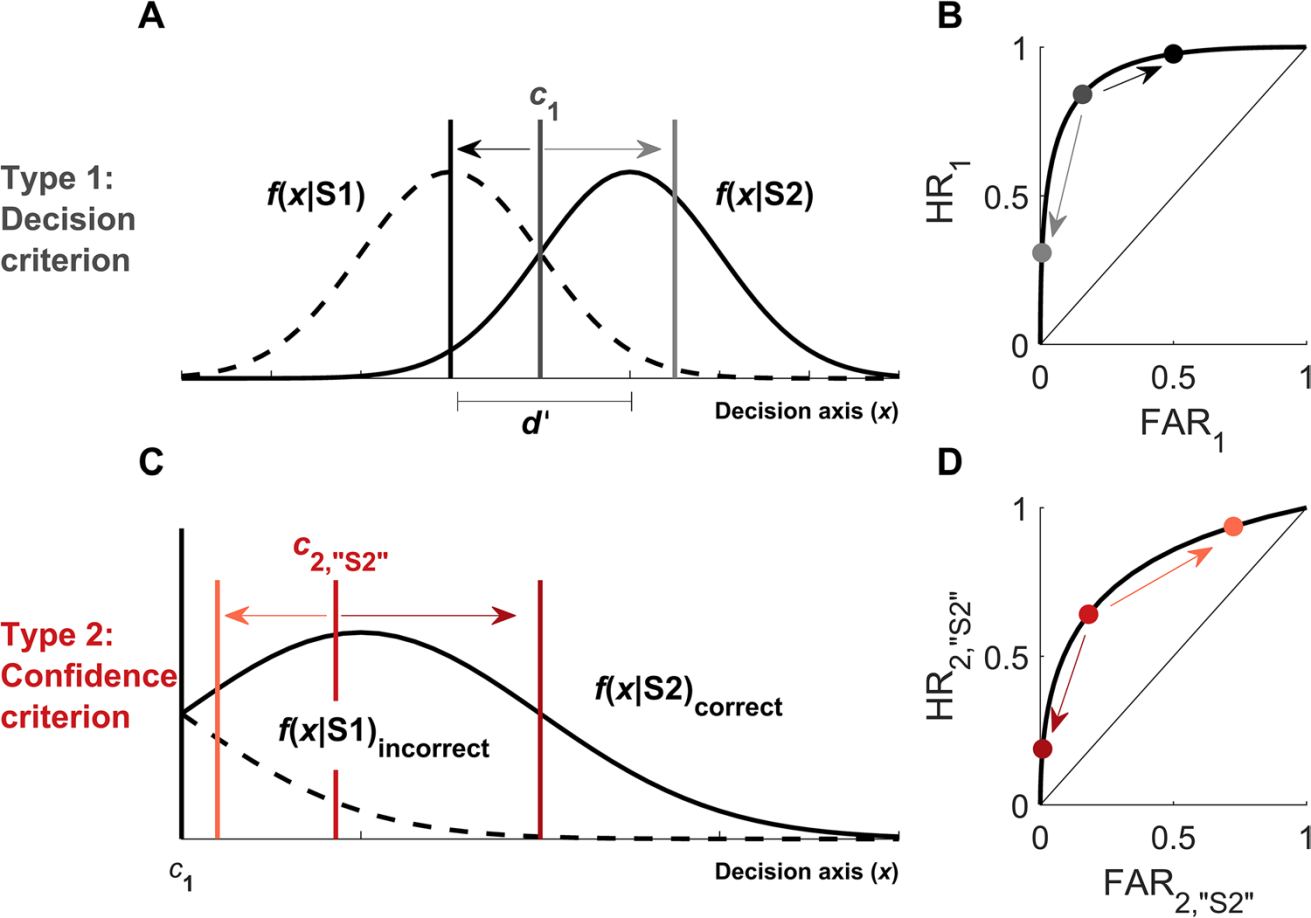
Type 1 and type 2 criterion in Signal Detection Theory. **(A)** The standard type 1 signal detection model. The observer discriminates between two stimuli S1 and S2. Evidence generated by each stimulus is distributed normally on the decision axis (denoted as “*x*”) and the distance between the two distributions, expressed in standard deviation units, reflects the sensitivity of the observer to discriminate between the two stimuli (*d’*). The observer’s type 1 criterion *c*_1_ corresponds to the signal *x* above which the observer will report “S2” rather than “S1”. When S1 and S2 stimuli are equally probable, the criterion that maximizes decision accuracy is placed midway between the two distributions, illustrated here by a dark gray vertical line labeled *c*_1_. If the observer’s decision criterion is shifted to the left, the observer will respond “S2” more often (black vertical line) and if the observer’s decision criterion is shifted to the right, the observer will respond “S1” more often (light gray vertical line). **(B)** Type 1 Receiver Operating Characteristic (ROC) curve corresponding to the hit rates (*p*(“S2” response | S2 stimulus)) and false alarm rates (*p*(“S2” response | S1 stimulus)) for an observer with a given level of sensitivity (*d’*) but a varying decision criterion (*c*_1_). Decision criteria highlighted in **(A)** are also highlighted on the ROC curve to illustrate their effect on hit and false alarm rates. **(C)** The type 2 signal detection model for “S2” responses. Since “S2” responses occur when *x* > *c*_1_, here we consider only the portion of the graph in **(A)** to the right of *c*_1_. The observer sets a type 2 criterion *c*_2,“S2”_ ≥ *c*_1_ (red vertical line) that defines the signal above which the observer will respond “high confidence” rather than “low confidence” for “S2” responses. If *c*_2,“S2”_ is shifted towards *c*_1_, the observer will respond “high confidence” more often (light red vertical line) and if *c*_2,“S2”_ is shifted away from *c*_1_, the observer will respond “high confidence” less often (dark red vertical line). The type 2 model for “S1” responses (not shown) follows similar principles: here the observer sets a type 2 criterion *c*_2,“S1”_ ≤ *c*_1_ such that evidence values smaller than *c*_2,“S1”_ yield “high confidence” responses, and “low confidence” otherwise, and *c*_2,“S1”_ becomes more liberal or conservative as it is shifted closer to or farther from *c*_1_, respectively. **(D)** Type 2 ROC curve for “S2” responses, corresponding to the type 2 hit rates (*p*(“high confidence” response | correct “S2” response)) and type 2 false alarm rates (p(“high confidence” response | incorrect “S2” response)), for an observer with a given level of sensitivity (*d’*) and type 1 decision criterion (*c*_1_, black vertical line) but varying confidence criterion (*c*_2,“S2”_). Confidence criteria highlighted in **(C)** are also highlighted on the type 2 ROC curve to illustrate their effect on type 2 hit and false alarm rates. Note that type 2 hit and false alarm rates can be derived from the graph in **(C)** after normalizing the distributions so that the area under each curve sums to 1. Similar principles hold for the construction of the type 2 ROC curve for “S1” responses

The following discussion assumes the reader is familiar with classical signal detection theory and its extension to response-specific type 2 decision making. To briefly review, type 1 SDT models how an observer performs the task of discriminating whether a stimulus (e.g. a grating) belongs to class S1 (e.g. left tilt) or S2 (e.g. right tilt). The model assumes that on each trial, the observer perceives a certain magnitude of evidence associated with the stimulus (e.g. evidence for left vs right tilt), with lower and higher values being more associated with S1 and S2, respectively. The observer classifies the stimulus as “S2” if the evidence on this trial exceeds a criterion value *c*_1_, and “S1” otherwise^3^. These evidence values are stochastic, such that over repeated presentations, S1 and S2 generate overlapping normal distributions of evidence (Figure 1A). (For simplicity, in this paper we make a standard assumption that the standard deviations of the S1 and S2 distributions are equal^4^.) This model captures empirical patterns in type 1 ROC curves, which describe how type 1 hits and false alarms trade off with changes in the observer’s criterion (Figure 1B). The type 2 SDT model posits that confidence ratings for “S1” and “S2” responses are similarly produced using additional type 2 decision criteria on either side of the type 1 criterion (Figure 1C), which can capture empirical patterns in type 2 ROC curves (Figure 1D). For a more in-depth review of type 1 and type 2 SDT, please see Supplementary Material S1.

In deciding how to set type 1 and type 2 criteria, an observer ought to consider the goals they want to achieve. What is the quantity they want to maximize or minimize? For example, an observer might want to maximize accuracy or reward in the type 1 task. Likewise, in the type 2 task the observer might want to maximize the correspondence between confidence and accuracy, or reward contingent on confidence ratings (Fleming & Dolan, 2010; Lebreton et al., 2018; Locke et al., 2020), and so on.

In this section we investigate optimal type 2 criterion setting for four outcome measures that an observer might wish to optimize: (1) type 2 accuracy, (2) type 2 reward, (3) calibration of confidence to expected accuracy, and (4) the difference between type 2 hit rate and false alarm rate. To deepen understanding, we consider scenarios (1), (2), and (4) in the context of their natural type 1 counterparts (i.e. setting the type 1 criterion to optimize type 1 accuracy, type 1 reward, and the difference between type 1 hit rate and false alarm rate).

How should the observer set their type 2 criteria so as to achieve optimal outcomes? The general strategy for answering this question involves (1) writing down an equation for the outcome measure to be optimized, (2) using SDT to re-express the equation in terms of a response-specific type 2 criterion, and (3) using calculus to solve for the value of the type 2 criterion that maximizes the outcome measure. (The strategy we employ for calibration is somewhat different, as discussed below.) The derived equations reveal how optimal type 2 criterion setting depends on factors such as prior probability of stimulus presentation (*p*(S2)), type 1 sensitivity (*d’*), type 1 criterion (*c*_1_), and others.

For all the optimization contexts considered here, there is only one type 2 criterion value that optimizes the outcome measure for a given type 1 response. Setting a single type 2 criterion partitions the decision axis into two regions corresponding to “low” and “high” confidence. Thus, the question of finding *the* single type 2 criterion that is optimal very naturally and straightforwardly lends itself to mathematical analysis using a binary confidence scale. Many experiments use confidence rating scales with more than two options, and for a scale with *N* ratings, the observer must set *N*-1 type 2 criteria for each response type. Nonetheless, if the observer is attempting to find *the* type 2 criterion that optimizes one of these outcome measures, this attempt can only pertain to one of those *N*-1 type 2 criteria. Thus, even for larger rating scales, the problem of optimizing the measures considered here reduces to consideration of the single optimal type 2 criterion that achieves the best binarization of confidence reports, and so our approach is applicable to data using larger rating scales. See Section "Optimal type 2 criterion setting for confidence rating scales with more than two levels" for further discussion.

**Fig. 2:**
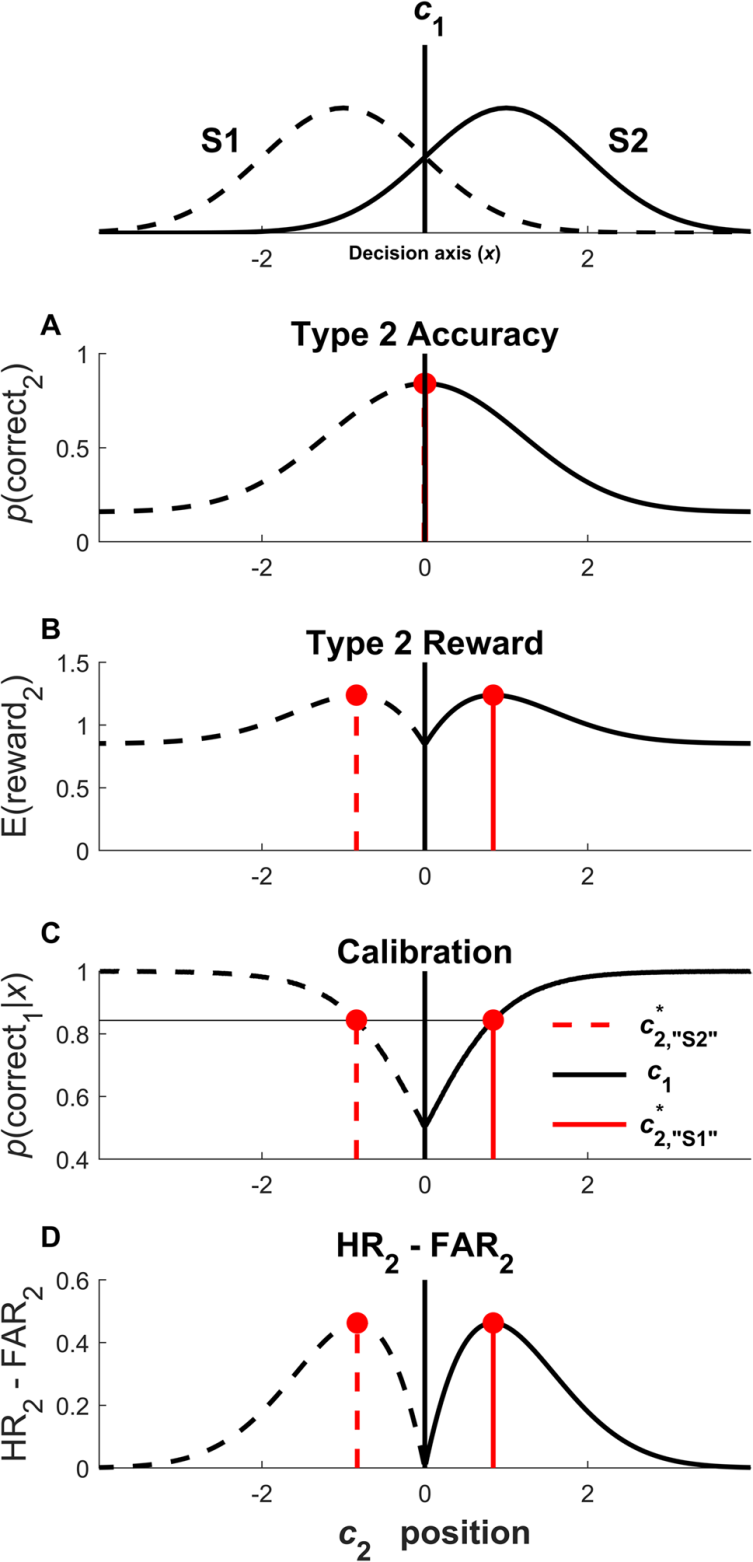
Example of four distinct outcome measures as a function of the positions of the confidence criteria. Top panel shows signal distributions for an unbiased observer (*c*_1_ = 0) with *d’* = 2 and equal probability of S1 and S2 used for the simulations. Black vertical lines depict type 1 criterion *c*_1_; dashed red lines depict optimal type 2 criterion ${c}_{2, {``}\mathrm{S}1\text{''}}^{*}$ (the superscript capital letter is omitted for generality) for “S1” responses and solid red lines depict optimal type 2 criterion ${c}_{2,``\mathrm{S}2\text{''}}^{*}$ for “S2” responses. **(A)** Type 2 accuracy as a function of *c*_2_ position on the decision axis. For this unbiased observer, type 2 accuracy decreases when placing the type 2 criterion further away from the type 1 criterion, and the optimal type 2 criterion is therefore identical to the type 1 criterion. **(B)** Expected type 2 reward as a function of *c*_2_ position for a reward contingency table where reporting low confidence for error trials is strongly rewarded (R_CR2_ = 5.37, R_hit2_ = 1, R_miss2_ = R_FA2_ = 0). **(C)** Expected type 1 accuracy as a function of evidence sample *x*. If the observer wishes to report high confidence only for trials where expected type 1 accuracy exceeds some threshold *p*(correct_1_)_T_, then they must place type 2 criteria at the locations where the function *p*(correct_1_|*x*) intersects *p*(correct_1_)_T_. In this example the threshold *p*(correct_1_)_T_ is set equal to 0.843, which is equal to the observer’s overall type 1 accuracy when *d’* = 2 and *c*_1_ = 0. **(D)** Difference in type 2 hit and false-alarm rates as a function of *c*_2_ position. These example plots are constructed so that the optimal type 2 criteria are the same in panels **B**-**D**, illustrating that in special cases, optimal type 2 criterion setting can be equivalent across different contexts. Specifically, when priors are equal (*p*(S1) = *p*(S2)) and the observer is unbiased (*c*_1_ = 0), maximizing HR_2_ - FAR_2_ is equivalent to calibrating confidence to one’s overall level of type 1 accuracy, i.e. setting *p*(correct_1_)_T_ = *p*(correct_1_); in this example, both equal 0.843. In turn, calibration is equivalent to maximizing type 2 reward when the odds ratio of a correct response at the threshold level of accuracy for calibration (*O*_T_) is equal to the relative reward quotient for maximizing reward (*Q*_2_); in this example, both equal 5.37. See Sections "Calibrating confidence to expected accuracy" and "Optimal type 2 criteria for maximizing HR_2_- FAR_2_" for further discussion of these equivalences

In Figure 2, we show an initial example of optimal type 2 criterion setting under the four optimization contexts considered in this paper. These plots show how the outcome measure to be optimized varies as a function of placement of the type 2 criteria for “S1” and “S2” responses. For type 2 accuracy (Figure 2A), type 2 reward (Figure 2B), and HR_2_ - FAR_2_ (Figure 2D), the outcome measures for each response type are unimodal functions of the type 2 criteria, and the optimal type 2 criteria are the ones occurring at the maximal values of these outcome functions (denoted in red). For calibration (Figure 2C), the optimal type 2 criteria correspond to locations on the decision axis where expected task accuracy given an evidence sample *x* is equal to a target level of accuracy. These plots illustrate the basic idea behind the modeling and derivation of optimal type 2 criteria in SDT as explored below. We have also specially constructed these example plots so that the optimal type 2 criteria for type 2 reward (Figure 2B), calibration (Figure 2C), and HR_2_ - FAR_2_ (Figure 2D) are identical, which illustrates the principle that in certain special cases, optimal type 2 criterion setting can be equivalent across different contexts. We elaborate on this idea throughout the paper.

Notation used in all derivations and presentation of results is shown in Table 1, and expanded derivations of optimal criteria for all outcome measures discussed below are provided in Supplementary Material S2.

**Table 1:** Notation used in derivations (see Supplementary Material S2) for finding the optimal type 1 and type 2 criteria under the four optimization contexts: accuracy, reward, calibration, and hit rate minus false alarm rate. The use of * indicates that the criterion in question is the optimal criterion under the context denoted by superscript capital letters, while the subscript of 1 or 2 indicates whether this criterion is for type 1 or type 2 decisions, respectively

optimization context	type 1 criterion $\varvec{c}_{1}$	type 2 criterion $\varvec{c}_{2}$	
		for “S1” responses	for “S2” responses
accuracy (A)	${c}_{1}^{\mathrm{A}^*}$	${c}_{2,``\mathrm{S}1"}^{\mathrm{A}{^*}}$	${c}_{2,``\mathrm{S}2"}^{\mathrm{A}^*}$
reward (R)	${c}_{1}^{\mathrm{R}^*}$	${c}_{2,``\mathrm{S}1"}^{{\mathrm{R}}^*}$	${c}_{2,``\mathrm{S}2"}^{\mathrm{R}^*}$
calibration (C)	$\textrm{N/A}$	${c}_{2,``\mathrm{S}1"}^{{\mathrm{C}}^*}$	${c}_{2,``\mathrm{S}2"}^{\mathrm{C}^*}$
hit rate - false alarm rate (HF)	${c}_{1}^{\mathrm{HF}^*}$	${c}_{2,``\mathrm{S}1"}^{\mathrm{HF}^*}$	${c}_{2,``\mathrm{S}2"}^{\mathrm{HF}^*}$

### Optimizing accuracy

#### Optimal type 1 criterion for maximizing type 1 accuracy

Suppose an observer with sensitivity *d’* wishes to place the type 1 criterion *c*_1_ so as to maximize proportion of correct responses in discriminating two stimulus classes, S1 and S2, where *p*(S1) and *p*(S2) denote the prior probabilities of S1 and S2 being presented on a given trial. As discussed in the SDT primer (Supplementary Material S1), when *p*(S1) = *p*(S2), the optimal location for the type 1 criterion *c*_1_ is at *x* = 0, or equivalently at *β*_1_ = 1. In the more general case where *p*(S1) and *p*(S2) can differ, the placement of the criterion needs to account for these prior probabilities. The measure to be optimized, proportion of correct type 1 responses, is given by

1 $$p({\mathrm{correct}}_{1})=p(\mathrm{S2}) \: {\mathrm{HR}}_{1} + p(\mathrm{S1}) \: (1-{\mathrm{FAR}}_{1})$$

where HR_1_ = type 1 hit rate = *p*(response = “S2” | stimulus = S2) and FAR_1_ = type 1 false alarm rate = *p*(response = “S2” | stimulus = S1). The value of *c*_1_ that maximizes type 1 accuracy is given by

2 $${c}_{1}^{\mathrm{A}^*}=\frac{ \ln \frac{p(\mathrm{S1})}{p(\mathrm{S2})} }{d^{\prime}}$$

An alternative formulation for the decision criterion *c*_1_ is as the *likelihood ratio* of the stimulus distributions $\frac{f(x|\mathrm{S2})}{f(x|\mathrm{S1})}$ occurring at the location of the criterion (i.e. the likelihood ratio at *x* = *c*_1_). This likelihood ratio *β*_1_ is given by ${\beta }_{1}=\frac{f({c}_{1}|\mathrm{S2})}{f({c}_{1}|\mathrm{S1})}={e}^{{c}_{1}{d}^{\prime}}$. Thus, for an unbiased observer with *c*_1_ = 0, the criterion can be expressed instead as *β*_1_ = 1, i.e. for an unbiased observer the criterion is placed at the point on the decision axis where *x* is equally likely to have been generated by S1 and S2. Similarly, *β*_1_ < 1 for a liberal criterion and *β*_1_ > 1 for a conservative criterion. (For brevity, we do not repeat all the derivations in the remainder of this manuscript with the likelihood ratio notation, but the interested reader can refer to Supplementary Material S1 and Supplementary Material S2, Section 5 for further discussion.) Re-expressing ${c}_{1}^{\mathrm{A}^*}$ as the likelihood ratio ${\beta }_{1}^{\mathrm{A}^*}$ gives

3 $${\beta }_{1}^{\mathrm{A}^*}=\frac{f({c}_{1}^{\mathrm{A}^*}|\mathrm{S2})}{f({c}_{1}^{\mathrm{A}^*}|\mathrm{S1})}={e}^{{c}_{1}^{\mathrm{A}^*}d^{\prime}}=\frac{p(\mathrm{S1})}{p(\mathrm{S2})}$$

**Fig. 3:**
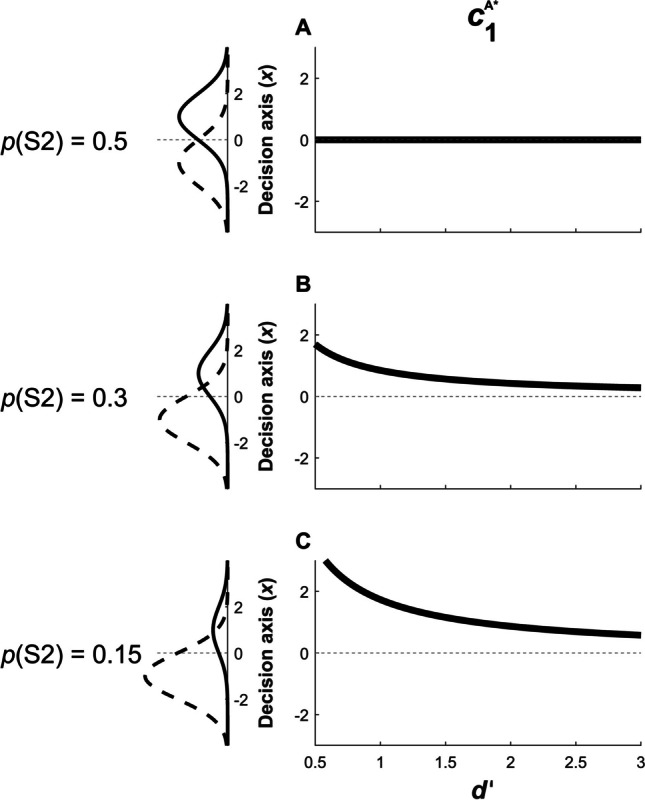
Optimal position of the type 1 criterion ${c}_{1}^{\mathrm{A}^*}$ which maximizes type 1 accuracy as a function of *d’* and *p*(S2). Each row represents different probabilities of stimulus presentation: **(A)** *p*(S2) = 0.5; **(B)** *p*(S2) = 0.3; **(C)** *p*(S2) = 0.15. Here instead of the traditional probability density functions conditional upon stimulus presentation, *f*(*x*|S1) and *f*(*x*|S2), we plot joint distribution functions *f*(*x*, S1) and *f*(*x*, S2) in the left column for a fixed *d’* value of 2 to illustrate how stimulus priors affect optimal criterion setting. In this representation, the optimal type 1 criterion ${c}_{1}^{\mathrm{A}^*}$ corresponds to where the functions intersect; thus e.g. as *f*(*x*, S2) loses probability mass with the reduction of *p*(S2) (solid black lines in left column), the location where *f*(*x*, S1) and *f*(*x*, S2) intersect becomes increasingly positive, meaning ${c}_{1}^{\mathrm{A}^*}$ becomes increasingly conservative. Plots in the right column show how the value of this increasingly conservative criterion is modulated by different *d’* values. Y-axes in the right panels represent the internal decision axis, as displayed in the left panels. Similarly, as *p*(S2) increases above 0.5, ${c}_{1}^{\mathrm{A}^*}$ becomes increasingly liberal (not shown)

From Eq. 3 it is clear why, when presentation of S1 and S2 is equally probable, the observer maximizes type 1 accuracy by setting ${c}_{1}={c}_{1}^{\mathrm{A}^*}=0$ (equivalently ${\beta }_{1}={\beta }_{1}^{\mathrm{A}^*}=1$), i.e. at the location at which the distributions intersect and evidence is equally likely to have been generated by S1 or S2 (Figure 3A). In this case, decisions depend only on the relative likelihoods of the evidence *x* under S1 and S2 – the observer responds “S2” whenever *x* has higher probability density for S2 than for S1 (*c*_1_ > 0 and *β*_1_ > 1), and “S1” otherwise.

When priors are imbalanced (Figure 3B-C), the observer must compensate by incorporating an equal and opposite imbalance in how they evaluate evidence. This is accomplished by setting the criterion at the location of the decision axis where the likelihood ratio of S2 to S1 (i.e. *β*_1_) matches the prior probability ratio of S1 to S2 (Eq. 3). For instance, if S2 is half as likely to be presented as S1, then the observer should have a conservative response bias, only responding “S2” if the evidence for S2 is at least twice as strong as the evidence for S1.

This decision-making policy is consistent with Bayesian reasoning. A Bayesian observer should respond “S2” whenever S2 has the greater posterior probability, i.e. whenever $\frac{p(\mathrm{S2}|x) }{p(\mathrm{S1}|x)}>1$, and respond “S1” otherwise. Using Bayes’ rule, this ratio of posterior probabilities can be expressed as the Bayes factor, i.e. $\frac{p(\mathrm{S2}|x) }{p(\mathrm{S1}|x)}=\frac{p(x|\mathrm{S2})p(\mathrm{S2}) }{p(x|\mathrm{S1})p(\mathrm{S1})}$. Substituting the Bayes factor into the inequality $\frac{p(\mathrm{S2}|x) }{p(\mathrm{S1}|x)}>1$ and rearranging yields a policy of responding “S2” whenever $\frac{p(x|\mathrm{S2}) }{p(x|\mathrm{S1})}>\frac{p(\mathrm{S1}) }{p(\mathrm{S2})}$, which is equivalent to the decision-making policy implicit in Eq. 3. Thus, the criterion setting policy for maximizing accuracy (Eqs. 2-3) can be understood intuitively as a decision policy to respond “S2” whenever S2 is the most probable stimulus given the evidence *x*, i.e. whenever $\frac{p(\mathrm{S2}|x) }{p(\mathrm{S1}|x)}>1$.

Derivation of the criterion that optimizes type 1 accuracy (${c}_{1}^{\mathrm{A}^*}$, Eq. 2) is provided in Supplementary Material S2, Section 1.1.

#### Optimal type 2 criteria for maximizing type 2 accuracy

Type 2 task performance can be understood by way of analogy to the type 1 case. Just as in the type 1 task the observer must classify S1 and S2 stimuli with type 1 responses “S1” and “S2,” so in the type 2 task the observer must classify correct and incorrect type 1 responses with type 2 responses “high confidence” and “low confidence.”

Extending the analogy, we can consider a type 2 response to be “correct” when it is congruent with accuracy and incorrect otherwise, as summarized in Table 2. For clarity and brevity, we write “correct_1_” to denote “type 1 correct” and “correct_2_” to denote “type 2 correct,” and similarly for incorrect trials.

**Table 2:** Types of responses that can be given in type 1 and type 2 tasks. Subscripts denote the type of task

type 1 task		
event	classification	
	response = “S1”	response = “S2”
stimulus = S1	correct_1_	incorrect_1_
stimulus = S2	incorrect_1_	correct_1_
type 2 task		
event	classification	
	confidence = low	confidence = high
accuracy = incorrect_1_	correct_2_	incorrect_2_
accuracy = correct_1_	incorrect_2_	correct_2_

Also by way of analogy to the type 1 case, the observer may wish to set type 2 criteria so as to maximize *p*(correct_2_) – i.e., the probability that confidence reports are congruent with accuracy – where

4 $$p({\mathrm{correct}}_{2})=p({\mathrm{correct}}_{1}) \: {\mathrm{HR}}_{2}+p({\mathrm{incorrect}}_{1}) \: (1-{\mathrm{FAR}}_{2})$$

where HR_2_ = type 2 hit rate = *p*(high conf | correct_1_) and FAR_2_ = type 2 false alarm rate = *p*(high conf | incorrect_1_) (compare to Eq. 1).

Notably, whereas in the type 1 case the event priors *p*(S1) and *p*(S2) depend on observer-independent states of the world, in the type 2 case the event priors *p*(correct_1_) and *p*(incorrect_1_) are determined by the observer’s type 1 task performance. As a consequence, unlike in the type 1 case where it is usually convenient for the experimenter to set *p*(S1) = *p*(S2), in the type 2 case the event priors are only equal in the special case where *p*(correct_1_) = *p*(incorrect_1_), i.e. when the observer’s type 1 task performance is at chance. As task performance increases, so does the disparity in the type 2 event priors *p*(correct_1_) and *p*(incorrect_1_).

It is mathematically convenient to characterize type 2 performance separately for type 1 “S1” and “S2” responses. For “S2” responses, the observer must set the type 2 criterion for “S2” responses, *c*_2,“S2”_, so as to maximize type 2 accuracy for “S2” responses, *p*(correct_2,“S2”_):

5 $$p\!\left({\mathrm{correct}}_{2,{``\mathrm{S}2"}}\right) = p\!\left({\mathrm{correct}}_{1,{``\mathrm{S}2"}}\right) {\mathrm{HR}}_{2,{``\mathrm{S}2"}} + p\!\left({\mathrm{incorrect}}_{1,{``\mathrm{S}2"}}\right) \left(1-{\mathrm{FAR}}_{2,{``\mathrm{S}2"}}\right)$$

where *p*(correct_1,”S2”_) = *p*(correct_1_ | resp = “S2”), *p*(incorrect_1,”S2”_) = *p*(incorrect_1_ | resp = “S2”), HR_2,”S2”_ = type 2 hit rate for “S2” responses = *p*(high conf | correct_1,”S2”_), and FAR_2,”S2”_ = type 2 false alarm rate for “S2” responses = *p*(high conf | incorrect_1,”S2”_). The value of *c*_2,“S2”_ that maximizes *p*(correct_2,“S2”_) is given by

6 $${c}_{2,{``\mathrm{S}2"}}^{\mathrm{A}^*}= \max \! \left(\frac{\ln \frac{p(\mathrm{S1})}{p(\mathrm{S2})} }{d^{\prime}},{c}_{1} \right)=\max\!\left({c}_{1}^{\mathrm{A}^*},{c}_{1} \right)$$

where *c*_1_ denotes the actual (and possibly suboptimal) type 1 criterion set by the observer. Similarly for “S1” responses, the observer must set *c*_2,“S1”_ so as to maximize *p*(correct_2,“S1”_):

7 $$p\!\left({\mathrm{correct}}_{2,{``\mathrm{S}1"}}\right)=p\!\left({\mathrm{correct}}_{1,{``\mathrm{S}1"}}\right) {\mathrm{HR}}_{2,{``\mathrm{S}1"}}+p\!\left({\mathrm{incorrect}}_{1,{``\mathrm{S}1"}}\right) \left(1-{\mathrm{FAR}}_{2,{``\mathrm{S}1"}}\right)$$

The value of *c*_2,“S1”_ that maximizes *p*(correct_2,“S1”_) is given by

8 $${c}_{2,{``\mathrm{S}1"}}^{\mathrm{A}^*}= \min\!\left(\frac{\ln \frac{p(\mathrm{S1})}{p(\mathrm{S2})} }{d^{\prime}},{c}_{1} \right)= \min\!\left({c}_{1}^{\mathrm{A}^*},{c}_{1} \right)$$

Derivations of the type 2 criteria that optimize type 2 accuracy for “S2” and “S1” responses (${c}_{2,\mathrm{``S2"}}^{\mathrm{A}^*}$ and ${c}_{2,\mathrm{``S1"}}^{\mathrm{A}^*}$ via Eqs. 6 and 8, respectively) are provided in Supplementary Material S2, Sections 1.2.1 and 1.2.2.

**Fig. 4:**
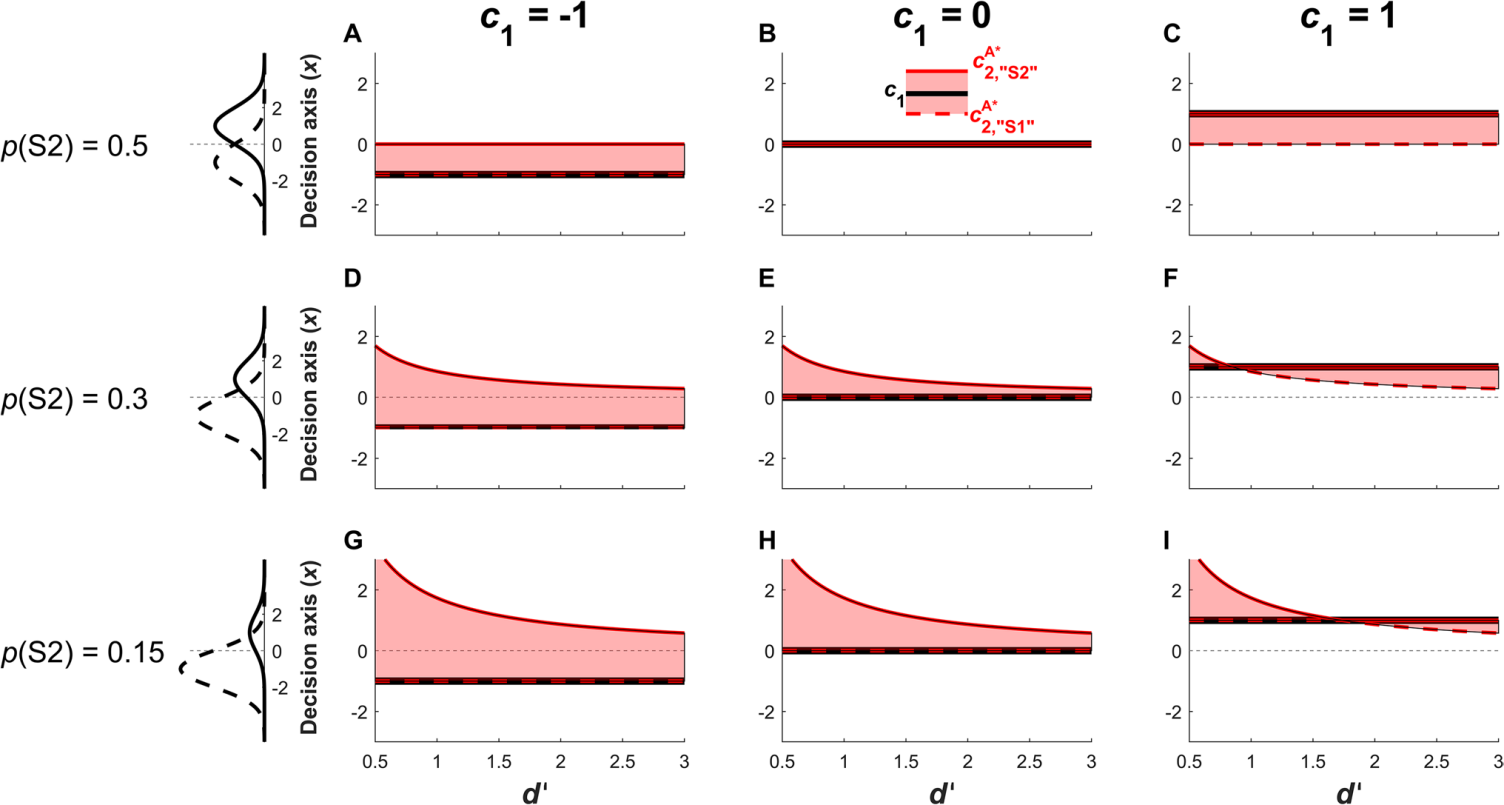
Positions of the optimal type 2 criteria ${c}_{2,{``\mathrm{S}1"}}^{\mathrm{A}^*}$ and ${c}_{2,{``\mathrm{S}2"}}^{\mathrm{A}^*}$ when maximizing type 2 accuracy as a function of *d’* (x-axis), for different type 1 criteria *c*_1_ (columns) and stimulus probabilities (rows). The left column depicts joint distribution functions *f*(*x*, S1) and *f*(*x*, S2) to better illustrate the influence of changing priors on optimal criterion setting, as discussed in the legend for Figure 2. The red shaded areas in **panels A-I** represent the region of the decision axis in which participants will respond “low confidence”. The optimal type 2 criteria ${c}_{2,{``\mathrm{S}1"}}^{\mathrm{A}^*}$ (red dashed line) and ${c}_{2,{``\mathrm{S}2"}}^{\mathrm{A}^*}$ (red solid line) are shown on each side of the type 1 criterion *c*_1_ (black solid line). When the type 1 criterion optimizes type 1 accuracy (i.e, ${c}_{1} ={c}_{1}^{\mathrm{A}^*}$) as in **(B)**, type 2 criteria are identical to the type 1 criterion and the observer never reports “low confidence”. When the type 1 criterion is suboptimal (all **panels** besides **B**), one of the optimal type 2 criteria is equal to the optimal type 1 criterion (${c}_{1}^{\mathrm{A}^*}$), while consistency constraints require the other type 2 criterion to be equal to the actual, suboptimal, type 1 criterion (*c*_1_).

Interestingly, ${c}_{2,{``\mathrm{S}2"}}^{\mathrm{A}^*}$ and ${c}_{2,{``\mathrm{S}1"}}^{\mathrm{A}^*}$ are virtually identical to the criterion that optimizes type 1 accuracy, ${c}_{1}^{\mathrm{A}^*}$ (Eq. 2). The only difference is in cases where the type 1 criterion is set suboptimally, thus preventing one of the type 2 criteria from equaling ${c}_{1}^{\mathrm{A}^*}$ due to the consistency constraints that ${c}_{{2,``\mathrm{S}2"}} \ge {c}_{1}$ and ${c}_{{2,``\mathrm{S}1"}} \le {c}_{1}$. For instance, when *p*(S1) = *p*(S2) = 0.5 (Figure 4, top row), the optimal criterion for type 1 accuracy is ${c}_{1}^{\mathrm{A}^*}=0$ (Figure 4B, black solid line), and the optimal type 2 criteria are also ${{c}_{2,{``\mathrm{S}1"}}^{\mathrm{A}^*}={c}_{{2,``\mathrm{S}2"}}^{\mathrm{A}^*}=0}$ (Figure 4B, red solid line). However, if priors are equal and the observer sets *c*_1_ = -1 (Figure 4A, black solid line), then the closest value to 0 that the optimal type 2 criterion for “S1” responses can achieve is ${c}_{2,{``\mathrm{S}1"}}^{\mathrm{A}^*}={c}_{1} = -1$ due to the consistency constraint that ${c}_{2,{``\mathrm{S}1"}}\le {c}_{1}$ (Figure 4A, red dashed line). Conversely, the optimal criterion for “S2” responses remains ${c}_{2,{``\mathrm{S}2"}}^{\mathrm{A}^*}=0$, and thus the observer reports low confidence for “S2” responses whenever -1 ≤ *x* ≤ 0 (red-shaded area between black and red lines).

Similar logic can be applied in the other plots of Figure 4. Of particular interest may be cases when *p*(S1) and *p*(S2) are not equal. When *p*(S1) ≠ *p*(S2), the optimal type 1 criterion ${c}_{1}^{\mathrm{A}^*}$ is inversely related to *d’* (Eq. 2), such that ${c}_{1}^{\mathrm{A}^*}$ takes on extreme values when *d’* is very small (with the sign and magnitude determined by the disparity in the priors), and asymptotes towards zero as *d’* approaches infinity (Figure 3). The optimal type 2 criteria ${c}_{2,{``\mathrm{S}2"}}^{\mathrm{A}^*}$ and ${c}_{2,{``\mathrm{S}1"}}^{\mathrm{A}^*}$ inherit this behavior (Figure 4, middle and bottom rows), subject to consistency constraints, such that one of the optimal type 2 criteria equals the *optimal* type 1 criterion ${c}_{1}^{\mathrm{A}^*}$, and the other equals the *actual* type 1 criterion *c*_1_.

Thus, provided that the observer sets the type 1 criterion optimally, i.e. so as to maximize type 1 accuracy, the observer maximizes type 2 accuracy by always reporting high confidence. If the observer sets the type 1 criterion suboptimally (e.g. by setting *c*_1_ = -1 when *p*(S1) = *p*(S2) as in Figure 4A), then type 2 accuracy is maximized by reporting low confidence only for evidence samples that fall between the optimal and actual type 1 criterion (e.g. evidence samples between the optimal ${c}_{1}^{\mathrm{A}^*}=0$ and the actual *c*_1_ = -1). We can understand this behavior by reference to the observation above that the criterion setting policy for optimizing type 1 accuracy (Eqs. 2-3) is equivalent to a Bayesian decision policy of responding “S2” when $\frac{p(\mathrm{S2}|x) }{p(\mathrm{S1}|x)}>1$, and responding “S1” otherwise. Regions of the decision axis lying between the *actual* and *optimal* type 1 criteria correspond to evidence samples where this decision policy is not used, i.e. cases where the observer responds “S2” in spite of S1 being the more probable stimulus (when the actual criterion *c*_1_ is more liberal than the optimal ${c}_{1}^{\mathrm{A}^*}$), or where the observer responds “S1” in spite of S2 being more probable (when the actual criterion *c*_1_ is more conservative than the optimal ${c}_{1}^{\mathrm{A}^*}$). On such trials, the observer’s classification response is actually more likely to be incorrect than correct. It follows that in order to maximize type 2 accuracy for these trials, the observer should report low confidence, since this strategy yields more type 2 correct rejections (low confidence incorrects) than the amount of type 2 hits (high confidence corrects) that could be accrued by reporting high confidence. Conversely, when the optimal response for a given evidence sample is given (all regions of the decision axis that do not lie between the actual *c*_1_ and optimal ${c}_{1}^{\mathrm{A}^*}$), then the response is more likely than not to be correct, meaning the observer should report high confidence since the expected frequency of type 2 hits from high confidence reports exceeds the expected frequency of type 2 correct rejections from low confidence reports.

In short, the optimal strategy for maximizing type 2 accuracy (Eqs. 6 and 8) is simply to report high confidence when the response is more likely than not to be correct (i.e. when the optimal type 1 response is given), and low confidence otherwise.

From this discussion, we can see that this notion of confidence rating is therefore somewhat redundant with type 1 decision making, since low confidence only indicates when a type 1 decision was made poorly (i.e. is likely to be incorrect). By contrast, in most experimental scenarios it is customary for ratings of low confidence to reflect cases where, although the decision is more likely than not to be correct, it nonetheless fails to surpass some more stringent criterion for reporting high confidence. However, this notion of confidence rating does map very naturally onto post-decision error detection (Boldt & Yeung, 2015; Charles & Yeung, 2019; Yeung & Summerfield, 2012), where a rating of low confidence would correspond to a report that the type 1 response was likely to be an error (Boldt & Yeung, 2015; Charles & Yeung, 2019).

### Optimizing reward

#### Optimal type 1 criterion for maximizing type 1 reward contingencies

Instead of optimizing task accuracy, an observer might have a different goal. Suppose an observer is presented with a reward matrix defining reward “points” (this could be e.g. money for a human subject, or drops of juice for a monkey, etc.) to be won or lost depending on the observer’s performance, as shown in Table 3. For instance, *R*_hit1_ corresponds to the number of points awarded following a type 1 hit trial. *R* values can be positive (typically for hits and correct rejections), zero, or negative (typically for misses and false alarms).

**Table 3:** Reward contingency table for type 1 responses. *R* indicates the points to be gained or lost under each response category

	type 1 payoff matrix	
event	classification	
	response = “S1”	response = “S2”
stimulus = S1	*R*_CR1_	*R*_FA1_
stimulus = S2	*R*_miss1_	*R*_hit1_

The observer’s objective is to gain as many points as possible. The measure to be optimized, expected reward for type 1 performance, can therefore be expressed as

9 $$\mathrm{E}\!\left(\mathrm{reward}_{1}\right)=p(\mathrm{S2})\left[{R}_\mathrm{hit1}\mathrm{HR}_{1}+{R}_\mathrm{miss1}(1-\mathrm{HR}_{1})\right]+p(\mathrm{S1})\left[{R}_\mathrm{CR1}(1-\mathrm{FAR}_{1})+{R}_\mathrm{FA1}\mathrm{FAR}_{1}\right]$$

with HR_1_ and FAR_1_ defined as above.

For convenience, let us define a relative reward quotient for type 1 reward, *Q*_1_, as

10 $${Q}_{1}= \frac{\left({R}_\mathrm{CR1}- {R}_\mathrm{FA1}\right)}{\left({R}_\mathrm{hit1}-{R}_\mathrm{miss1}\right)}$$

The value of *c*_1_ that maximizes E(reward_1_) is given by

11 $${c}_{1}^{\mathrm{R}^*}=\frac{\ln \frac{p(\mathrm{S1})}{p(\mathrm{S2})} + \ln {Q}_{1} }{d^{\prime}}={c}_{1}^{\mathrm{A}^*}+\frac{\ln {Q}_{1} }{d^{\prime}}$$

Thus, the criterion for maximizing expected type 1 reward (Eq. 11) is similar to the criterion for maximizing type 1 accuracy (Eq. 2), but with an added term *Q*_1_ (Eq. 10) that summarizes the relative balance of all possible type 1 reward outcomes.

The numerator of *Q*_1_, (*R*_CR1_ - *R*_FA1_), measures the relative reward for a correct vs an incorrect response when an S1 stimulus is shown. As this quantity increases, the optimal criterion becomes more conservative (Eq. 11) in order to make correct responses to S1 stimuli more likely and thereby reap the associated rewards. Conversely, the denominator of *Q*_1_, (*R*_hit1_ - *R*_miss1_), measures the relative reward for a correct vs an incorrect response when an S2 stimulus is shown. This quantity acts as a counterbalancing force in the criterion setting process, where increases tend to make the optimal criterion more liberal in order to increase reward from correct vs incorrect responses to S2 stimuli.

When stimulus priors are unequal (i.e., *p*(S1) ≠ *p*(S2)), Eq. 11 describes how consideration of stimulus priors trades off with consideration of reward contingencies. For instance, if S2 stimuli are twice as likely to occur as S1 stimuli, this would tend to make the optimal criterion more liberal to increase the frequency of “S2” responses. However, this consideration could be modulated or even overturned if the relative reward quotient *Q*_1_ favors “S1” responses strongly enough. For instance, if correct vs incorrect trials are rewarded three times as strongly for S1 stimuli (i.e. *Q*_1_ = 3), then the optimal criterion would actually be conservative, favoring “S1” responses in spite of S2 stimuli occurring twice as often (i.e. $\frac{p(\mathrm{S1})}{p(\mathrm{S2})}=\frac{1}{2}$).

In the special case where (*R*_CR1_ - *R*_FA1_) = (*R*_hit1_ - *R*_miss1_), the effects of relative reward contingencies for S1 and S2 stimuli cancel out and optimal criterion setting reduces to considering only the stimulus priors (Eq. 2).

Note that for ${c}_{1}^{\mathrm{R}^*}$ to take on a defined value that maximizes reward, it must be the case that (*R*_CR1_ - *R*_FA1_) > 0 and (*R*_hit1_ - *R*_miss1_) > 0. When either of (*R*_CR1_ - *R*_FA1_) or (*R*_hit1_ - *R*_miss1_) are zero or have different signs from each other, ${c}_{1}^{\mathrm{R}^*}$ is undefined. When (*R*_CR1_ - *R*_FA1_) and (*R*_hit1_ - *R*_miss1_) are both negative, the reward function E(reward_1_) is inverted and the equation for ${c}_{1}^{\mathrm{R}^*}$ picks out the minimum of the function rather than the maximum.

Derivation of ${c}_{1}^{\mathrm{R}^*}$ (Eq. 11) is provided in Supplementary Material S2, Section 2.1.

#### Optimal type 2 criteria for maximizing type 2 reward contingencies

It is possible that reward depends not only on type 1 performance (i.e. whether a response is a hit, miss, correct rejection, or false alarm), but also type 2 performance (i.e. whether those type 1 responses are endorsed with high or low confidence) (Fleming & Dolan, 2010; Lebreton et al., 2018; Locke et al., 2020). This gives a type 2 payoff matrix^5^, as shown in Table 4. *R*_hit2_, _FA2_, *R*_CR2_, and *R*_miss2_ correspond to the number of points following type 2 hits (high confidence corrects), type 2 false alarms (high confidence incorrects), type 2 correct rejections (low confidence incorrects), and type 2 misses (low confidence corrects). *R* values can be positive (typically for type 2 hits and correct rejections), zero, or negative (typically for type 2 misses and false alarms).

**Table 4:** Reward contingency table for type 2 responses. As before, *R* indicates the points to be gained or lost under each response category

	type 2 payoff matrix	
event	classification	
	confidence = low	confidence = high
accuracy = incorrect_1_	*R*_CR2_	*R*_FA2_
accuracy = correct_1_	*R*_miss2_	*R*_hit2_

Let us call reward in such a task reward_2_ to denote that it depends on type 2 performance (Table 4), as contrasted to reward under a payoff matrix that only depends on type 1 performance (Table 3). For “S2” responses, the observer must set the type 2 criterion ${c}_{2,{``\mathrm{S}2"}}$ so as to maximize E(reward_2,“S2”_):

12 $$\mathrm{E}\!\left(\mathrm{reward}_{2,\mathrm{``S2"}}\right) = p\!\left(\mathrm{hit}_{1},\mathrm{LC}|\mathrm{``S2"}\right){R}_\mathrm{miss2}+p\!\left(\mathrm{hit}_{1},\mathrm{HC}|\mathrm{``S2"}\right){R}_\mathrm{hit2} + p\!\left(\mathrm{FA}_{1},\mathrm{LC}|\mathrm{``S2"}\right){R}_\mathrm{CR2}+p\!\left(\mathrm{FA}_{1},\mathrm{HC}|\mathrm{``S2"}\right){R}_{\mathrm{FA2}}$$

For convenience, let us define a relative reward quotient for type 2 reward, *Q*_2_, as

13 $${Q}_{2}= \frac{\left({R}_{\mathrm{CR2}}-{R}_{\mathrm{FA2}}\right)}{\left({R}_\mathrm{hit2}-{ R}_\mathrm{miss2}\right)}$$

The value of *c*_2,“S2”_ that maximizes E(reward_2,“S2”_) is given by

14 $${c}_{2,{``\mathrm{S}2"}}^{\mathrm{R}^*}= \max\!\left(\frac{\ln \frac{p(\mathrm{S1})}{p(\mathrm{S2})} + \ln {Q}_{2} }{d^{\prime}},{c}_{1} \right)=\max \!\left({c}_{1}^{\mathrm{A}^*}+\frac{\ln {Q}_{2} }{d^{\prime}},{c}_{1} \right)$$

Similarly for “S1” responses, the observer must set *c*_2,“S1”_ so as to maximize E(reward_2,“S1”_):

15 $$\mathrm{E}\!\left(\mathrm{reward}_{2,{``\mathrm{S}1"}}\right)=p\!\left(\mathrm{CR}_{1},\mathrm{LC}|\mathrm{``S1"}\right){R}_\mathrm{miss2}+p\!\left(\mathrm{CR}_{1},\mathrm{HC}|\mathrm{``S1"}\right){R}_\mathrm{hit2}+p\!\left(\mathrm{miss}_{1},\mathrm{LC}|\mathrm{``S1"}\right){R}_\mathrm{CR2}+p\!\left(\mathrm{miss}_{1},\mathrm{HC}|\mathrm{``S1"}\right){R}_\mathrm{FA2}$$

The value of *c*_2,“S1”_ that maximizes E(reward_2,“S1”_) is given by

16 $${c}_{2,{``\mathrm{S}1"}}^{\mathrm{R}^*}=\min\!\left(\frac{\ln \frac{p(\mathrm{S1})}{p(\mathrm{S2})} + \ln \frac{1}{{Q}_{2}} }{d^{\prime}},{c}_{1} \right)=\min\!\left({c}_{1}^{\mathrm{A}^*}-\frac{\ln {Q}_{2} }{d^{\prime}},{c}_{1} \right)$$

Derivations of the type 2 criteria that optimize type 2 reward for “S2” and “S1” responses (${c}_{2,\mathrm{``S2"}}^{\mathrm{R}^*}$ and ${c}_{2,\mathrm{``S1"}}^{\mathrm{R}^*}$ via Eqs. 14 and 16, respectively) are provided in Supplementary Material S2, Sections 2.2.1 and 2.2.2.

Thus, the type 2 criteria for maximizing expected type 2 reward (${c}_{2,{``\mathrm{S}2"}}^{\mathrm{R}^*}$ and ${c}_{2,{``\mathrm{S}1"}}^{\mathrm{R}^*}$, Eqs. 14 and 16) are similar to the type 2 criteria for maximizing type 2 accuracy (${c}_{2,{``\mathrm{S}2"}}^{\mathrm{A}^*}$ and ${c}_{2,{``\mathrm{S}1"}}^{\mathrm{A}^*}$, Eqs. 6 and 8), but with an added term *Q*_2_ (Eq. 13) that summarizes the relative balance of all possible type 2 reward outcomes. The numerator of *Q*_2_, (*R*_CR2_ - *R*_FA2_), measures the relative reward for low vs high confidence when the classification response is incorrect, whereas the denominator (*R*_hit2_ - *R*_miss2_) measures relative reward for high vs low confidence when the response is correct. Thus, *Q*_2_ quantifies the tradeoff in reward for avoiding high confidence errors (numerator) vs accruing high confidence correct responses (denominator).

As the numerator (*R*_CR2_ - *R*_FA2_) increases, the observer is increasingly rewarded for avoiding high confidence errors. This incentivizes the observer to set type 2 criteria more conservatively, i.e. farther away from the type 1 criterion. Numerically, this conservative bias manifests as an increase in the type 2 criterion for “S2” responses (Eq. 14) and as a decrease in the type 2 criterion for “S1” responses (Eq. 16). Conversely, as the denominator (*R*_hit2_ - *R*_miss2_) increases, the observer is increasingly rewarded for accruing high confidence correct responses. This incentivizes the observer to set type 2 criteria more liberally, i.e. closer to the type 1 criterion. Numerically, this liberal bias manifests as a decrease in the type 2 criterion for “S2” responses (Eq. 14) and as an increase in the type 2 criterion for “S1” responses (Eq. 16).

**Fig. 5:**
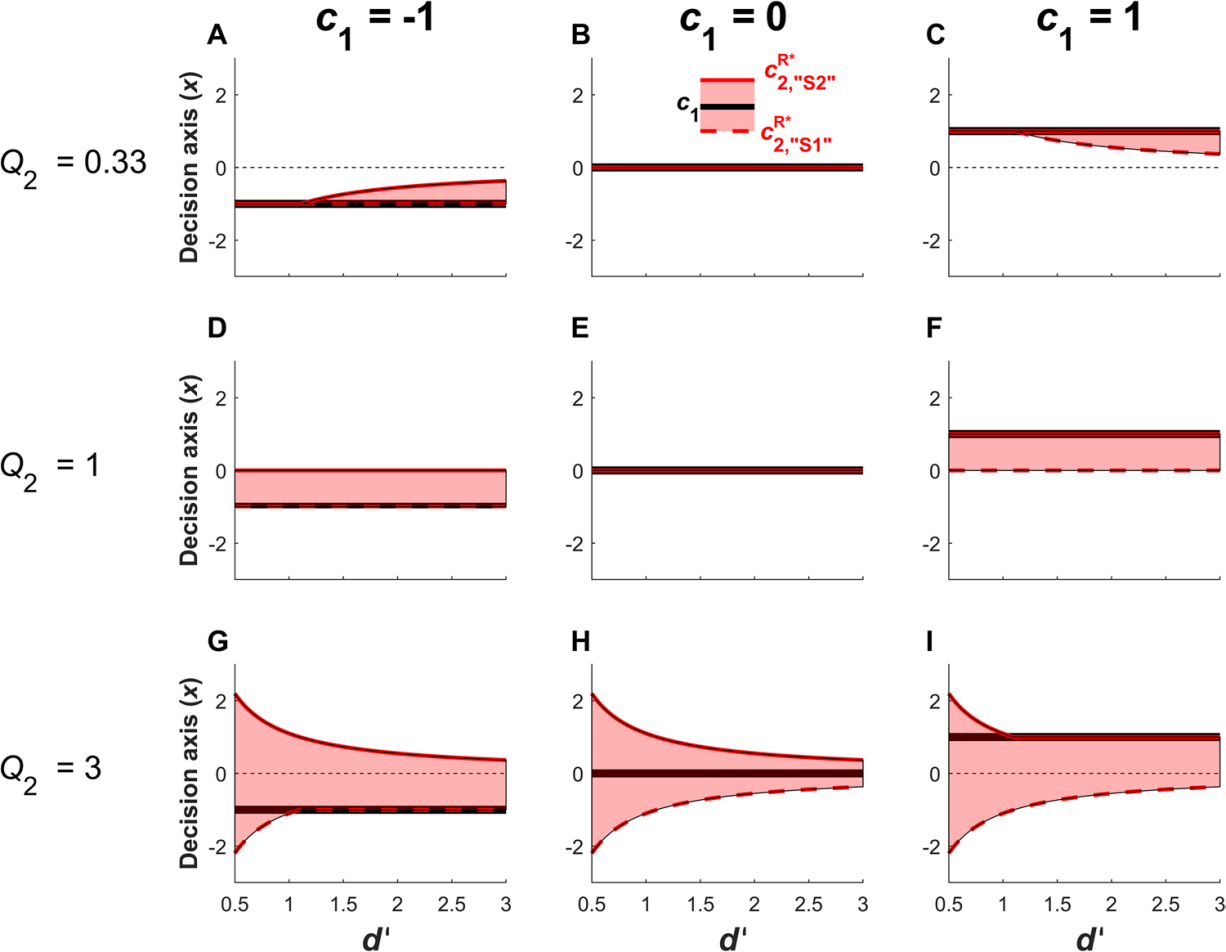
Positions of the optimal type 2 criteria ${c}_{2,{``\mathrm{S}1"}}^{\mathrm{R}^*}$ and ${c}_{2,{``\mathrm{S}2"}}^{\mathrm{R}^*}$ when maximizing type 2 reward (reward_2_, see main text), according to type 1 *d’* (x-axis) for different relative reward quotients *Q*_2_ (Eq. 13; rows) and different type 1 criteria (columns) under equal stimulus priors (i.e. *p*(S1) = *p*(S2) = 0.5). The red shaded areas represent the region in which an observer will report low confidence. The optimal type 2 criteria ${c}_{2,{``\mathrm{S}1"}}^{\mathrm{R}^*}$ (red dashed line) and ${c}_{2,{``\mathrm{S}2"}}^{\mathrm{R}^*}$ (red solid line) are shown on each side of the type 1 criterion *c*_1_ (black solid line). When *Q*_2_ < 1 **(A-C)**, accrual of high confidence corrects is incentivized more than avoidance of high confidence errors, leading to a liberal bias in type 2 criterion setting. When *Q*_2_ = 1 **(D-F)**, there is equal incentive to accrue high confidence corrects and avoid high confidence errors, and optimal type 2 criterion setting reduces to optimizing type 2 accuracy (compare to Figure 4A-C). When *Q*_2_ > 1 **(G-I)**, avoidance of high confidence errors is more strongly incentivized, leading to a conservative bias in type 2 criterion setting

In the special case where *Q*_2_ = 1 and (*R*_hit2_ - *R*_miss2_) = (*R*_CR2_ - *R*_FA2_), the effects of relative type 2 reward contingencies for correct and error trials cancel out and optimal criterion setting for maximizing type 2 reward reduces to optimal criterion setting for maximizing type 2 accuracy (i.e. Eqs. 14 and 16 reduce to Eqs. 6 and 8; compare Figure 4A-C to Figure 5D-F). Thus when *Q*_2_ = 1, the observer reports high confidence whenever their type 1 response is more likely than not to be correct. It follows that when *Q*_2_ < 1, the observer is incentivized to set their type 2 criteria even more liberally than in the *Q*_2_ = 1 case, meaning that even some responses that are likely to be *in*correct will be endorsed with high confidence. For instance, the shaded regions in Figure 5D and 5F are regions where responses are likely to be incorrect due to suboptimal type 1 criterion setting; the portion of these regions that are no longer shaded in Figure 5A and 5C are regions where setting ${Q}_{2}={~}^{1}\!\left/ \!{~}_{3}\right.$ incentivizes the observer to report high confidence for these likely incorrect responses. Thus, in practice, the maximization of type 2 reward is most interesting and relevant to typical confidence rating behavior when *Q*_2_ > 1 (Figure 5G-I), since here the observer is incentivized to report low confidence even for some responses that are likely to be correct. (See Section "Calibrating confidence to expected accuracy" for a fuller quantitative account of how *Q*_2_ relates to expected accuracy.)

Note that for ${c}_{2,{``\mathrm{S}2"}}^{\mathrm{R}^*}$ to take on a defined value that maximizes reward, it must be the case that (*R*_CR2_ - *R*_FA2_) > 0 and (*R*_hit2_ - *R*_miss2_) > 0. When either of (*R*_CR2_ - *R*_FA2_) or (*R*_hit2_ - *R*_miss2_) are zero or have different signs from each other, ${c}_{2,{``\mathrm{S}2"}}^{\mathrm{R}^*}$ is undefined. When (*R*_CR2_ - *R*_FA2_) and (*R*_hit2_ - *R*_miss2_) are both negative, the reward function E(reward_2,“S2”_) is inverted and the equation for ${c}_{2,{``\mathrm{S}2"}}^{\mathrm{R}^*}$ picks out the minimum of the function rather than the maximum. Similar reasoning applies to ${c}_{2,{``\mathrm{S}1"}}^{\mathrm{R}^*}$.

### Calibrating confidence to expected accuracy

Suppose the observer wishes to place high confidence ratings to reflect that some benchmark of type 1 accuracy has been achieved. For instance, the observer may choose to rate “high confidence” only when the estimated probability of a correct type 1 choice, *p*(correct_1_), exceeds 0.8. Here we name this objective “calibration,” taking inspiration from classic metacognition research in which calibration referred to how well an observer’s estimated accuracy predicted their actual accuracy (Koriat et al., 1980; Lichtenstein et al., 1977; Wagenaar, 1988). In the context of SDT, a well-calibrated observer is one who can accurately assess the location on the decision axis at which a given level of type 1 accuracy is achieved and use that knowledge to guide type 2 criterion setting. This type of goal may occur in the context of learning if confidence is to be used as a proxy for reward prediction error in the absence of external feedback, for example the work of Guggenmos and colleagues (2016) and Ptasczynski and colleagues (2021). How should the observer set type 2 criteria so as to achieve this goal?

More formally, let *p*(correct_1_)_T_ be the threshold value of accuracy needed to report high confidence (where T denotes threshold), and let *p*(correct_1_|*x*) be the observer’s estimate of being correct on the current trial based upon the evidence sample *x*. Then the observer seeking to calibrate confidence to accuracy uses the following decision policy for rating confidence:

17 $$\text{confidence} = \begin{cases} \text{high}, & \text{if } \: p(\mathrm{correct}_{1}|x)>p(\mathrm{correct}_{1})_\mathrm{T} \\ \text{low}, & \text{if } \: p(\mathrm{correct}_{1}|x)\le p(\mathrm{correct}_{1})_\mathrm{T}\end{cases}$$

As before, we consider “S1” and “S2” responses separately. On trials where the observer responds “S2,” the quantity the observer must consider in order to calibrate confidence to accuracy is *p*(correct_1,“S2”_|*x*), where *p*(correct_1,“S2”_) denotes probability of a correct type 1 response, given that the response was “S2”. Following Eq. 17, the observer must report high confidence for “S2” responses when *p*(correct_1,“S2”_|*x*) > *p*(correct_1_)_T_. This objective can be accomplished by setting the type 2 criterion *c*_2,“S2”_ at the location of the decision axis value *x* where *p*(correct_1,“S2”_|*x*) = *p*(correct_1_)_T_ .

For convenience, let us define *O*_T_ as the odds of a correct response at the threshold level of accuracy, *p*(correct_1_)_T_:

18 $${O}_\mathrm{T}= \frac{p(\mathrm{correct}_{1})_\mathrm{T}}{1-p(\mathrm{correct}_{1})_\mathrm{T}}$$

Then the value of *c*_2*,“*S2*”*_ that satisfies *p*(correct_1,“S2”_| *x*=*c*_2,“S2”_) ﻿= *p*(correct_1_)_T_ is given by

19 $${c}_{2,{``\mathrm{S}2"}}^{\mathrm{C}^*}=\max\!\left(\frac{\ln \frac{p(\mathrm{S1})}{p(\mathrm{S2})} + \ln {O}_\mathrm{T} }{d^{\prime}},{c}_{1} \right)=\max\!\left({c}_{1}^{\mathrm{A}^*}+\frac{\ln {O}_\mathrm{T} }{d^{\prime}},{c}_{1} \right)$$

Similarly for “S1” responses,

20 $${c}_{2,{``\mathrm{S}1"}}^{\mathrm{C}^*} = \min\!\left(\frac{\ln \frac{p(\mathrm{S1})}{p(\mathrm{S2})} + \ln \frac{1}{{O}_\mathrm{T}} }{d^{\prime}},{c}_{1} \right) = \min\!\left({c}_{1}^{\mathrm{A}^*}-\frac{\ln {O}_\mathrm{T} }{d^{\prime}},{c}_{1} \right)$$

Derivations of the type 2 criteria that optimize calibration for “S2” and “S1” responses (${c}_{2,{``\mathrm{S}2"}}^{\mathrm{C}^*}$ and ${c}_{2,{``\mathrm{S}1"}}^{\mathrm{C}^*}$ via Eqs. 19 and 20, respectively) are provided in Supplementary Material S2, Sections 3.2.1 and 3.2.2.

Thus, the type 2 criteria for optimizing calibration (Eqs. 19 and 20) are similar to the type 2 criteria for maximizing type 2 accuracy (Eqs. 6 and 8), but with an added term *O*_T_ (Eq. 18) corresponding to the odds of a correct response at the threshold level of accuracy, *p*(correct_1_)_T_.

When *p*(correct_1_)_T_ = 0.5, *O*_T_ = 1 and optimal criterion setting for calibration reduces to optimal criterion setting for maximizing type 2 accuracy (i.e. Eqs. 19 and 20 reduce to Eqs. 6 and 8). This is in agreement with earlier observations that maximizing type 2 accuracy is equivalent to reporting high confidence whenever the type 1 response is more likely than not to be correct.

As *p*(correct_1_)_T_ increases, the observer becomes increasingly stringent in what level of type 1 accuracy they will endorse with high confidence. This corresponds to setting type 2 criteria more conservatively, i.e. further away from the type 1 criterion. Numerically, this conservative bias manifests as an increase in the optimal type 2 criterion for “S2” responses ${c}_{2,{``\mathrm{S}2"}}^{\mathrm{C}^*}$ (Eq. 19) and as a decrease in the optimal type 2 criterion for “S1” responses ${c}_{2,{``\mathrm{S}1"}}^{\mathrm{C}^*}$ (Eq. 20).

The equations for the type 2 criteria that optimize calibration (${c}_{2,{``\mathrm{S}2"}}^{\mathrm{C}^*}$ and ${c}_{2,{``\mathrm{S}1"}}^{\mathrm{C}^*}$ , Eqs. 19 and 20) bear a close resemblance to the equations for those that optimize type 2 reward (${c}_{2,\mathrm{``S2"}}^{\mathrm{R}^*}$ and ${c}_{2,\mathrm{``S1"}}^{\mathrm{R}^*}$ , Eqs. 14 and 16), with the relative reward quotient *Q*_2_ (Eq. 13) playing the same role in type 2 criterion setting for optimizing type 2 reward as the odds of a correct response at the threshold level of accuracy *O*_T_ (Eq. 18) plays in type 2 criterion setting for optimizing calibration. In particular, increasing reward-based incentives to avoid high confidence errors (*Q*_2_) is equivalent to increasing the threshold level of accuracy for reporting high confidence (*O*_T_); in both cases, the ideal observer achieves their objective by becoming more conservative in reporting high confidence.

More generally, the equations for optimal type 2 criterion setting under reward maximization and calibration reveal a simple numerical and conceptual correspondence between these seemingly distinct strategies. For instance, the optimal strategy for type 2 criterion setting when avoidance of high confidence errors is rewarded twice as much as accrual of high confidence corrects (*Q*_2_ = 2) is equivalent to the optimal strategy for calibrating confidence reports such that high confidence trials are at least twice as likely to be correct as low confidence trials (*O*_T_ = 2, or equivalently *p*(correct_1_)_T_ = 2/3). An example of this equivalence is illustrated in Figure 2B-C.

**Fig. 6.**
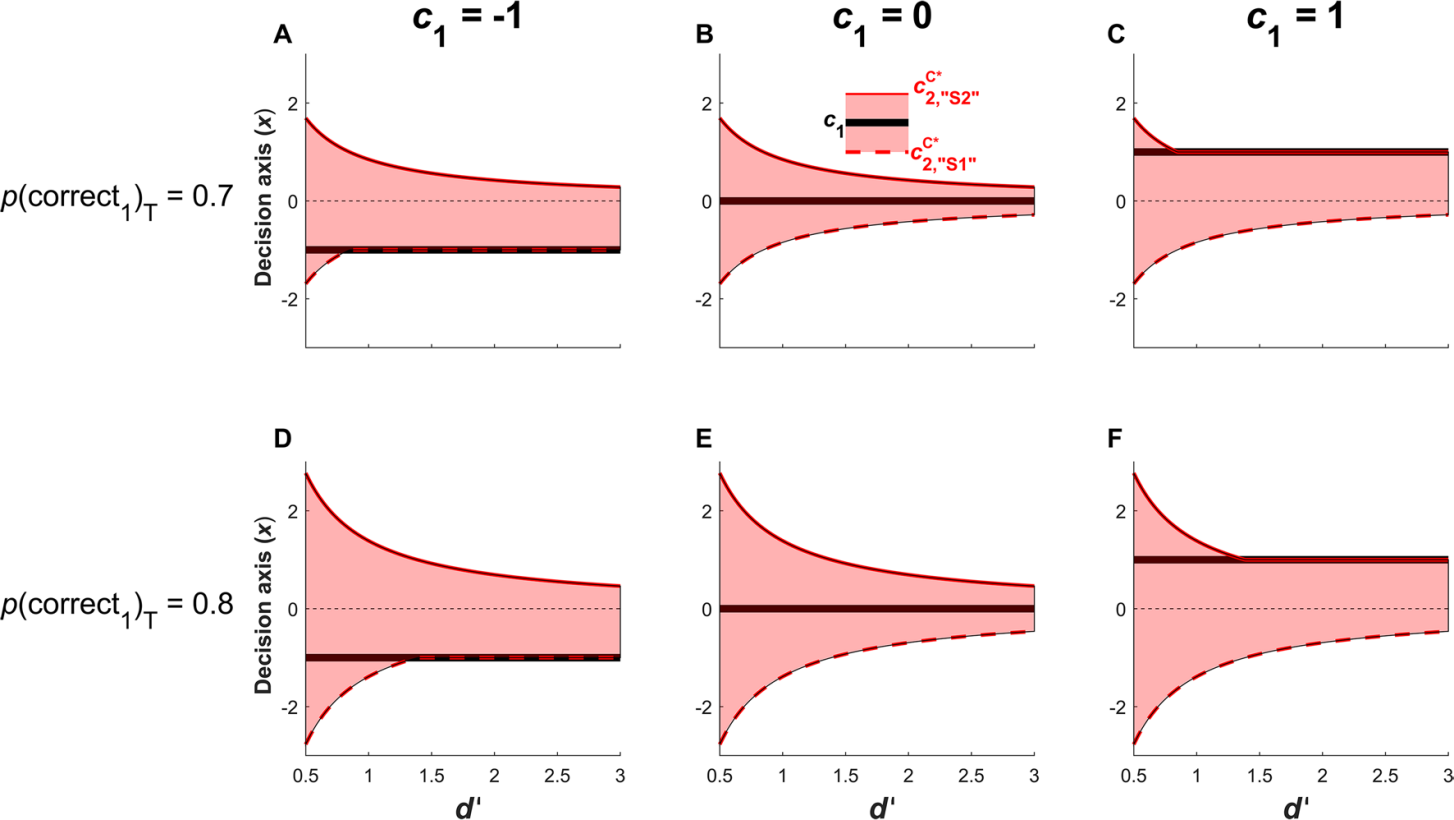
Positions of the optimal type 2 criteria ${c}_{2,{``\mathrm{S}1"}}^{\mathrm{C}^*}$ and ${c}_{2,{``\mathrm{S}2"}}^{\mathrm{C}^*}$ when calibrating confidence to a threshold level of accuracy *p*(correct_1_)_T_, according to type 1 *d’* (x-axis), for different values of *p*(correct_1_)_T_ (0.7 and 0.8; rows) and type 1 criterion (columns) under equal stimulus priors (i.e. *p*(S1) = *p*(S2) = 0.5). The red shaded areas represent the region in which the observer reports low confidence. The optimal criteria ${c}_{2,{``\mathrm{S}1"}}^{\mathrm{C}^*}$ (red dashed line) and ${c}_{2,{``\mathrm{S}2"}}^{\mathrm{C}^*}$ (red solid line) are shown on each side of the type 1 criterion *c*_1_ (black solid line)

Interestingly, as observed in Figure 6, the position of ${c}_{2,{``\mathrm{S}2"}}^{\mathrm{C}^*}$ and ${c}_{2,{``\mathrm{S}1"}}^{\mathrm{C}^*}$ is highly dependent on type 1 accuracy. Therefore, even when *p*(correct_1_)_T_ is set to a low value such as *p*(correct_1_)_T_ = 0.7 (Figure 6A-C), an observer will very rarely report high confidence when their *d’* is low (e.g. *d’* = 0.5). Likewise, when an observer has very high sensitivity (e.g. *d’* = 3), they will almost always report high confidence.

### Maximizing the difference between hit rate and false alarm rate

#### Optimal type 1 criterion for maximizing HR_1_ - FAR_1_

In the type 1 task, maximizing the difference between type 1 hit rate and false alarm rate is trivially accomplished by setting ${c}_{1} ={c}_{1}^{\mathrm{HF}^*}=0$ (see Supplementary Material S2, Section 4.1 for derivation).

Unlike the optimal type 1 criteria for maximizing accuracy (${c}_{1}^{\mathrm{A}^*}$, Eq. 2) and reward (${c}_{1}^{\mathrm{R}^*}$, Eq. 11), ${c}_{1}^{\mathrm{HF}^*}$ does not depend on stimulus priors or *d’*. This is because HR_1_ and FAR_1_ are rates of responding “S2” *conditional upon* presentation of S2 and S1 stimuli, respectively. As a result, the outcome measure HR_1_ - FAR_1_ is independent of stimulus priors, which is not the case with type 1 accuracy (Eq. 1) or reward (Eq. 9). Since HR_1_ - FAR_1_ does not depend on stimulus priors, it follows that the equation for the type 1 criterion that maximizes HR_1_ - FAR_1_ does not need to take priors into account.

#### Optimal type 2 criteria for maximizing HR_2_ - FAR_2_

Suppose the observer has the objective of maximizing the difference between type 2 hit rate and type 2 false alarm rate:

21 $${D}_{2}=\mathrm{HR}_{2}-\mathrm{FAR}_{2}$$

For “S2” responses, this is accomplished by setting

22 $${c}_{2,{``\mathrm{S}2"}}^{\mathrm{HF}^*}=\max\!\left(\frac{\ln \frac{\mathrm{HR}_{1}}{\mathrm{FAR}_{1}} }{d^{\prime}},{c}_{1} \right)$$

Similarly for “S1” responses,

23 $${c}_{2,{``\mathrm{S}1"}}^{\mathrm{HF}^*}=\min\!\left(\frac{\ln \frac{1-\mathrm{HR}_{1}}{\mathrm{1-FAR}_{1}} }{d^{\prime}},{c}_{1} \right)$$

Derivations of the type 2 criteria that optimize HR_2_ - FAR_2_ for “S2” and “S1” responses (${c}_{2,\mathrm{``S2"}}^{\mathrm{HF}^*}$ and ${c}_{2,\mathrm{``S1"}}^{\mathrm{HF}^*}$ via Eqs. 22 and 23, respectively) are provided in Supplementary Material S2, Sections 4.2.1 and 4.2.2.

Optimizing HR_2_ - FAR_2_ differs from the previously considered type 2 criterion setting strategies in that it does not depend on the stimulus priors *p*(S1) and *p*(S2). In this respect it is similar to the type 1 criterion that maximizes HR_1_ - FAR_1_, which is also independent of stimulus priors (see Section "Optimal type 1 criterion for maximizing HR_1_ - FAR_1_"). In both cases, the reason for the independence from stimulus priors is the same: since the outcome measures being maximized (HR_2_ - FAR_2_ and HR_1_ - FAR_1_) do not depend on stimulus priors, the criterion setting strategy for maximizing these outcome measures does not have to take those priors into account.

**Fig. 7:**
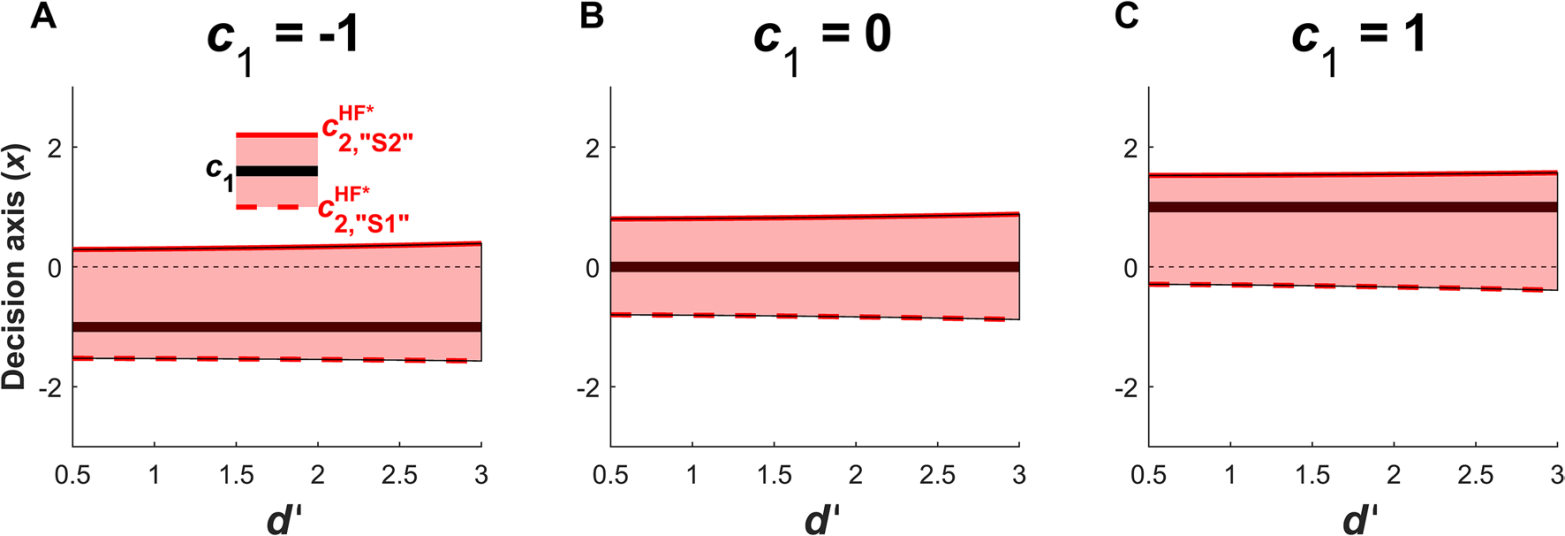
Positions of the optimal type 2 criteria ${c}_{2,{``\mathrm{S}1"}}^{\mathrm{HF}^*}$ and ${c}_{2,{``\mathrm{S}2"}}^{\mathrm{HF}^*}$ when maximizing the difference between type 2 hit and false alarm rates according to type 1 *d’* (x-axis), for different values of type 1 criterion *c*_1_ (columns). The red shaded areas represent the region in which the observer reports low confidence. The optimal criteria ${c}_{2,{``\mathrm{S}1"}}^{\mathrm{HF}^*}$ (red dashed line) and ${c}_{2,{``\mathrm{S}2"}}^{\mathrm{HF}^*}$ (red solid line) are shown on each side of the type 1 criterion *c*_1_ (black solid line)

As in the other optimization contexts, *d’* appears in the denominator of the equations for ${c}_{2,{``\mathrm{S}1"}}^{\mathrm{HF}^*}$ and ${c}_{2,{``\mathrm{S}2"}}^{\mathrm{HF}^*}$. However, the influence of *d’* on the optimal type 2 criteria is far more limited when maximizing HR_2_ - FAR_2_ than in the other optimization contexts (compare Figure 7 to Figures 4, 5, 6). The reason for this is that in the other optimization contexts, the numerator of the criteria equations contain terms that are fixed and independent of *d’* (*p*(S1), *p*(S2), *Q*_2_, *O*_T_). By contrast, for ${c}_{2,{``\mathrm{S}2"}}^{\mathrm{HF}^*}$ the numerator is ln(HR_1_) - ln(FAR_1_), whereas the denominator is *d’* = z(HR_1_) - z(FAR_1_) where z is the inverse of the normal cumulative distribution function. It turns out that for a fixed value of c_1_, the ratio of the functions ln(HR_1_) - ln(FAR_1_) and z(HR_1_) - z(FAR_1_) is approximately constant for typical values of *d’* in [0, 5] (which spans *p*(correct_1_) values from 0.5 to 0.99 for an unbiased observer). Similar considerations hold for ${c}_{2,{``\mathrm{S}1"}}^{\mathrm{HF}^*}$, where the numerator is ln(1 - HR_1_) - ln(1 - FAR_1_). Thus, ${c}_{2,{``\mathrm{S}1"}}^{\mathrm{HF}^*}$ and ${c}_{2,{``\mathrm{S}2"}}^{\mathrm{HF}^*}$ are sensitive to *c*_1_ but relatively independent of *d’* (Figure 7). These observations suggest a possible source for empirical observations suggesting a ‘fixed’ or immobile type 2 criterion across conditions or even tasks (Li et al., 2018; Rahnev et al., 2011; Solovey et al., 2015). See Section "Assessing the metacognitive strategies of real observers" for further discussion.

Another unique characteristic of ${c}_{2,{``\mathrm{S}1"}}^{\mathrm{HF}^*}$ and ${c}_{2,{``\mathrm{S}2"}}^{\mathrm{HF}^*}$ is that they are constrained to have negative and positive values, respectively. When *d’* > 0 it must be the case that HR_1_ > FAR_1_, which entails that ln(HR_1_ / FAR_1_) > 0 and thus ${c}_{2,{``\mathrm{S}2"}}^{\mathrm{HF}^*}$ > 0 (Eq. 22). By similar reasoning, when *d’* > 0 it must be the case that ${c}_{2,{``\mathrm{S}1"}}^{\mathrm{HF}^*}$ < 0. However, although they are constrained to have opposite signs, the two type 2 criteria can be located asymmetrically around 0 (Figure 7).

In the special case where stimulus priors are equal (i.e. *p*(S1) = *p*(S2)) and the observer sets the type 1 criterion optimally for maximizing type 1 accuracy (i.e. ${c}_{1} ={c}_{1}^{\mathrm{A}^*}=0$), maximizing HR_2_ - FAR_2_ is equivalent to calibrating confidence to the observer’s actual overall accuracy in the task, i.e. optimizing calibration when setting *p*(correct_1_)_T_ = *p*(correct_1_). For instance, compare the type 2 criteria for “S2” responses that optimize calibration (${c}_{2,{``\mathrm{S}2"}}^{\mathrm{C}^*}$, Eq. 19) and HR_2_ - FAR_2_ (${c}_{2,{``\mathrm{S}2"}}^{\mathrm{HF}^*}$, Eq. 22). Setting these equations equal when ${c}_{1}^{\mathrm{A}^*}=0$ implies $\frac{\mathrm{HR}_{1}}{\mathrm{FAR}_{1}}={O}_\mathrm{T}=\frac{p(\mathrm{correct}_{1})_\mathrm{T}}{1-p(\mathrm{correct}_{1})_\mathrm{T}}$. Furthermore, when *p*(S1) = *p*(S2) and ${c}_{1} ={c}_{1}^{\mathrm{A}^*}=0$, there is a symmetry whereby overall accuracy *p*(correct_1_) is identical to both hit rate HR_1_ and correct rejection rate (1 - FAR_1_), which in turn implies $\frac{\mathrm{HR}_{1}}{\mathrm{FAR}_{1}}=\frac{{p(\mathrm{correct}}_{1})}{1-p(\mathrm{correct}_{1})}$. Thus, in this special case, Eqs. 19 and 22 are identical when *p*(correct_1_)_T_ = *p*(correct_1_). Similar reasoning applies to “S1” responses. An example of this equivalence is illustrated in Figure 2C-D.

### The impact of suboptimal metacognition: When meta-*d’* < *d’*

The classical SDT model makes implicit predictions about metacognitive sensitivity since *d’* and *c*_1_ jointly imply type 2 ROC curves for “S1” and “S2” responses (Figure 1C-D). The measure called meta-*d’* inverts this relationship by characterizing empirical type 2 ROC curves in terms of the value of *d’* that maximizes their likelihood under classical SDT (Maniscalco & Lau, 2012, 2014). For instance, if meta-*d’* = 0.5, this means that the empirical type 2 ROC curves are close to what would be predicted by a classical SDT model in which *d’* = 0.5. As *d’* increases, so does the area under the SDT-predicted type 2 ROC curves, which is a measure of metacognitive sensitivity. Thus, meta-*d’* measures metacognitive sensitivity in terms of the *d’* that would be predicted from type 2 data by the SDT model. By definition, SDT predicts that meta-*d’* = *d’*, and when this prediction is satisfied we say that the observer’s type 2 sensitivity is ideal according to SDT, or SDT-ideal.

Thus far, we have only considered optimization of type 2 criteria when metacognitive sensitivity conforms to SDT expectation, i.e. when meta-*d’* = *d’*. However, empirically it is often the case that this expectation is violated, such that meta-*d’* ≠ *d’*. In most such cases meta-*d’* is smaller than *d’*, which indicates suboptimal metacognitive sensitivity relative to SDT expectation. Alternatively, it has also been found that meta-*d’* can be greater than *d’* (Charles et al., 2013) if time-pressure on the response is increased and participants are able to detect and correct a large proportion of their fast erroneous guesses. Therefore, an important question is how the derived optimal type 2 criteria may change in cases where metacognitive sensitivity cannot be adequately captured by the standard SDT model (i.e. cases where meta-*d’* ≠ *d’*).

When meta-*d’* ≠ *d’*, one might be tempted to simply substitute meta-*d’* for all occurrences of *d’* in the formulae for optimal type 2 criteria. However, this is not appropriate since the meta-*d'* framework is not intended to provide a model of the specific computational processes by which the SDT expectation that meta-*d’* = *d’* is violated. Rather, the meta-*d’* framework answers the counterfactual question, “what *d’* would a hypothetical SDT-ideal observer require in order to reproduce the observed type 2 ROC curves?” As a consequence, the type 2 criteria in the meta-*d’* model apply to this hypothetical SDT-ideal observer, not to the actual observer whose data is being analyzed. Thus, substituting meta-*d’* for *d’* in the above equations would inform us about how this hypothetical SDT-ideal observer should set their type 2 criteria in order to be optimal. However, it would not necessarily shed much light on optimal type 2 criterion setting for the actual observer whose data violates SDT expectation. Indeed, below we will show that even for a fixed *d’* value, optimal type 2 criteria can differ for the same value of meta-*d’*, depending on the computational processes whereby that meta-*d’* value was generated.

It is therefore necessary to first specify a model which makes explicit the computational mechanisms whereby meta-*d’* ≠ *d’* can occur, and then investigate how the behavior of that model influences optimal type 2 criterion setting. For instance, a simple example of such a process model (considered further below) is one that is identical to the standard SDT model, but with the added mechanism that the evidence samples used for type 1 judgments receive extra Gaussian noise before being used for type 2 judgments. However, even for this very simple extension of the standard SDT model, the mathematics for deriving optimal type 2 criteria become intractable^6^. Fortunately, optimal type 2 criterion setting under such models can be explored via computational simulation.

There are many possible models of how the SDT expectation that meta-*d’* = *d’* might be violated, and a comprehensive exploration of how every possible model that has been proposed in the literature might impact type 2 criterion setting is beyond the scope of this paper. Thus, here we use computational simulations to investigate two simple example models – a “type 2 noise” model and a “type 2 signal loss” model – for the sake of illustrating how different mechanisms that allow for meta-*d'* < *d'* might similarly and differentially impact optimal type 2 criterion setting (Maniscalco & Lau, 2016; Peters et al., 2017; Shekhar & Rahnev, 2021a, 2021b). Readers interested in exploring optimal type 2 criterion setting in alternative models are invited to adapt the code used for the simulations below to their own purposes. This simulation code is available online at https://github.com/CNClaboratory/opt_t2c and is designed to be easily used with any specified model of type 2 processes.

In the interest of brevity and exploring illustrative examples rather than being exhaustive, we also consider only two of the four optimization contexts explored above: maximizing type 2 reward and maximizing the difference between type 2 hit rate and type 2 false alarm rate. As seen above, the equations for optimizing type 2 accuracy, type 2 reward, and calibration in the traditional SDT model are similar, and thus we chose type 2 reward optimization as a representative optimization context from this set. For interested readers, the simulation code available online allows for exploration of optimal type 2 criterion setting for type 2 accuracy and calibration.

Our general approach to the simulations was to investigate how a representative range of values for the parameter controlling metacognitive sensitivity affected optimal type 2 criterion setting, holding all other parameters of the model constant as a fixed reference. Briefly, in the type 2 noise model (Section "Type 2 noise model"**;** Supplementary Material S3), the amount of extra noise added prior to the type 2 judgment is captured by the single parameter *σ*_2_; in the type 2 signal loss model (Section "Type 2 signal loss model"**; **Supplementary Material S3), it is assumed that prior to the type 2 judgment, the internal signal loses magnitude according to a scalar multiplier *k* (and also is corrupted by type 2 noise). We fixed other parameters at *d’* = 2, *c*_1_ = 0, and *p*(S2) = 0.5, and in the reward optimization simulations, we set *Q*_2_ = 3.

Here in the main text we provide a high-level summary of the results of these simulations to illustrate how even just these two different process models, under just these two optimization contexts, make qualitatively different predictions about shifts in the optimal type 2 criteria: Sometimes they should become more liberal under decreasing meta-*d’*, while other times they should become more conservative. Full details for all simulations and more comprehensive exploration of the results are provided in Supplementary Material S3.

#### Type 2 noise model

In the type 2 noise model, type 2 evidence samples are noisier than their type 1 counterparts, thus yielding suboptimal metacognitive sensitivity relative to SDT expectation. Specifically, the relationship between type 1 signal *x*_1_ and type 2 signal *x*_2_ on a given trial is given by

24 $${x}_{2}={x}_{1}+\varepsilon , \varepsilon \sim N(0,{{\sigma }_{2}}^{2})$$

where *σ*_2_ is the standard deviation of the additional type 2 noise, over and above any noise present at the type 1 level. In the absence of type 2 noise, when *σ*_2_ = 0, the model reduces to the standard SDT model described in Supplementary Material S1. As *σ*_2_ grows larger, metacognitive sensitivity (as measured by meta-*d’*) decreases.

#### Type 2 signal loss model

In the type 2 signal loss model, in addition to the presence of type 2 noise, the magnitude of the internal signal used to make type 2 judgments can also be smaller than its type 1 counterpart. Specifically, the relationship between type 1 signal *x*_1_ and type 2 signal *x*_2_ on a given trial is given by

25 $${x}_{2}=(1-k){x}_{1}+\varepsilon , \varepsilon \sim N(0,{{\sigma }_{2}}^{2})$$

where 0 ≤ *k* ≤ 1 is the signal loss parameter and *σ*_2_ is the standard deviation of the additional type 2 noise. In the absence of type 2 noise and signal loss, when *σ*_2_ = 0 and *k* = 0, the model reduces to the standard SDT model described in Supplementary Material S1. When *σ*_2_ > 0, increases in *k* yield reductions in meta-*d’*.^7^ Thus here we set *σ*_2_ to a small constant value of 0.1 and explore the effect of modulating *k* on optimal type 2 criterion setting.

#### Type 2 noise and signal loss differently affect optimal type 2 criterion setting

**Fig. 8:**
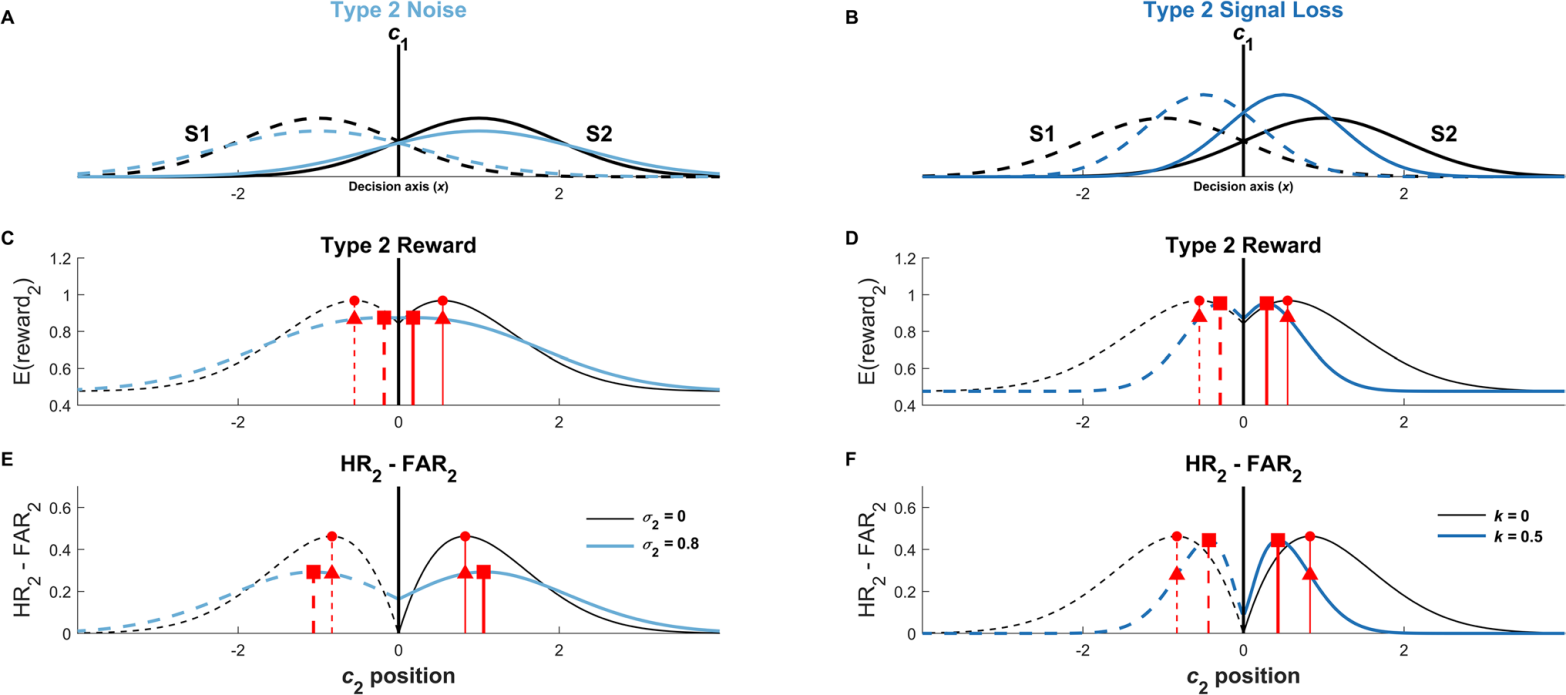
Example illustrations of how type 2 noise **(A,C,E)** and type 2 signal loss **(C,D,F)** affects optimal type 2 criterion setting by changing the distribution of the outcome measure to be optimized. **(A)** The standard signal detection theoretic framework is shown for reference, with values of *d’* = 2 and *c*_1_ = 0 (black lines). Blue lines depict the effect of type 2 noise **(A)** and type 2 signal loss **(B)** on the distributions. **(C-D)** Expected type 2 reward (given *Q*_2_ = 3 and *p*(S1) = *p*(S2) = 0.5) as a function of possible type 2 criterion values for “S1” responses (x-axis values lower than *c*_1_) and “S2” responses (x-axis values higher than *c*_1_) under added type 2 noise **(C)** and type 2 signal loss **(D)**. Red squares depict the expected outcome value for the optimal *c*_2_ if the observer were to take into account the presence of the type 2 noise **(C)** or signal loss **(D)**, while red triangles depict the expected outcome value for the optimal *c*_2_ if the observer were to ignore the presence of the type 2 noise or signal loss. **(E-F)** HR_2_ - FAR_2_ as a function of possible type 2 criterion values for “S1” responses (x-axis values lower than *c*_1_) and “S2” responses (x-axis values higher than *c*_1_) under added type 2 signal noise **(E)** and type 2 signal loss **(F)**. Red squares depict the expected HR_2_ - FAR_2_ for the optimal *c*_2_ if the observer were to take into account the presence of the type 2 noise **(E)** or signal loss **(F)**, while red triangles depict the expected outcome value for the optimal *c*_2_ if the observer were to ignore the presence of the type 2 noise or signal loss

In Figure 8 we provide an initial intuition for how type 2 noise (Figure 8A, C, E) and type 2 signal loss (Figure 8B, D, F) affect type 2 criterion setting, using an example where *d’* = 2, *c*_1_ = 0, and *p*(S2) = 0.5 (Figure 8A-B).

In a reward optimization context with *Q*_2_ = 3 (Figure 8C-D) and no type 2 noise (*σ*_2_ = 0) and no signal decay (*k* = 0), expected reward for each response type is a unimodal function of *c*_2_ position (Figure 8C-D, thin black lines), and the optimal type 2 criteria are the *c*_2_ values at which the maxima of these functions occur (circles, thin red lines). When type 2 noise is introduced (*σ*_2_ = 0.8, Figure 8C), the expected reward functions flatten out such that the maxima of the functions for each response type move inwards towards the type 1 criterion (Figure 8C, light blue lines). As a consequence, type 2 noise has the effect of making the optimal type 2 criteria for maximizing reward more liberal (thick red lines and red squares). Note that if the observer fails to take type 2 noise into account and sets their type 2 criteria according to the optimal strategy when *σ*_2_ = 0 (Figure 8C, thin red lines and red triangles), the loss in reward is relatively small (difference in y-value between red squares and red triangles). In the case of type 2 signal loss (*k* = 0.5, *σ*_2_ = 0.1, Figure 8D), the expected reward functions instead become sharper but the maxima still move inwards towards the type 1 criterion (Figure 8D, dark blue lines). Thus, under this optimization scheme, type 2 signal loss has a similar effect to type 2 noise, making the optimal type 2 criteria for maximizing reward more liberal (thick red lines and squares). Here, failing to take into account type 2 signal loss would lead to a slightly larger reduction in expected reward (Figure 8D, red squares vs red triangles).

In the HR_2_ - FAR_2_ optimization context, the effect of type 2 noise is again to flatten the expected outcome functions (Figure 8E, light blue lines), but in this case the result is that the maxima of the functions move outwards away from the type 1 criterion, not inwards; thus, the optimal type 2 criteria become more conservative (thick red lines and squares). Ignoring type 2 noise in this context leads to minimal reduction in the outcome function (Figure 8E, red squares vs red triangles). In contrast, the effect of type 2 signal loss (Figure 8F, dark blue lines) is still to sharpen the reward function and move it inward, such that the optimal type 2 criteria become more liberal. In this case, though, note that ignoring the presence of signal loss leads to a substantial reduction in the outcome function (Figure 8F, difference between red squares and red triangles).

A more comprehensive and systematic investigation of how type 2 noise and signal decay affect optimal type 2 criterion setting is provided in Supplementary Material S3. There, we demonstrate in more detail how the optimal type 2 criteria may become either more liberal or more conservative with decreasing metacognitive sensitivity, depending on the process model and the outcome measure to be optimized. We additionally explore how the cost (failure to achieve maximal outcome) of neglecting to take metacognitive suboptimality into account when setting type 2 criteria can be either quite small or large, depending on the process model.

**Fig. 9:**
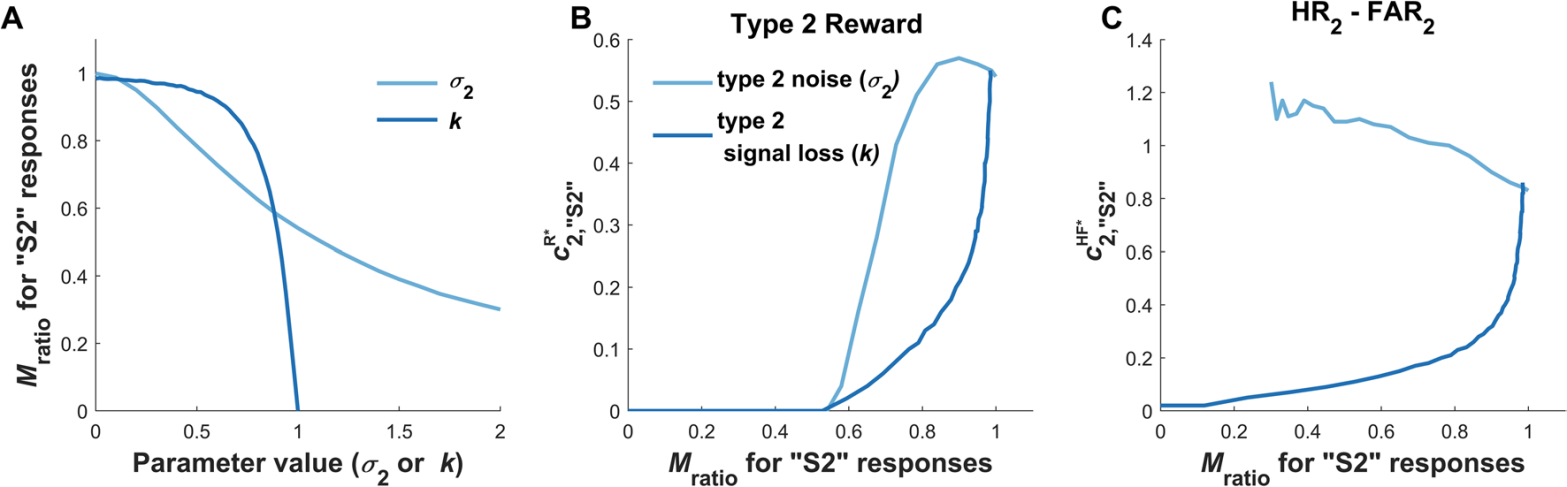
Effect of type 2 noise and signal loss parameters on *M*_ratio_ and on the position of the optimal confidence criterion ${c}_{2,{``\mathrm{S}2"}}^{*}$. **(A)** Effect of type 2 noise (*σ*_2_, light blue) and type 2 signal loss (*k*, dark blue) on *M*_ratio_. **(B-C)** Position of ${c}_{2,{``\mathrm{S}2"}}^{*}$ according to the *M*_ratio_ resulting from different levels of type 2 noise (*σ*_2_, light blue) and type 2 signal loss (*k*, dark blue), when maximizing type 2 reward **(B)** or HR_2_ - FAR_2_ **(C)**

The different behavior of the type 2 noise and type 2 signal loss models can be further illustrated by considering how optimal type 2 criterion setting depends on metacognitive efficiency, quantified by *M*_ratio_ (*M*_ratio_ = meta-*d’*/*d’*), according to each model. Whereas values of the type 2 noise and type 2 signal loss parameters σ_2_ and *k* are not directly comparable, each parameter value or combination entails a specific value of *M*_ratio_ (Figure 9A), and so the models can be directly compared at parameter values that yield identical *M*_ratio_ values (Figure 9B and C). This plotting method makes clear that the relationship between *M*_ratio_ and optimal type 2 criterion setting differs for the type 2 noise and type 2 signal loss models under the two optimization contexts considered here. More generally, this finding implies that the question of how optimal type 2 criterion setting is influenced by metacognitive sensitivity when meta-*d’* ≠ *d’* cannot be addressed without specifying a type 2 process model and optimization context.

The foregoing points underscore the importance of context – including the specified process model and outcome measure to be optimized – for characterizing optimal type 2 criterion setting when meta-*d’* ≠ *d’*. For simplicity, here we have not considered the impact of type 1 parameters *d'*, *c*_1_, and *p*(S2), but it is likely that these parameters introduce further interesting complexities in characterizing optimal type 2 criterion setting. The upshot of all this is that analyses intended to characterize optimal type 2 setting when meta-*d’* ≠ *d’* need to utilize theoretical or simulation-based approaches that are carefully tailored to the specifics of the empirical or theoretical phenomena under consideration. In Section "Analyzing type 2 criterion setting when meta-*d’* ≠ *d’*" we further explore the ramifications of these observations for the analysis of optimal type 2 criterion setting.

We note that the code for the above simulations is available in the opt_t2c toolbox that we provide as a companion piece to this manuscript (https://github.com/CNClaboratory/opt_t2c). This toolbox can be readily used to explore optimal type 2 criterion setting for any parameter settings of the type 2 noise and type 2 signal loss models, and for any of the four optimization contexts considered in this manuscript. It can also be readily adapted to explore the behavior of alternative process models.

## General discussion

An observer’s strategy for rating confidence in their decisions depends highly on that observer’s goal: just as with type 1 decisions, the optimal strategy for producing type 2 ratings of confidence changes whether the observer wishes to maximize type 2 accuracy, type 2 reward, the correspondence between confidence and accuracy, or the difference between type 2 hit and false alarm rates. In this paper, we explored the different strategies an observer can adopt to optimize these various outcomes using the framework of type 2 signal detection theory.

To facilitate this exploration and derivation of optimal strategies, we first reviewed how, according to classic signal detection theory, the decision criterion can be set to optimize a particular outcome measure following the experimenter’s instructions and stimuli presented. We then explored how such optimization could apply to confidence judgments, evaluating how different outcome measures at the type 2 level could be optimized, and how this would affect the position of the criteria for reporting high confidence. We considered four distinct optimization strategies (in the context of their type 1 counterparts as appropriate): (1) maximize type 2 accuracy, (2) maximize type 2 reward, (3) adjust confidence to a threshold probability of type 1 accuracy and (4) maximize the difference between type 2 hit rate and type 2 false alarm rate. For each outcome measure, we derived mathematical formulae for computing the optimal type 2 criteria in the standard signal detection theory model. Finally, we used computational simulations to conduct a preliminary exploration of how optimal type 2 criterion setting changes when metacognitive sensitivity deviates from SDT expectation, i.e. models in which type 2 signal is altered compared to type 1 signal (which entails meta-*d’* ≠ *d’*).

For simplicity, all analyses here have assumed the equal variance SDT model, which is appropriate for discrimination and 2AFC tasks. However, detection tasks are often better modeled by the unequal variance SDT model, and so some caution is warranted in applying the present analyses to detection tasks (Green & Swets, 1966; Macmillan & Creelman, 2004). Future work can readily extend the theoretical and simulation-based approaches in this paper to the unequal variance case.

### Summary of results

Interestingly, we found several formal similarities and conceptual links among the type 2 criteria for maximizing type 2 accuracy, maximizing type 2 reward, and calibrating confidence to a threshold level of accuracy. All of these have a close connection to the type 1 criterion that maximizes type 1 accuracy (${c}_{1}^{\mathrm{A}^*}$), being located either as close as possible to ${c}_{1}^{\mathrm{A}^*}$ (for type 2 accuracy) or symmetrically around ${c}_{1}^{\mathrm{A}^*}$ (for type 2 reward and calibration), subject to consistency constraints. The type 2 criteria for maximizing type 2 reward and calibrating to accuracy additionally depend on the observer’s type 1 sensitivity (*d'*) and relevant aspects of the decision making context (the reward quotient *Q*_2_ and threshold accuracy odds ratio *O*_T_, respectively, which play formally identical roles in their respective contexts). Conversely, the type 2 criteria for maximizing the difference between type 2 hit and false alarm rate behave qualitatively differently in that they are almost completely independent from the optimal type 1 criterion and *d’*, instead depending primarily on the observer’s actual type 1 criterion. We further show that the calibration context can be useful for shedding light on the other optimization contexts by characterizing their confidence behavior in terms of accuracy thresholds.

We also found that characterizations of optimal type 2 criterion setting must take metacognitive sensitivity into account. Specifically, we used simulations to show that when metacognitive sensitivity deviates from SDT expectation, the truly optimal type 2 criteria may differ from the values predicted by the equations derived from the classical SDT framework. The specific manner in which this occurs depends upon the optimization context and the underlying computational model of how type 2 responses are generated, even at matched levels of metacognitive efficiency. We discuss and expand upon the significance of each of these findings in the next sections.

#### Optimizing type 2 accuracy

The most common target for optimizing type 1 task performance is to maximize accuracy. It is therefore natural to consider the objective of maximizing the analogue of accuracy in the type 2 confidence rating task (Table 2), where “correct” type 2 trials are those in which confidence is congruent with accuracy (high confidence for correct trials and low confidence for incorrect trials).

Derivation of the optimal type 2 criteria for maximizing type 2 accuracy (${c}_{2,{``\mathrm{S}1"}}^{\mathrm{A}^*}$ and ${c}_{2,{``\mathrm{S}2"}}^{\mathrm{A}^*}$) reveals that they are closely related to the optimal type 1 criterion for maximizing type 1 accuracy (${c}_{1}^{\mathrm{A}^*}$): the optimal strategy is essentially to place the type 2 criteria as close as possible to the *optimal* type 1 criterion, subject to consistency constraints requiring the type 2 criteria for “S1” and “S2” responses to be located on the appropriate sides of the *actual* type 1 criterion (Eqs. 6 and 8). Ultimately, this entails that the observer should only report low confidence for suboptimal type 1 responses (see Section "Optimal type 2 criteria for maximizing type 2 accuracy" for full reasoning). A suboptimal type 1 response occurs when the evidence sample falls between the *actual* and *optimal* type 1 criterion, in which case the response is more likely than not to be incorrect (Figure 4). It follows that in order to maximize type 2 accuracy, confidence rating should be treated like an error detection task in which low confidence ratings flag likely errors. When the observer sets the type 1 criterion optimally, responses are never more likely to be incorrect than correct, and thus the ideal strategy is to always report high confidence.

#### Optimizing type 2 reward

When hits, misses, correct rejections, and false alarms in the type 1 task are associated with different rewards (Table 3), the observer may seek to respond in a way that maximizes reward rather than accuracy. The objective to maximize reward can also influence type 2 criterion setting in cases where reward depends on type 2 hits, misses, correct rejections, and false alarms (Table 4). We call reward contingent upon type 2 outcomes “type 2 reward.”

The optimal type 2 criteria for maximizing type 2 reward (${c}_{2,{``\mathrm{S}1"}}^{\mathrm{R}^*}$ and ${c}_{2,{``\mathrm{S}2"}}^{\mathrm{R}^*}$) are located symmetrically around the optimal type 1 criterion that maximizes type 1 accuracy (${c}_{1}^{\mathrm{A}^*}$), subject to consistency constraints (Eqs. 14 and 16). Their distance from ${c}_{1}^{\mathrm{A}^*}$ depends on the relative reward quotient *Q*_2_ (Eq. 13), which measures the ratio of the reward for avoiding high confidence errors (*R*_CR2_ – *R*_FA2_) to the reward for accruing high confidence correct responses (*R*_hit2_ – *R*_miss2_).

When the quotient *Q*_2_ = 1, optimizing type 2 reward reduces to optimizing type 2 accuracy, and when *Q*_2_ < 1, the observer is incentivized to be excessively liberal, such that even some responses that are likely to be incorrect should be endorsed with high confidence. Thus, the case where rewards incentivize the observer to avoid high confidence errors (*Q*_2_ > 1) is of the greatest interest. In this scenario, the observer should report low confidence for trials that are likely to be correct but are nonetheless supported by relatively weak supporting evidence, consistent with how confidence ratings are typically employed in metacognition research. Another way of expressing this observation is that setting *Q*_2_ > 1 is equivalent to setting *O*_T_ > 1 in the calibration framework, i.e. setting the threshold level of accuracy for reporting high confidence greater than the chance level of accuracy 0.5; see following section.

When the reward quotient encourages conservative type 2 criterion setting (*Q*_2_ > 1), the extent of this conservative influence is moderated by the observer’s type 1 sensitivity (*d’*) (Figure 5). For a fixed reward quotient, the observer should be more conservative in reporting high confidence when *d’* is low and errors are more frequent, so as to mitigate the risk of high confidence errors. Conversely, the observer should be more liberal when *d’* is high and errors are more infrequent, so as to safely capitalize on the opportunity for high confidence correct responses.

#### Calibrating confidence to accuracy

A natural confidence rating strategy is to report high confidence whenever the estimated likelihood that one’s response is correct surpasses some threshold level of accuracy, *p*(correct_1_)_T_. For instance, one might report high confidence whenever one estimates that one’s response is at least 80% likely to be correct. In this case, the optimal type 2 criterion setting strategy does not involve maximizing some outcome measure, but rather consists in accurately assessing the location on the decision axis corresponding to the desired threshold accuracy level.

The optimal type 2 criteria for calibrating confidence to a threshold probability of being correct *p*(correct_1_)_T_ (${c}_{2,{``\mathrm{S}1"}}^{\mathrm{C}^*}$ and ${c}_{2,{``\mathrm{S}2"}}^{\mathrm{C}^*}$ ) are located symmetrically around the optimal type 1 criterion (${c}_{1}^{\mathrm{A}^*}$), subject to consistency constraints (Eqs. 19 and 20). Their distance from ${c}_{1}^{\mathrm{A}^*}$ depends on the odds ratio of *p*(correct_1_)_T_ to (1 - *p*(correct_1_)_T_), denoted *O*_T_ (Eq. 18). The higher one sets *p*(correct_1_)_T_, the higher the estimated accuracy of a particular response must be in order to report high confidence, leading to increasingly conservative biases in confidence rating. When *p*(correct_1_)_T_ = 0.5, the equations for the optimally calibrating type 2 criteria reduce to the equations for optimizing type 2 accuracy. This stands in agreement with the above observation that when optimizing type 2 accuracy, one reports high confidence whenever the response is more likely than not to be correct, i.e. when *p*(correct_1_) > 0.5.

The equations for calibrating type 2 criteria to accuracy (Eqs. 19 and 20) are remarkably similar to the equations for the type 2 criteria that optimize reward (Eqs. 14 and 16). In fact, these equations are identical with the exception that the *Q*_2_ term in the reward equations is replaced by the *O*_T_ term in the calibration equations. This suggests a strong conceptual link between these two optimization contexts, with *Q*_2_ and *O*_T_ performing closely related functions. For instance, the optimal type 2 criteria in a reward context with *Q*_2_ = 2 are identical to the optimal type 2 criteria in a calibration context with *O*_T_ = 2. This entails that for the ideal observer, rewarding the avoidance of high confidence errors twice as much as the accrual of high confidence corrects (*Q*_2_ = 2) is equivalent to calibrating confidence such that high confidence is reported whenever a response is at least twice as likely to be correct as it is to be incorrect (*O*_T_ = 2).

Due to the close similarity of their equations, *d’* has a similar impact on the type 2 criteria optimizing calibration to a type 1 accuracy threshold as it does for type 2 criteria optimizing type 2 reward. In the most relevant case where *p*(correct_1_)_T_ > 0.5, increasing *d’* makes the optimal type 2 criteria more liberal (Figure 6). This is intuitive in that as *d’* increases, the evidence distributions *f*(*x*|S1) and *f*(*x*|S2) become increasingly separated, which entails that the likelihood ratio needed to achieve a target level of accuracy occurs at less extreme values of the decision axis.

#### Optimizing the difference between type 2 hit rate and false alarm rate

A somewhat different approach from the foregoing is to maximize the difference between type 2 hit rate and false alarm rate (HR_2_ - FAR_2_). This strategy differs from the others in that HR_2_ and FAR_2_ do not depend upon stimulus priors *p*(S1) and *p*(S2), and as a consequence the optimal type 2 criteria for maximizing HR_2_ - FAR_2_ (${c}_{2,{``\mathrm{S}1"}}^{\mathrm{HF}^*}$ and ${c}_{2,{``\mathrm{S}2"}}^{\mathrm{HF}^*}$) do not depend on on the optimal type 1 criterion ${c}_{1}^{\mathrm{A}^*}$ (which acts as a kind of correction for unequal priors; see Eq. 2 and Figure 3). Rather, these optimal type 2 criteria depend only on type 1 hit rate and false alarm rate (Eqs. 22 and 23). Another distinct characteristic of this strategy is that for a fixed value of the type 1 criterion (any *c*_1_, optimal or not), the type 2 criteria for maximizing (HR_2_ - FAR_2_) are relatively independent of *d’*, unlike the optimal type 2 criteria for the other optimization contexts (compare Figure 7 to Figures 4, 5, 6).

In the special case where stimulus priors are equal (*p*(S1) = *p*(S2)) and the observer is unbiased (${c}_{1} ={c}_{1}^{\mathrm{A}^*}=0$), maximizing HR_2_ - FAR_2_ is equivalent to calibrating confidence to one’s average type 1 accuracy, i.e. setting *p*(correct_1_)_T_ = *p*(correct_1_) (see Section "Optimal type 2 criteria for maximizing HR_2_ - FAR_2_" for full reasoning).

#### The influence of suboptimal metacognition upon optimal type 2 criterion setting

The foregoing discussion applies to optimal type 2 criterion setting in the classical signal detection theory (SDT) model. However, SDT makes a strong prediction about the relationship between metacognitive sensitivity and task performance (i.e. that meta-*d’* = *d’*) which in practice does not always hold, necessitating consideration of alternative models.

We considered two simple models that allow for alteration in type 2 evidence samples, which causes suboptimal metacognitive sensitivity relative to SDT expectation (meta-*d’* < *d’*) via two distinct mechanisms (type 2 noise and type 2 signal loss). We examined the behavior of these models in two different optimization contexts (maximizing type 2 reward and HR_2_ - FAR_2_). Because exact derivation of optimal type 2 criteria in these models was not possible, we examined their behavior using computational simulations. In the simulations, we held the type 1 parameters of the models fixed and examined how changes in the parameters controlling metacognitive sensitivity affected optimal type 2 criterion setting.

In all cases, optimal type 2 criterion setting changed as a function of metacognitive sensitivity, confirming that this is a relevant factor to consider in analyzing type 2 criterion setting (Figure 8, expanded in Figures S3.1 and S3.2 of Supplementary Material S3). However, we also showed that decreasing metacognitive sensitivity could make optimal type 2 criteria become either more liberal or more conservative depending on the model and optimization context (Figures S3.1 and S3.2, panels A, B, D, and E). Moreover, the costs of neglecting to take suboptimal metacognitive sensitivity into account when setting type 2 criteria can be either small or considerable, depending on the model (Figures S3.1 and S3.2, panels **C** and **F**). Crucially, even when *M*_ratio_ is matched, different sources of metacognitive suboptimality can entail different rules for optimal type 2 criterion setting (Figure 9).

These results demonstrate that when analyzing optimal type 2 criterion setting outside of the confines of classical SDT, it is necessary to pay special attention to the unique details of the model and optimization context under consideration. We provide the code used to conduct the simulations presented in this manuscript in the opt_t2c toolbox (https://github.com/CNClaboratory/opt_t2c). This code can be readily adapted to investigate optimal type 2 criterion setting in different models, optimization contexts, and parameter settings than the ones considered here.

### Assessing the metacognitive strategies of real observers

#### General considerations

Thus far we have discussed normative strategies of type 2 criterion setting for ideal observers, but naturally this theoretical work is primarily of interest to provide context for better understanding the confidence reporting behavior of actual observers. Two questions of interest here are (1) what type 2 criterion setting strategy do observers spontaneously use when not given explicit instruction? And (2) how well are observers able to enact a given type 2 criterion setting strategy when explicitly instructed to do so?

Notably, current protocols used to study confidence often do not implement a *normative* set of instructions for confidence reports, instead letting participant interpret “high” and “low” confidence as they wish (De Martino et al., 2013; Fleming et al., 2010; Gherman & Philiastides, 2018; Zylberberg et al., 2012, 2014). In such cases, the observer may spontaneously attempt to follow a normative strategy, such as calibrating reports of high confidence to a threshold accuracy of 80% correct, or alternatively they may adopt a more heuristic approach that does not strictly follow any of the optimization strategies considered here. Even in cases where an observer attempts to follow a normative strategy – of their own volition, or to follow task instructions – they may not always be able to execute the strategy effectively or completely, necessitating a nuanced analysis.

As we have seen, type 2 criterion setting strategies involve deciding how to report confidence in order to pursue a definite objective (e.g. maximizing reward), making adjustments for various factors that mediate the relationship between criterion and outcome (e.g. *d’*, *c*_1_, *p*(S2), *Q*_2_, *O*_T_). It follows that fully understanding an observer’s type 2 criterion setting strategy requires measuring their confidence reports across conditions where factors that potentially modulate type 2 criterion setting (e.g. *d’*, *c*_1_, *p*(S2), and where applicable, *Q*_2_, *O*_T_, or other factors) are systematically manipulated through experimental manipulations. With such data in hand, the observer’s empirical pattern of type 2 criterion setting across conditions can then be compared with the theoretical patterns of type 2 criterion setting corresponding to one or more strategies for optimal type 2 criterion setting in order to better understand the observer’s criterion setting strategy.

The manner in which this comparison of empirical and theoretical type 2 criterion setting should be conducted depends in part on whether the observer’s strategy is instructed or spontaneous. When the observer is instructed to use a particular strategy (e.g. maximizing reward), their type 2 criterion setting patterns can be compared to the optimal type 2 criteria for that strategy to assess how effective the observer was at implementing the instructed strategy (Fleming & Dolan, 2010; Lebreton et al., 2018; Locke et al., 2020). In such cases, in addition to exploring the influences of *d’*, *c*_1_, and/or *p*(S2), the experimenter may wish to systematically vary the relative reward quotient *Q*_2_ or the threshold level of accuracy *O*_T_ (where applicable) in order to investigate how well the observer can adjust their type 2 criteria to various performance targets. Some studies have used this approach to estimate how incentivizing confidence reports influences metacognitive bias. Their findings suggest that human participants struggle to adopt a strategy that maximizes payoffs, due to the effect of loss aversion (Fleming & Dolan, 2010), difficulty in integrating reward with priors (Locke et al., 2020), or interactions between rewards and confidence reports themselves (Lebreton et al., 2018).

When the observer is free to choose their own strategy, analysis might more broadly investigate which optimal strategy (if any) most closely resembles the observer’s empirical type 2 criterion setting patterns across conditions. In the absence of explicit instruction regarding target outcomes or strategies, experimental manipulation of *Q*_2_ and *O*_T_ is presumably not applicable, and so experiments would instead focus on manipulating *d’*, *c*_1_, and/or *p*(S2). Indeed, investigating optimization of externally imposed rewards seems incompatible with investigating spontaneous type 2 criterion setting behavior, since the experimental introduction of a system of rewards and punishments already implies the imposition of norms for criterion setting. Conversely, analyzing the observer’s spontaneous criterion setting behavior in terms of the calibration framework would be a natural choice. In this case, given that *O*_T_ is not experimentally controlled, analysis could assess what value of *O*_T_ best explains the observer’s type 2 criterion setting patterns as other factors such as *d’* and *p*(S2) change. If the value of *O*_T_ inferred from the observer’s type 2 criteria across conditions appears roughly constant, this could justify an inference that the observer is employing a strategy to calibrate confidence ratings to accuracy. However, if *O*_T_ varies substantially across conditions, the situation is less clear: the observer may be attempting to calibrate confidence and not succeeding; they may be calibrating confidence by appropriately considering some factors (e.g. *d’*) but not others (e.g. *p*(S2)) -- see Factorizing the optimization strategies below; they may be pursuing another optimization objective entirely; or they may be using a heuristic approach that has no clear interpretation as an optimization strategy. If the observer indeed utilizes a strategy or heuristic that does not resemble any of the optimal strategies considered here, analysis could illuminate the nature of this strategy or heuristic by characterizing the observer’s type 2 criterion setting patterns across conditions.

Given the close link between the reward and calibration frameworks, the value of *O*_T_ that best explains an observer’s behavior could also be interpreted as reflecting a set of *internal* “rewards” associated with different type 2 outcomes, as summarized in an internal reward quotient *Q*_2_ with the same value as *O*_T_. These internal “rewards” could correspond to e.g. emotional states or cognitive appraisals of value that contribute to criterion setting. For instance, if an observer spontaneously sets *O*_T_ to a high value of 9 (only reporting high confidence for decisions at least 90% likely to be correct), this is equivalent to setting *Q*_2_ = 9 in a reward framework, which could reflect the spontaneously chosen strategy of a risk-averse observer who values avoiding high confidence errors nine times as strongly as accruing high confidence correct responses.

Optimizing type 2 accuracy differs qualitatively from the other optimization contexts considered here, which has consequences for experimental investigations of this strategy. As noted previously, optimizing type 2 accuracy amounts to using low confidence reports to flag likely errors, which is not consistent with how participants typically spontaneously choose to report confidence (e.g. Charles & Yeung, 2019). Thus, assessing an observer’s adherence to the strategy of optimizing type 2 accuracy may not be directly relevant for many research purposes, with the notable exception of research on error detection.

There is also a conceptual tension involved in instructing an observer to optimize type 2 accuracy. The ideal observer must compute the type 1 criterion that optimizes accuracy (${c}_{1}^{\mathrm{A}^*}$) in determining their type 2 criteria (Eqs. 6 and 8). But if the observer can compute ${c}_{1}^{\mathrm{A}^*}$ for the purposes of type 2 criterion setting, they should also be able to use it for type 1 criterion setting as well and set ${c}_{1} ={c}_{1}^{\mathrm{A}^*}$. In this case the strategy for optimizing type 2 accuracy is trivial – always report high confidence. However, if the observer has some rational grounds for setting ${c}_{1} \ne {c}_{1}^{\mathrm{A}^*}$ in spite of being *able* to compute ${c}_{1}^{\mathrm{A}^*}$, then it becomes meaningful to investigate whether the observer can optimize type 2 accuracy (i.e. accurately detect likely errors) in cases where their type 1 criterion setting strategy causes them to sometimes make decisions that are likely to be incorrect. For instance, suppose that the reward structure of the task incentivizes the observer to set a type 1 criterion that maximizes reward but not accuracy (i.e. ${c}_{1} ={c}_{1}^{\mathrm{R}^*}\ne {c}_{1}^{\mathrm{A}^*}$). Here it might be interesting to ask whether the observer can reliably report when this type 1 response strategy generates responses that are likely to be incorrect, which would require setting one type 2 criterion equal to the actual type 1 criterion ${c}_{1}^{\mathrm{R}^*}$, and the other equal to the type 1 criterion for optimizing accuracy, ${c}_{1}^{\mathrm{A}^*}$, in line with Eqs. 6 and 8.

In practice, resource limitations might often preclude an experimenter from exhaustively probing the influence of factors such as *d’*, *p*(S2), etc. on type 2 criterion setting. Comparison of empirical and theoretical type 2 criteria can always be conducted regardless – even, in principle, for data from a single experimental condition where no potential influences on type 2 criterion setting are systematically varied. The analysis results in such cases can still be informative, provided that the conclusions one draws from the analysis take appropriate stock of the limitations imposed by the available data.

One possible approach for investigating type 2 criterion setting in the face of such resource limitations would be to focus the experimental design and analysis on specific *aspects* of criterion setting strategy, such as the potential influence of *d’* on type 2 criterion setting, as discussed below.

#### Factorizing the optimization strategies

Thus far we have discussed optimal strategies as if they are unitary, undifferentiated processes, but it is also worth noting that the optimal type 2 criterion formulae can be decomposed into several mathematical components, each of which encompasses one particular facet of the optimal criterion setting strategy. Consideration of these separate components could provide further insights and discoveries regarding type 2 criterion setting. For instance, an observer might only partially implement an optimal type 2 criterion setting strategy by implementing some aspects of the strategy but not others.

In the case of optimizing type 2 reward and calibration, the formulae for the optimal type 2 criteria (Eqs. 14, 16, 19, and 20) suggest three separable components of the optimal criterion setting strategy: (1) the two response-specific type 2 criteria are set symmetrically around ${c}_{1}^{\mathrm{A}^*}$, which itself depends on *p*(S2) and *d’*; (2) type 2 criteria become more liberal (closer to ${c}_{1}^{\mathrm{A}^*}$) with increasing *d’* (provided *Q*_2_ > 1 or *O*_T_ > 1); and (3) type 2 criteria become more conservative (farther from ${c}_{1}^{\mathrm{A}^*}$) with increasing *Q*_2_ or *O*_T_. It could be the case that e.g. an observer neglects (1) entirely by failing to adjust type 2 criteria as a function of stimulus priors, but nonetheless accomplishes (2) and (3) and thereby partially follows the optimal strategy. Indeed, prior findings suggest the existence of similar dissociations when accounting for stimulus prior and rewards (Lebreton et al., 2018; Locke et al., 2020).

When optimizing HR_2_ - FAR_2_, the formulae for the optimal type 2 criteria (Eqs. 22 and 23) imply a different set of component strategies: (1) type 2 criteria shift as a function of *c*_1_ due to the effect that *c*_1_ has on HR_1_ and FAR_1_ (Figure 7); (2) type 2 criteria are largely independent of *d’*; and (3) type 2 criteria for “S1” and “S2” responses are always negative and positive, respectively, when *d’* > 0. (See Section "Optimal type 2 criteria for maximizing HR_2_ - FAR_2_" above for further discussion on these somewhat unusual properties.) Here again, it could be the case that an observer attempting to optimize HR_2_ - FAR_2_ might follow some but not all of these component strategies.

The procedure for optimizing type 2 accuracy is simpler than the other optimization contexts in that it only requires computation of the optimal type 1 criterion, ${c}_{1}^{\mathrm{A}^*}$ (Eqs. 6 and 8). The empirical test for this strategy would be to assess whether a participant sets their type 2 criteria as close as possible to ${c}_{1}^{\mathrm{A}^*}$, subject to consistency constraints imposed by the placement of their actual *c*_1_.

#### Assessing over-confidence and under-confidence

Whenever there is a normative standard for what an observer’s confidence *should* be, it follows that they can potentially be over-confident or under-confident relative to that standard (Fleming & Dolan, 2010; Lebreton et al., 2018). Specifically, the observer is over-confident if their actual type 2 criteria are more liberal than the optimal type 2 criteria, leading to more frequent high confidence reports than is optimal, and similarly they are under-confident if their actual type 2 criteria are more conservative than the optimal type 2 criteria, leading to less frequent high confidence reports than is optimal. Being able to rigorously characterize an observer’s confidence reports as over-confident or under-confident in this way can add an extra dimension of richness to understanding their metacognitive performance.

However, there may be cases where it is not trivial to decide which strategy the observer is attempting to follow, or ought to follow – especially when they do not receive explicit instruction on what strategy to use. In such cases where there are no clear grounds for saying what the observer’s confidence *should* be, it follows that there are no clear grounds for characterizing their performance as over- or under-confident. A somewhat related and important point is that the same confidence behavior can be over-confident relative to one optimization strategy, and under-confident relative to another. Just as there is not a single, monolithic optimal strategy for rating confidence, but rather different strategies that are optimal *relative to* different objectives, similarly there is no such thing as over- or under-confidence in the abstract, but rather there is over- or under-confidence *relative to* a given standard. Thus, some caution and nuance is required when assessing an observer’s putative over- or under-confidence.

One approach for quantifying over- or under-confidence in a standardized way is to normalize the distance of the actual type 2 criterion from the type 1 criterion by the distance of the optimal type 2 criterion from the type 1 criterion, i.e. (*c*_2_ - *c*_1_) / (c^*^_2_ - *c*_1_) (Charles et al., 2020). This ratio thus expresses type 2 criterion setting *relative* to the optimal standard, where over-confidence corresponds to the ratio being less than 1 (i.e., *c*_2_ is “too close” to *c*_1_), under-confidence corresponds to the ratio being greater than 1 (i.e., *c*_2_ is “too far” from *c*_1_), and optimality corresponds to the ratio being exactly 1. This approach has some affinity with the standardized expression of metacognitive sensitivity as the ratio of meta-*d’* to *d’* (Maniscalco & Lau, 2012).

#### Analyzing type 2 criterion setting when meta-*d’* ≠ *d’*

In most of the above, we considered evaluating an observer’s metacognitive strategy using the formulae for optimal type 2 criteria in classical signal detection theory, which assumes meta-*d’* = *d’*. However, it is often the case empirically that meta-*d’* ≠ *d’*. In Section "The impact of suboptimal metacognition: when meta-*d’* < *d’*" we showed that in such cases an alternative model is needed to specify the computational processes governing the observer’s metacognitive sensitivity, and optimal type 2 criterion setting according to this alternative model may differ from its classical SDT counterpart. How should assessment of the observer’s type 2 criterion setting proceed in such cases?

The most thorough approach would be to consider the observer’s data in light of a model that specifies computational processes which account for deviations from the metacognitive sensitivity expected by classical SDT, analogous to the simple type 2 noise or type 2 signal loss models considered in this paper. Ideally when applied to empirical data, the model’s appropriateness for characterizing the data would be well-validated e.g. by model comparison analysis or by reference to previous research. This model could then be used to ascertain the optimal type 2 criteria, either by derived mathematical formulae or by simulations. The optimal type 2 criteria obtained from the model could then be compared to the observer’s actual type 2 criteria according to fits of the model to the observer’s data, in line with the considerations discussed above (Sections "General considerations", "Factorizing the optimization strategies", and "Assessing over-confidence and under-confidence").

Depending on the research question, the optimal type 2 criteria derived from classical SDT might still be of interest for shedding light on empirical data even in cases where meta-*d’* ≠ *d’*. For instance, it might be of interest to know how the observer would ideally set type 2 criteria if their metacognitive sensitivity were also ideal according to SDT, i.e. if it had been the case that the observer’s meta-*d’* were equal to their empirically established *d’*. It might also be of interest to know how the ideal observer would set their type 2 criteria if they ignored deviations of metacognitive sensitivity from SDT-defined optimality, or simply had no introspective access to such deviations to begin with. This question might be of particular interest in cases where the deviation of meta-*d’* from *d’* is small, and/or cases where neglecting sources of type 2 suboptimality has relatively small consequences for optimization outcomes (e.g. as in the type 2 noise model’s behavior described in Supplementary Material S3 and shown in Figure S3.1C and S3.1F).

Complications arising from meta-*d’* ≠ *d’* might also be relaxed in cases where the researcher is more interested in investigating qualitative patterns in type 2 criterion setting, rather than in computing exact type 2 criterion values. For instance, it is likely the case that for most plausible models allowing for meta-*d’* ≠ *d’*, the qualitative patterns that type 2 criteria should become more liberal with increasing *d’*, or more conservative with increasing *Q*_2_ or *O*_T_, hold. (Indeed, simulations confirm that these qualitative patterns hold under type 2 noise. We do not show these data here, but provide code for running this analysis in the opt_t2c toolbox.) If the researcher is mainly interested in assessing such qualitative patterns, then it may not be necessary to engage with the complexities of characterizing exact type 2 criterion values when meta-*d’* ≠ *d’*.

#### Alternative optimization strategies and heuristics

As we have alluded to in the preceding discussion, it is important to keep in mind that there are many possible type 2 criterion setting strategies that an observer could adopt aside from the four main optimal strategies considered in this paper. One category of such alternative strategies might be partial adoption of an optimal strategy, as considered in Section "Factorizing the optimization strategies"; for instance, an observer might neglect to take stimulus priors into account when enacting a calibration strategy due to ignorance or resource limitations, while still adhering to the other components of the optimal strategy (Locke et al., 2020). It is also possible that an observer might choose a strategy that is optimal for some objective not considered in this paper; for example, one natural choice might be to make reports of high confidence maximally diagnostic of accuracy by maximizing *p*(correct|high confidence) - *p*(incorrect|high confidence) – a measure related to, but distinct from, HR_2_ - FAR_2_. There are of course many other possible optimization contexts not discussed here, and exploration of them all is beyond the scope of the present project. Naturally, criteria that are optimal in one context are likely to be suboptimal in another context, so it is important to always interpret optimality *relative to* a given objective rather than characterize optimality in an absolute sense.

Finally, an observer could employ a more heuristic strategy that does not optimize for any particular objective, but nonetheless serves some useful purpose (for example, related to mutual information (Dayan, 2022) or maximum entropy (Bang et al., 2017)); this could even include an observer choosing to employ a strategy that combines aspects of two or more of the strategies discussed here, or combines one or more of these strategies with a completely separate strategy. The present work is only an initial exploration of the rigorous study of optimal metacognitive decision strategies in an SDT framework, and will need to be built upon by subsequent theoretical and empirical research which further expands on the space of optimal and actual metacognitive decision strategies.

#### Sample size and statistical inference

In this work we have focused on the theoretical foundations of analyzing type 2 criterion setting. It will be important for future work to further characterize the practical influence of sample size on applying the theory in order to make statistically sound inferences from real data (Barrett et al., 2013; Rahnev, 2023). Of particular interest here is the number of trials available for estimating parameters of the SDT model within a single condition for a single subject. Such future work could address questions such as: How does the precision and bias of parameter recovery from simulated data depend on the number of trials across different parameter values? What experimental design choices maximize the decisiveness of planned hypothesis tests or model comparisons while minimizing the number of trials needed (Rong & Peters, 2023)? How do actual and estimated type 2 sensitivity factor into these considerations? While such important questions are beyond the scope of the present work, we provide the tools to address such questions in the opt_t2c toolbox (https://github.com/CNClaboratory/opt_t2c).

### Advantages and disadvantages vary by strategy

Which type 2 criterion setting strategy an observer chooses to employ might depend on the relative advantages and disadvantages of the various strategies. Here we consider what those advantages and disadvantages might be. Since optimizing type 2 accuracy is limited to special cases where the observer is concerned with error detection, and since optimizing reward behaves similarly to calibration, we focus our discussion particularly on the comparison between optimizing type 2 calibration and maximizing HR_2_ - FAR_2_.

Calibration tethers confidence reports to a fixed level of accuracy regardless of changes to the decision-making context, which makes such confidence reports clear, immediately applicable to managing uncertainty and risk and useful for informing subsequent decisions (Balsdon et al., 2020; Boldt et al., 2019; Boldt & Gilbert, 2019; Guggenmos et al., 2016; Stolyarova et al., 2019), and readily communicable to other observers in a social context and hence useful for group decision-making (Bahrami et al., 2010, 2012; Bang et al., 2017; Koriat, 2012; Pescetelli et al., 2016). By comparison, the objective to maximize HR_2_ - FAR_2_ may be relatively obscure and unintuitive to typical human and animal observers since it requires assessing the probability of high confidence conditional on correct and incorrect responses, and then computing their difference.

On the other hand, the complexity of implementing the calibration strategy is a potential disadvantage. Calibration requires (1) accurately tracking stimulus priors (*p*(S2)) and task performance (*d’*), and (2) utilizing these values in the proper way to compute the correct type 2 criteria (Eqs. 19 and 20). These requirements are computationally demanding, and if the resource costs incurred by them are salient enough, it may actually be preferable for the observer to forgo attempting to implement the optimal calibration strategy and instead resort to simpler heuristics that approximate the optimal strategy well enough while incurring significantly less computational costs (Adler & Ma, 2018; Jones, 1999; Schwartz et al., 2011). An additional difficulty arises from the fact that both *p*(S2) and *d’* are aggregate measures that characterize aspects of the decision-making context that only fully manifest over extended time periods or many trials, and so the observer may encounter further difficulties in accurately updating type 2 criteria to account for changes to the decision-making context. Indeed, preliminary evidence suggests that whereas type 2 criteria can adjust to changes in the decision-making context when these changes are grouped into temporally contiguous blocks of trials, such adjustment does not take place when the changes occur on faster time scales at the level of individual trials (Maniscalco et al., 2024).

By comparison, the strategy of maximizing HR_2_ - FAR_2_ is potentially far simpler and less resource-intensive to implement in that it does not depend on stimulus priors and is largely independent of changes in *d’*; rather, it mainly exhibits a relatively simple dependence on the observer’s type 1 criterion (Figure 7). Furthermore, we have shown that when stimulus priors are equal and the observer’s type 1 criterion is unbiased (${c}_{1} ={c}_{1}^{\mathrm{A}^*}$), optimizing HR_2_ - FAR_2_ is equivalent to calibrating confidence to one’s average type 1 accuracy, suggesting that this strategy could function as a kind of convenient heuristic for approximating a reasonable and robust calibration strategy. Some preliminary evidence (Charles et al., 2020) suggests that participants might indeed employ a type 2 criterion setting strategy that aims to maximize HR_2_ - FAR_2_. In that study, participants reported their confidence on a 4-point scale; results demonstrated that participants placed the type 2 criteria separating ratings 2 and 3 (corresponding to the middle of the confidence scale) close to the location of the type 2 criteria that maximize HR_2_ - FAR_2_. Further evidence is needed to confirm if optimizing HR_2_ - FAR_2_ is indeed a strategy widely employed by human observers.

### Application to common experimental practices

#### Optimal type 2 criterion setting for confidence rating scales with more than two levels

We have discussed optimal type 2 criterion setting when utilizing a binary confidence rating scale consisting of low vs high confidence. This entails setting a single type 2 criterion on either side of the type 1 criterion which separates low vs high confidence regions of the decision axis for each response type. However, many studies use more expansive confidence rating scales; common examples include 4-point scales (e.g. Charles et al., 2020), 6-point scales (e.g. Boldt & Yeung, 2015) and semi-continuous scales (e.g. Rahnev & Fleming, 2019; Rouault et al., 2019). In general, for an *N*-point confidence rating scale, *N*-1 type 2 criteria for each response type are required to divide the decision axis on either side of the type 1 criterion into *N* regions corresponding to the *N* confidence levels. Thus, the question naturally arises: how does the present treatment of optimal type 2 criteria for binary confidence ratings apply to more expansive rating scales requiring more than one type 2 criterion per response type?

A preliminary general observation is that although for many purposes it is convenient to reduce confidence rating to a binary low vs high judgment, such an approach does not necessarily preclude application to scales with more than two levels. For instance, type 2 ROC curves (Clarke et al., 1959; Galvin et al., 2003; Maniscalco & Lau, 2014) are an example of an analysis approach that both binarizes confidence ratings and also allows for consideration of the entire rating scale. This is accomplished by computing type 2 hit rate and false alarm rate for every possible binarization of the scale. For instance, a 4-point rating scale has three possible ways of being reduced to a 2-point scale, and each such binarization generates a corresponding (FAR_2_, HR_2_) pair for use in type 2 ROC analysis.

However, most of the optimization contexts considered here involve finding *the* single, unique type 2 criterion for each response type that yields *the* single best outcome for the observer’s objective. For instance, there is only one type 2 criterion (for a given response type) that yields the maximum possible type 2 accuracy, type 2 reward, or HR_2_ - FAR_2_. Thus, the criteria that optimize these outcomes can only apply to one of the multiple type 2 criteria for a given response type when more than two levels of confidence can be reported.

Since optimizing type 2 accuracy entails reporting low confidence only when the response is likely to be an error, the corresponding optimal type 2 criteria may not be relevant for rating scales in which the lowest possible confidence rating is intended to denote responses that have minimal supporting evidence, as opposed to responses that are not merely uncertain but are actually suspected as likely to be incorrect. However, some confidence rating scales explicitly include options to indicate that the response was likely incorrect (e.g., (Boldt & Yeung, 2015)); for such scales, the optimal type 2 criteria for maximizing type 2 accuracy provide a natural characterization of what value should be used for the type 2 criteria separating “likely incorrect” from “likely correct” ratings.

When considering tasks in which confidence is reported on several levels, the type 2 criterion optimizing HR_2_ - FAR_2_ for a given response type could be an appropriate target for the middle of the scale as it occurs at intermediate values on the decision axis (Figure 7). This is consistent with the previously mentioned finding that in one data set, participants set the type 2 criteria separating confidence ratings of 2 and 3 at a value consistent with the criterion that maximizes HR_2_ - FAR_2_ (Charles et al., 2020). Similarly, a reward matrix that is contingent upon type 2 outcomes as outlined in Table 4 implies a single type 2 criterion for each response type that yields maximum reward. It is possible that an expanded reward matrix that includes different reward outcomes for each level of an *N*-point rating scale for *N* > 2, contingent on the accuracy of the type 1 response, could specify normative values for each of the *N*-1 type 2 criteria and thereby be applicable to *N*-point rating scales. We leave investigation of this possibility to future work.

The notable exception here is the calibration framework, which naturally extends to arbitrarily many type 2 criteria and thus to arbitrarily large rating scales. This is because it is natural to evaluate confidence with reference to many threshold levels of accuracy, none of which are mutually exclusive with the others. For instance, to utilize a 4-point rating scale, one might set three type 2 criteria corresponding to accuracy thresholds of 70%, 80%, and 90% correct. There is only one optimal type 2 criterion for each threshold level of accuracy, but arbitrarily many such thresholds can be evaluated simultaneously.

#### Using average confidence as a proxy for type 2 criterion setting

It is common practice for researchers to analyze average confidence ratings, and these are sometimes taken as a kind of proxy for measuring type 2 criterion setting or metacognitive bias per se. However, caution is needed in making inferences about an observer’s metacognitive decision strategy from average confidence, since according to SDT confidence depends on *d’* and *c*_1_ as well as the observer’s type 2 criteria, and only the latter pertain directly to the observer’s metacognitive decision strategy per se.

The influence of *d’* upon average confidence is easy to understand. Holding type 1 and type 2 criteria constant, increasing *d’* has the effect of increasing the separation of the evidence distributions for S1 and S2 stimuli, with the result that a higher proportion of the distributions’ probability mass exceeds the type 2 criteria and therefore higher levels of confidence are more frequently reported. Thus, it is possible for changes in confidence to be driven entirely by changes in *d’*, even as an observer’s type 2 criteria remain fixed. As a consequence, it is not appropriate to use average confidence to assess the influence of *d’* upon metacognitive decision strategy, but rather a modeling approach must be used to isolate type 2 criterion setting independent of changes in *d’*. For other purposes it may still be appropriate to use average confidence as a proxy for type 2 criterion setting, but only if *d’* is held constant across conditions.

The effect of *c*_1_ upon average confidence is more subtle. Changes in *c*_1_ can alter average confidence if the observer employs a strategy that adjusts type 2 criteria for “S1” and “S2” responses relative to *c*_1_. For instance, suppose an observer adopts a simple strategy of always setting *c*_2,“S1”_ = *c*_1_ - 1 and *c*_2,“S2”_ = *c*_1_ + 1. Suppose in condition A, *d’* = 2 and *c*_1_ = 0; then *c*_2,“S1”_ = - 1, *c*_2,“S2”_ = 1, and the observer’s average *p*(high confidence) = 0.52. Suppose in condition B, *d’* remains constant but *c*_1_ shifts to a new value of 1; then *c*_2,“S1”_ = 0,* c*_2,“S2”_ = 2, and the observer’s average *p*(high confidence) = 0.58. The rightward shift of the decision criteria causes an increase in average confidence for “S1” responses (*p*(high confidence) = 0.68) and a decrease in average confidence for “S2” responses (*p*(high confidence) = 0.31), while also making “S1” responses more frequent overall (*p*(“S1” response) = 0.74), with the net effect that overall confidence increases. When *c*_1_ is biased (*c*_1_ ≠ 0), *p*(S2) can also influence response-specific and overall average confidence. Thus, it is recommended to keep not only *d’* but also *c*_1_ and *p*(S2) constant across conditions if using average confidence to make inferences about metacognitive decision strategy.

To summarize, because average confidence depends on a range of factors in addition to type 2 criterion setting, it follows that average confidence is only appropriate for making inferences about metacognitive decision strategy (i.e. type 2 criterion setting) when other factors are properly controlled. This entails ensuring that *d’*, *c*_1_, and *p*(S2) are held constant across conditions where average confidence is compared. But this constraint requires some experimental finesse to achieve in real data, and furthermore restricts one from investigating the potential role of *d’*, *c*_1_, and *p*(S2) in an observer’s type 2 criterion setting strategy. Thus, for many purposes it may be preferable to use a modeling approach to make inferences about metacognitive decision strategies by reference to type 2 criterion setting, rather than using average confidence. This is not to say that average confidence is not an interesting or relevant measure in its own right, only that it is not an ideal tool for making specific inferences about metacognitive decision strategy per se.

### Closing remarks

Here, we have used type 2 signal detection theory to derive optimal metacognitive criterion setting strategies under four optimization contexts: type 2 accuracy, type 2 reward, calibration of confidence to accuracy, and maximizing the difference between type 2 hit rate and false alarm rate. Our formal derivation approach provides both theoretical and practical contribution to the study of decision-making and metacognition. Further, our simulation code provides a practical tool by which researchers in the field may explore the impact of metacognitive suboptimality on confidence judgments across a wide range of situations and tasks. Together, our findings represent a significant step forward in the scientific study of metacognitive evaluation of decisions made under uncertainty.

## Supplementary information

Below is the link to the electronic supplementary material.

Supplementary file1 (PDF 311 KB)

Supplementary file2 (PDF 341 KB)

Supplementary file3 (PDF 693 KB)

## Footnotes

1 Throughout the manuscript we use “type 2” and “metacognitive” interchangeably. To prevent possible confusion, note that we never use the terms “type 1” and “type 2” in this paper to refer to errors of commission (false alarms, sometimes called “type I errors”) or errors of omission (misses, sometimes called “type II errors”).

2 In most cases, we use the term “ideal” to refer to optimal criterion setting relative to a given objective (e.g. to maximize accuracy or reward). When we discuss the ideal or optimal type 1 criterion, we are usually referring to the type 1 criterion that maximizes type 1 accuracy, unless context specifies otherwise. When discussing ideal type 2 criterion setting, we allow for the possibility that the type 1 criterion is not optimal. Later, when discussing type 2 sensitivity, we sometimes use the term “ideal” or “SDT-ideal” to refer to task performance where meta-*d'* = *d'*. In all cases, the exact meaning of terms like “ideal” and “optimal” should be clear from context and notation (Table 2).

3 Throughout this manuscript, we write “S1” or “S2” (in quotes) to indicate an observer’s subjective report that they judge the stimulus to belong to the S1 or S2 category, as contrasted to writing S1 or S2 (without quotes) to indicate the objective identity of the stimulus. This follows existing convention e.g. as in Maniscalco & Lau 2014.

4 The standard assumption of equal variance across the S1 and S2 distributions is typically appropriate for discrimination tasks and two-interval forced-choice detection tasks, but not for yes-no detection tasks. Therefore, we set the variances of S1 and S2 to be equal here, with the understanding that this assumption may not be appropriate for yes-no detection tasks (Green & Swets, 1966; Kellij et al., 2020; Macmillan & Creelman, 2004; Mazor et al., 2020, 2021).

5 It is possible to define type 2 rewards differently for “S1” and “S2” responses, which might make sense in some experimental designs or situations. However, here for simplicity we only consider the case where the same type 2 rewards are available regardless of the type 1 response category. It is trivial to extend this analysis to different response-specific type 2 payoff matrices by substituting response-specific reward quotients Q_2,”S2”_ and Q_2,”S1”_ into Eq. 14 and 16 respectively.

6 The presence of Gaussian type 2 noise entails that the integrals corresponding to p(high confidence) terms in the SDT model contain the normal cumulative distribution function, which has no closed form expression. It follows that in the presence of Gaussian type 2 noise, there is no closed form solution for p(high confidence) terms.

7 When *σ*_2_ = 0 and 0 < *k* < 1, (1 - *k*) scales the mean and standard deviation of *x*_1_ to an equal extent, and so signal-to-noise ratio for the scaled *x*_2_ samples is equivalent to the signal-to-noise ratio of the original *x*_1_ samples. As a consequence, meta-*d’* remains equal to *d’* for all *k* in 0 < *k* < 1 when *σ*_2_ = 0.
