# Supplementary material for: Optimal metacognitive decision strategies in signal detection theory
Source: Psychon Bull Rev. 2024 Nov 18;32(3):1041–69. doi: 10.3758/s13423-024-02510-7 (PMC12092500; doi:10.3758/s13423-024-02510-7)
Supplement: Supplementary file 1 — Supplementary file1 (PDF 311 KB) [file 13423_2024_2510_MOESM1_ESM.pdf]

Optimal Metacognitive Decision Strategies in Signal Detection Theory

Brian Maniscalco*, Lucie Charles*, & Megan A. K. Peters

Supplementary Material S1

A Brief Primer on Type 1 and Type 2 SDT

Here we provide a brief primer on signal detection theory (SDT) as applied to type 1 and type 2 tasks. The main manuscript presupposes familiarity with this material.

1. Type 1 SDT

In its simplest and most canonical form, SDT characterizes the task of using ambiguous evidence to make a binary classification decision about some external state of the world. The observer is modeled as making this decision by using a continuous, one-dimensional internal evidence variable x. This one-dimensional continuum forms the *decision axis* along which evidence is evaluated. On each trial, x takes on a single numerical value; the exact value it takes depends on both the strength of evidence (e.g. due to stimulus strength, attentional state, etc.) and stochastic processes, such that identical presentations of a stimulus on successive trials may generate different evidence values x due to random noise. SDT assumes that the distribution of evidence values generated by repeated presentations of a stimulus across trials is Gaussian, where the mean of the distribution reflects the average strength of internal evidence generated by the stimulus. Thus, in the case where one of two possible stimuli, S1 or S2, is shown on each trial, there are two Gaussian distributions, $f(x|\text{S1})$ and $f(x|\text{S2})$, corresponding to the probability density functions of evidence values contingent on presentation of S1 or S2 (**Figure 1A**, main text). By convention, the mean of the S1 distribution is less than the mean of the S2 distribution, and is often set such that the mean of S1 is the negative of the mean of S2. For simplicity, in this paper we make a standard assumption that the standard deviations of the two distributions are equal[1]. In the following primer, we also make the additional simplifying assumption that S1 and S2 are equally likely to occur, i.e. the prior probabilities of S1 and S2 are equal unless otherwise specified. This assumption is relaxed as we discuss deriving optimal criterion placement for the type 1 and type 2 criteria (and their relationship) in the main manuscript.

[1] The standard assumption of equal variance across the S1 and S2 distributions is typically appropriate for discrimination tasks and two-interval forced-choice detection tasks, but not for yes-no detection tasks. Therefore, we set the variances of S1 and S2 to be equal here, with the understanding that this assumption may not be appropriate for yes-no detection tasks (Green & Swets, 1966; Kellij et al., 2020; Macmillan & Creelman, 2004; Mazor et al., 2020, 2021).

Sensitivity – i.e., the observer's overall ability to distinguish between S1 and S2 – depends on the degree of overlap between the evidence distributions for S1 and S2. In cases where the distributions overlap substantially, the two stimuli generate very similar distributions of evidence, meaning that a given evidence value observed on a particular trial might be highly consistent with the presentation of either S1 or S2. Since the observer only ever has access to the evidence value generated by the stimulus on that trial and must use this to infer which stimulus was presented, such ambiguity makes the task difficult and entails the inevitability of frequent errors. By contrast, when the distributions are well-separated, then the stimuli are more easily distinguished and errors are rare. Sensitivity is quantified in SDT by the measure *d'*, which corresponds to the number of standard deviations that separate the means of the S1 and S2 distributions. When $d' = 0$, the distributions overlap perfectly and the observer's performance is at the chance level of 50% correct, whereas *d'* values of 1, 2, and 3 correspond to accuracy rates of 69%, 84%, and 93% when the observer's responding is unbiased (see below).

It is not enough to have a graded evidence value x on a given trial; the observer must use x to make a definite decision about whether the stimulus was S1 or S2. According to SDT, the observer accomplishes this by setting a decision criterion (denoted by the variable c_1) at some point along the decision axis, and comparing x to c_1 in order to decide what the stimulus was on the current trial. Specifically, the observer reports "S1" whenever $x < c_1$, and reports "S2" otherwise[2]. By convention, when the variances of the distributions are assumed to be equal, then $x = 0$ corresponds to the point on the decision axis where the distributions intersect, i.e. $x = 0$ is the evidence value for which S1 and S2 were equally likely to have generated x. Thus, an observer is said to have an unbiased criterion when $c_1 = 0$, a liberal criterion when $c_1 < 0$, and a conservative criterion when $c_1 > 0$ (where "liberal" and "conservative" connote the observer's propensity for reporting "S2").

An alternative formulation for the decision criterion c_1 is as the *likelihood ratio* of the stimulus distributions $\frac{f(x|\text{S2})}{f(x|\text{S1})}$ occurring at the location of the criterion (i.e. the likelihood ratio at $x = c_1$). This likelihood ratio β_1 is given by $\beta_1 = \frac{f(c_1|\text{S2})}{f(c_1|\text{S1})} = e^{c_1 d'}$. Thus, for an unbiased observer with $c_1 = 0$, the criterion can be expressed instead as $\beta_1 = 1$, i.e. for an unbiased observer the criterion is placed at the point on the decision axis where x is equally likely to have been generated by S1 and S2. Similarly, $\beta_1 < 1$ for a liberal criterion and $\beta_1 > 1$ for a conservative criterion. Expressing criterion in terms of β_1 can be conceptually useful, as seen in **Section 2.1.1** of the main manuscript. See **Supplementary Material S2**, **Section 5** for further discussion on β.

Together, *d'* and c_1 determine the observer's hit rate and false alarm rate, where hit rate = HR = p(response = "S2" | stimulus = S2) and false alarm rate = FAR = p(response = "S2" | stimulus = S1). HR and FAR correspond to the areas under the $f(x|\text{S2})$ and $f(x|\text{S1})$ curves exceeding c_1, respectively. Indeed, we infer an observer's *d'* and c_1 based on their empirically measured HR and FAR in a task using the equations $d' =$

[2] This notation follows the convention of writing "S1" (in quotes) to indicate an observer's subjective report that they judge the stimulus to belong to the S1 category, as contrasted to writing S1 (without quotes) to denote the objective identity of the stimulus as belonging to the S1 category.

$z(\mathrm{HR}) - z(\mathrm{FAR})$ and $c_1 = -0.5[z(\mathrm{HR}) + z(\mathrm{FAR})]$, where $z(\cdot)$ is the inverse cumulative distribution function for the Gaussian distribution.

Whereas d' is constrained by properties of the stimulus, environment, and observer, c_1 is free to vary depending on the observer's decision-making strategy or bias. Thus, it is possible that the same observer discriminating the same stimuli may set different values of c_1 in different experimental conditions that encourage or reveal different criterion setting strategies, even while d' remains fixed. In this case, each experimental condition will generate a distinct pair of HR and FAR values. Plotting the HR vs FAR values against each other yields a receiver operating characteristic (ROC) curve, which reveals how HR and FAR trade off as a function of criterion setting while sensitivity is held constant (**Figure 1B**, main text). Mathematically, the ROC curve corresponds to the infinite set of (FAR, HR) pairs that are generated for a fixed level of d' by sweeping c_1 from negative to positive infinity. The strong empirical success of SDT can essentially be framed as the remarkable ability of this very simple model to generate theoretical ROC curves that capture the forms taken by empirical ROC curves generated by human and animal observers over a wide range of tasks and circumstances (Green & Swets, 1966; Macmillan & Creelman, 2004).

2. Type 2 SDT

Thus far we have presented the SDT model of type 1 decision making, i.e. making judgments about states of the world. It is possible to extend the SDT model to type 2 decision making, i.e. making judgments about the accuracy of one's own decision (type 1 judgments), in a fairly straightforward way. For simplicity, here we will assume that the type 2 decision amounts to deciding whether to report "high confidence" or "low confidence" in the type 1 decision[3]. We start with a simple scenario in which we suppose that the observer sets two type 2 decision criteria on the decision axis, one on either side of the type 1 criterion c_1. These correspond to the criteria used to rate confidence separately for "S1" and "S2" responses, and so we call them $c_{2,\text{"S1"}}$ and $c_{2,\text{"S2"}}$.

Each type 2 criterion applies only to one kind of type 1 response, and so the value of the type 2 criterion is constrained to be located on the appropriate side of the type 1 criterion c_1. For example, since $c_{2,\text{"S1"}}$ is the criterion used to evaluate confidence for "S1" responses, and since "S1" responses by definition only occur for $x < c_1$, then it must be the case that $c_{2,\text{"S1"}} \leq c_1$. By similar reasoning, $c_{2,\text{"S2"}} \geq c_1$.

Evidence values further away from c_1 denote stronger magnitudes of evidence supporting one response or another, and so the observer reports high confidence for "S1" responses whenever $x < c_{2,\text{"S1"}}$, and similarly, high confidence for "S2" responses whenever $x > c_{2,\text{"S2"}}$.

Sensitivity in the type 2 task corresponds to how well the observer's type 2 responses distinguish between their own correct and incorrect type 1 responses. By analogy to the type 1 case, this type 2 sensitivity depends on how much overlap there is between the type 2 distributions of evidence, i.e. $f(x|\text{correct})$ and

[3] It is worth noting that the type 2 report could also be about something other than confidence, such as subjective clarity of the stimulus. It is also frequently assumed that the type 2 response scale could take on multiple ordinal categories (e.g., response on a scale of 1-4) or even continuous reporting (e.g., an analog slider for type 2 report). In such cases, however, it is always possible to reduce the confidence data into a binary high vs low categorization, e.g. by applying a median split.

$f(x|\text{incorrect})$. However, we cannot simply assume that these type 2 distributions are Gaussian, as we did in the type 1 case. In fact, specifying d' and c_1 in the type 1 SDT model already determines an expected set of type 2 distributions, and these are indeed *not* Gaussian (Galvin et al., 2003). For instance, for simplicity let us consider "S2" responses only, i.e. the portion of the decision axis where $x > c_1$ (**Figure 1C**, main text). Over this region of the decision axis, by definition, the observer responds correctly whenever the true stimulus is S2, and incorrectly when it is S1. Thus, for $x > c_1$, the $f(x|S2)$ and $f(x|S1)$ distributions correspond to correct and incorrect responses, respectively[4]. It follows that the degree of overlap between the $f(x|S2)$ and $f(x|S1)$ distributions over the $x > c_1$ region of the decision axis determines type 2 sensitivity for "S2" responses. Similar reasoning applies to the separate case of "S1" responses. For this reason, the SDT model predicts that type 1 d' and c_1 jointly determine type 2 sensitivity (Galvin et al., 2003; Maniscalco & Lau, 2012, 2014).

The $c_{2,\text{"S2"}}$ criterion determines which x values for "S2" responses are sufficient to generate "high confidence" responses. By analogy with the type 1 case, when the type 2 criterion is applied to the type 2 distributions, it generates type 2 hit rates and false alarm rates, where type 2 hit rate = HR_2 = p(high confidence | correct) and type 2 false alarm rate = FAR_2 = p(high confidence | incorrect). Following the notation for $c_{2,\text{"S2"}}$, we can write $HR_{2,\text{"S2"}}$ and $FAR_{2,\text{"S2"}}$ to denote type 2 hit rate and false alarm rates for "S2" responses. $HR_{2,\text{"S2"}}$ corresponds to the area under the $f(x|S2)$ curve that exceeds $c_{2,\text{"S2"}}$ (i.e. the probability of a high confidence "S2" response for the S2 stimulus) divided by the area under the $f(x|S2)$ curve that exceeds c_1 (i.e. the overall probability of an "S2" response for the S2 stimulus). Similarly, $FAR_{2,\text{"S2"}}$ corresponds to the area under the $f(x|S1)$ curve that exceeds $c_{2,\text{"S2"}}$ divided by the area under the $f(x|S1)$ curve that exceeds c_1. Similar reasoning applies to the separate case of "S1" responses.

For an observer with fixed type 1 and type 2 sensitivity observing a fixed set of stimuli, multiple ($HR_{2,\text{"S2"}}$, $FAR_{2,\text{"S2"}}$) pairs can be derived by adjusting the type 2 criterion across experimental conditions[5]. These can be plotted against each other to form an empirical type 2 ROC curve for "S2" responses (**Figure 1D**, main text), which describes how $HR_{2,\text{"S2"}}$ and $FAR_{2,\text{"S2"}}$ trade off as a function of type 2 criterion setting for a fixed level of type 2 sensitivity. Similar reasoning applies to "S1" responses.

As discussed above, SDT predicts that type 2 evidence distributions, and thus type 2 ROC curves, are jointly determined by type 1 d' and c_1. However, it is empirically observed that type 2 sensitivity can vary independently from type 1 sensitivity (Fleming et al., 2010), and so the simple SDT model cannot be the whole story about type 2 decision making. Nonetheless, it is theoretically useful to characterize observed type 2 sensitivity in terms of the value of d' that best characterizes a set of empirical type 2 ROC curve data, according to SDT. This is what is accomplished by the measure of type 2 sensitivity called meta-d' (Maniscalco & Lau, 2012, 2014). This measure allows us to estimate the metacognitive efficiency of an observer by comparing the meta-d' fitted to their type 2 ROC curves to their empirically measured d'. For

[4] Note that the distributions must be renormalized before they are proper probability density functions such that the total area under each curve sums to 1.

[5] It is also possible to create type 2 ROC curves within a single experiment or condition by having the type 2 rating scale offer more than two levels, as briefly mentioned above. Although many investigations use this method, the optimal setting of more than one type 2 criterion is beyond the scope of this paper. See **Section 3.4.1** of the main manuscript for further discussion.

an observer who is ideal according to SDT, it should be the case that meta-$d' = d'$, i.e. empirical type 2 ROC curves should be consistent with the theoretical type 2 ROC curves derived from SDT, as predicted from the observer's d' and c_1. If meta-$d' \neq d'$, this implies that the relationship between type 1 and type 2 sensitivity does not conform to SDT expectation. In most such cases meta-d' is smaller than d', which indicates suboptimal metacognitive sensitivity relative to SDT expectation. Alternatively, it has also been found that meta-d' can be greater than d' (Charles et al., 2013) if time-pressure on the response is increased and participants are able to detect and correct a large proportion of their fast erroneous guesses.

One interpretation of empirical findings of suboptimal metacognitive efficiency relative to SDT expectation (meta-$d' < d'$) is that the evidence used to form type 2 judgments is somehow degraded relative to the evidence used to form type 1 judgments. The details of such an account is still a matter of ongoing research (Barrett et al., 2013; Fleming & Daw, 2017; Fleming & Lau, 2014). One possibility is that additional noise corrupts type 2 distributions (Maniscalco & Lau, 2016; Peters et al., 2017; Shekhar & Rahnev, 2021a, 2021b), possibly because type 2 decisions occur later in time than type 1 decisions or are constructed in downstream brain regions (Maniscalco & Lau, 2016; Pleskac & Busemeyer, 2010). In **Section 2.5** of the main manuscript, we discuss how cases where meta-$d' < d'$ impact our findings.

References

Barrett, A. B., Dienes, Z., & Seth, A. K. (2013). Measures of metacognition on signal-detection theoretic models. *Psychological Methods*, *18*(4), 535–552.

Charles, L., Van Opstal, F., Marti, S., & Dehaene, S. (2013). Distinct brain mechanisms for conscious versus subliminal error detection. *NeuroImage*, *73*, 80–94.

Fleming, S. M., & Daw, N. D. (2017). Self-evaluation of decision-making: A general Bayesian framework for metacognitive computation. *Psychological Review*, *124*(1), 91–114.

Fleming, S. M., & Lau, H. (2014). How to measure metacognition. *Frontiers in Human Neuroscience*, *8*(July), 443.

Fleming, S. M., Weil, R. S., Nagy, Z., Dolan, R. J., & Rees, G. (2010). Relating introspective accuracy to individual differences in brain structure. *Science*, *329*(1541-1543), 1541–1543.

Galvin, S. J., Podd, J. V., Drga, V., & Whitmore, J. (2003). Type 2 tasks in the theory of signal detectability: discrimination between correct and incorrect decisions. *Psychonomic Bulletin & Review*, *10*(4), 843–

876.

Green, D. M., & Swets, J. A. (1966). *Signal Detection Theory and Psychophysics*. John Wiley & Sons, Inc.

Kellij, S., Fahrenfort, J., Lau, H., Peters, M. A. K., & Odegaard, B. (2020). An investigation of how relative precision of target encoding influences metacognitive performance. *Attention, Perception & Psychophysics*. https://doi.org/10.3758/s13414-020-02190-0

Macmillan, N. A., & Creelman, C. D. (2004). *Detection Theory: A User's Guide*. Taylor & Francis.

Maniscalco, B., & Lau, H. (2012). A signal detection theoretic approach for estimating metacognitive sensitivity from confidence ratings. *Consciousness and Cognition*, *21*(1), 422–430.

Maniscalco, B., & Lau, H. (2014). *Signal detection theory analysis of type 1 and type 2 data: meta-d', response-specific meta-d', and the unequal variance SDT mode* (S. M. Fleming & C. D. Frith (eds.); pp. 25–66). Springer.

Maniscalco, B., & Lau, H. (2016). The signal processing architecture underlying subjective reports of sensory awareness. *Neuroscience of Consciousness*, *November 2015*, 1–41.

Mazor, M., Friston, K. J., & Fleming, S. M. (2020). Distinct neural contributions to metacognition for detecting, but not discriminating visual stimuli. *eLife*, *9*, e53900.

Mazor, M., Moran, R., & Fleming, S. M. (2021). Metacognitive asymmetries in visual perception. *Neuroscience of Consciousness*, *2021*(2), niab025.

Peters, M. A. K., Fesi, J., Amendi, N., Knotts, J. D., Lau, H., & Ro, T. (2017). Transcranial magnetic stimulation to visual cortex induces suboptimal introspection. *Cortex; a Journal Devoted to the Study of the Nervous System and Behavior*, *93*, 119–132.

Pleskac, T. J., & Busemeyer, J. R. (2010). Two-stage dynamic signal detection: a theory of choice, decision time, and confidence. *Psychological Review*, *117*(3), 864–901.

Shekhar, M., & Rahnev, D. (2021a). Sources of Metacognitive Inefficiency. *Trends in Cognitive Sciences*, *25*(1), 12–23.

Shekhar, M., & Rahnev, D. (2021b). The nature of metacognitive inefficiency in perceptual decision making. *Psychological Review*, *128*(1), 45–70.
