# Supplementary material for: Optimal metacognitive decision strategies in signal detection theory
Source: Psychon Bull Rev. 2024 Nov 18;32(3):1041–69. doi: 10.3758/s13423-024-02510-7 (PMC12092500; doi:10.3758/s13423-024-02510-7)
Supplement: Supplementary file 2 — Supplementary file2 (PDF 341 KB) [file 13423_2024_2510_MOESM2_ESM.pdf]

Optimal Metacognitive Decision Strategies in Signal Detection Theory

Brian Maniscalco*, Lucie Charles*, & Megan Peters

Supplementary Material S2

Derivations of Optimal Type 1 and Type 2 Criteria

1. Optimizing Accuracy

1.1. Optimal Type 1 Criterion for Maximizing Type 1 Accuracy

Suppose an observer with perceptual sensitivity d' wishes to place the type 1 criterion c_1 so as to maximize the proportion of correct responses in discriminating two stimulus classes, S1 and S2, where p(S1) and p(S2) denote the prior probabilities of S1 and S2 being presented on a given trial. The measure to be optimized, proportion correct, can be expressed as

$$p(\text{correct}_1) = p(\text{S2})\ \text{HR}_1 + p(\text{S1})\ (1 - \text{FAR}_1) \tag{S1}$$

where HR_1 = type 1 hit rate = p(response = "S2" | stimulus = S2) and FAR_1 = type 1 false alarm rate = p(response = "S2" | stimulus = S1). For a given d' and c_1, HR_1 and FAR_1 can be expressed in the equal variance signal detection theory model as

$$\text{HR}_1 = 1 - \phi\left(c_1 \,\middle|\, \frac{d'}{2}\right) \tag{S2}$$

$$\text{FAR}_1 = 1 - \phi\left(c_1 \,\middle|\, -\frac{d'}{2}\right) \tag{S3}$$

where $\phi(x \mid \mu)$ is the cumulative distribution function of the normal distribution with mean μ and standard deviation $\sigma = 1$ evaluated at *x*. (Here we set $\sigma = 1$ for convenience, without loss of generality, as d' corresponds to the signal-to-noise ratio $\frac{\mu_{S2}-\mu_{S1}}{\sigma}$.)

To find the criterion c_1 that maximizes $p(\text{correct}_1)$, we set the derivative of $p(\text{correct}_1)$ with respect to c_1 equal to zero and solve for c_1. The derivative is given by

$$\frac{\mathrm{d}}{\mathrm{d}c_1}[p(\text{correct}_1)] = -p(\text{S2})\, \varphi\left(c_1 \,\middle|\, \frac{d'}{2}\right) + p(\text{S1})\, \varphi\left(c_1 \,\middle|\, -\frac{d'}{2}\right) \tag{S4}$$

where $\varphi(x \mid \mu)$ is the probability density function of the normal distribution with mean μ and standard deviation $\sigma = 1$ evaluated at *x*:

$$\varphi(x \mid \mu) = \frac{1}{\sqrt{2\pi}} e^{-\frac{(x-\mu)^2}{2}} \tag{S5}$$

Setting the derivative equal to zero and solving for c_1 gives

$$p(\text{S2})\, \varphi\left(c_1 \,\middle|\, \frac{d'}{2}\right) = p(\text{S1})\, \varphi\left(c_1 \,\middle|\, -\frac{d'}{2}\right)$$

$$\ln\frac{p(\text{S1})}{p(\text{S2})} = \ln\frac{\varphi\left(c_1 \,\middle|\, \frac{d'}{2}\right)}{\varphi\left(c_1 \,\middle|\, -\frac{d'}{2}\right)} = \frac{-\left(c_1 - \frac{d'}{2}\right)^2}{2} - \frac{-\left(c_1 + \frac{d'}{2}\right)^2}{2} = c_1 d'$$

$$c_1^{\mathrm{A}^*} = \frac{\ln\frac{p(\text{S1})}{p(\text{S2})}}{d'} \tag{S6}$$

where the A* superscript denotes that Equation S6 defines the optimal c_1 for maximizing type 1 accuracy (see **Table 1**, main manuscript).

1.2.1. Optimal Type 2 Criterion for Maximizing Type 2 Accuracy for “S2” Responses

Next, suppose the observer wishes to place the type 2 criterion for "S2" responses, $c_{2,\text{"S2"}}$, so as to maximize the proportion of correct type 2 responses when responding "S2." By way of analogy to the type 1 case, type 2 responses are considered to be correct when "high confidence" is reported for correct type 1 responses and when "low confidence" is reported for incorrect type 1 responses (see **Table 2**, main manuscript). More formally, the observer must set $c_{2,\text{"S2"}}$ so as to maximize $p(\text{correct}_{2,\text{"S2"}})$:

$$p\left(\text{correct}_{2,\text{"S2"}}\right) = p\left(\text{correct}_{1,\text{"S2"}}\right) \text{HR}_{2,\text{"S2"}} + p\left(\text{incorrect}_{1,\text{"S2"}}\right) \left(1 - \text{FAR}_{2,\text{"S2"}}\right) \qquad \text{(S7)}$$

where $\text{HR}_{2,\text{"S2"}}$ and $\text{FAR}_{2,\text{"S2"}}$ correspond to type 2 hit rate and false alarm rate for "S2" responses:

$$\text{HR}_{2,\text{"S2"}} = \frac{1 - \phi\left(c_{2,\text{"S2"}} \middle| \frac{d'}{2}\right)}{\text{HR}_1} \qquad \text{(S8)}$$

$$\text{FAR}_{2,\text{"S2"}} = \frac{1 - \phi\left(c_{2,\text{"S2"}} \middle| -\frac{d'}{2}\right)}{\text{FAR}_1} \qquad \text{(S9)}$$

and where $p(\text{correct}_{1,\text{"S2"}})$ and $p(\text{incorrect}_{1,\text{"S2"}})$ correspond to the probability of a correct or incorrect type 1 decision, given that the type 1 response was "S2":

$$p\left(\text{correct}_{1,\text{"S2"}}\right) = p(\text{stim} = \text{S2} \mid \text{resp} = \text{"S2"}) \qquad \text{(S10)}$$

$$p\left(\text{incorrect}_{1,\text{"S2"}}\right) = p(\text{stim} = \text{S1} \mid \text{resp} = \text{"S2"}) \qquad \text{(S11)}$$

Eqs. S10 and S11 can be re-expressed as

$$p\left(\text{correct}_{1,\text{"S2"}}\right) = \frac{p(\text{stim} = \text{S2} \cap \text{resp} = \text{"S2"})}{p(\text{resp} = \text{"S2"})} = \frac{p(\text{S2})\ \text{HR}_1}{p(\text{resp} = \text{"S2"})} \qquad \text{(S12)}$$

$$p\left(\text{incorrect}_{1,\text{"S2"}}\right) = \frac{p(\text{stim} = \text{S1} \cap \text{resp} = \text{"S2"})}{p(\text{resp} = \text{"S2"})} = \frac{p(\text{S1})\ \text{FAR}_1}{p(\text{resp} = \text{"S2"})} \qquad \text{(S13)}$$

Thus, substituting Eqs. S8-9 and S12-13 into Eq. S7, $p(\text{correct}_{2,\text{"S2"}})$ can be written

$$p(\text{correct}_{2,\text{"S2"}}) = \frac{p(\text{S2})\ \text{HR}_1}{p(\text{resp} = \text{"S2"})} \frac{1 - \phi\left(c_{2,\text{"S2"}} \middle| \frac{d'}{2}\right)}{\text{HR}_1} + \frac{p(\text{S1})\ \text{FAR}_1}{p(\text{resp} = \text{"S2"})} \left(1 - \left(\frac{1 - \phi\left(c_{2,\text{"S2"}} \middle| -\frac{d'}{2}\right)}{\text{FAR}_1}\right)\right)$$

Simplifying,

$$p(\text{correct}_{2,\text{"S2"}}) = \frac{p(\text{S2})}{p(\text{resp} = \text{"S2"})}\left(1 - \phi\left(c_{2,\text{"S2"}} \middle| \frac{d'}{2}\right)\right) + \frac{p(\text{S1})}{p(\text{resp} = \text{"S2"})}\left(\text{FAR}_1 - 1 + \phi\left(c_{2,\text{"S2"}} \middle| -\frac{d'}{2}\right)\right)$$

Differentiating with respect to $c_{2,\text{"S2"}}$ gives

$$\frac{\mathrm{d}}{\mathrm{d}c_{2,\text{"S2"}}}\left[p(\text{correct}_{2,\text{"S2"}})\right] = -\frac{p(\text{S2})}{p(\text{resp} = \text{"S2"})}\varphi\left(c_{2,\text{"S2"}} \middle| \frac{d'}{2}\right) + \frac{p(\text{S1})}{p(\text{resp} = \text{"S2"})}\varphi\left(c_{2,\text{"S2"}} \middle| -\frac{d'}{2}\right) \quad \text{(S14)}$$

Setting the derivative equal to zero and solving for $c_{2,\text{"S2"}}$ gives

$$\frac{p(\text{S2})}{p(\text{resp} = \text{"S2"})}\varphi\left(c_{2,\text{"S2"}} \middle| \frac{d'}{2}\right) = \frac{p(\text{S1})}{p(\text{resp} = \text{"S2"})}\varphi\left(c_{2,\text{"S2"}} \middle| -\frac{d'}{2}\right)$$

$$\log\frac{p(\text{S1})}{p(\text{S2})} = \ln\frac{\varphi\left(c_{2,\text{"S2"}} \middle| \frac{d'}{2}\right)}{\varphi\left(c_{2,\text{"S2"}} \middle| -\frac{d'}{2}\right)} = c_{2,\text{"S2"}}d'$$

$$c_{2,\text{"S2"}} = \frac{\ln \frac{p(\text{S1})}{p(\text{S2})}}{d'} \tag{S15}$$

Enforcing the constraint that $c_{2,\text{"S2"}} \geq c_1$ yields a final solution for $c_{2,\text{"S2"}}$ as

$$c_{2,\text{"S2"}}^{\text{A}^*} = \max\left(\frac{\ln \frac{p(\text{S1})}{p(\text{S2})}}{d'}, c_1\right) \tag{S16}$$

where the A* superscript denotes that Equation S16 defines the optimal $c_{2,\text{"S2"}}$ for maximizing type 2 accuracy (see **Table 1**, main manuscript).

1.2.2. Optimal Type 2 Criterion for Maximizing Type 2 Accuracy for "S1" Responses

For "S1" responses, the observer must set the type 2 criterion for "S1" responses, $c_{2,\text{"S1"}}$, so as to maximize p(correct$_{2,\text{"S1"}}$):

$$p(\text{correct}_{2,\text{"S1"}}) = p(\text{correct}_{1,\text{"S1"}})\,\text{HR}_{2,\text{"S1"}} + p(\text{incorrect}_{1,\text{"S1"}})\,(1 - \text{FAR}_{2,\text{"S1"}}) \tag{S17}$$

where

$$\text{HR}_{2,\text{"S1"}} = \frac{\Phi\left(c_{2,\text{"S1"}} \middle| -\frac{d'}{2}\right)}{(1 - \text{FAR}_1)} \tag{S18}$$

$$\text{FAR}_{2,\text{"S1"}} = \frac{\Phi\left(c_{2,\text{"S1"}} \middle| \frac{d'}{2}\right)}{(1 - \text{HR}_1)} \tag{S19}$$

$$p(\text{correct}_{1,\text{"S1"}}) = \frac{p(\text{S1})\,(1 - \text{FAR}_1)}{p(\text{resp} = \text{S1})} \tag{S20}$$

$$p\left(\text{incorrect}_{1,\text{"S1"}}\right) = \frac{p(\text{S2})\ (1 - \text{HR}_1)}{p(\text{resp} = \text{S1})} \tag{S21}$$

Substituting Eqs. S18-21 into Eq. S17, p(correct$_{2,\text{"S2"}}$) can be written

$$p\left(\text{correct}_{2,\text{"S1"}}\right) = \frac{p(\text{S1})\ (1 - \text{FAR}_1)}{p(\text{resp} = \text{"S1"})} \frac{\Phi\left(c_{2,\text{"S1"}} \middle| -\frac{d'}{2}\right)}{(1 - \text{FAR}_1)} + \frac{p(\text{S2})\ (1 - \text{HR}_1)}{p(\text{resp} = \text{"S1"})} \left(1 - \left(\frac{\Phi\left(c_{2,\text{"S1"}} \middle| \frac{d'}{2}\right)}{(1 - \text{HR}_1)}\right)\right)$$

which simplifies to

$$p\left(\text{correct}_{2,\text{"S1"}}\right) = \frac{p(\text{S1})}{p(\text{resp} = \text{"S1"})} \Phi\left(c_{2,\text{"S1"}} \middle| -\frac{d'}{2}\right) + \frac{p(\text{S2})}{p(\text{resp} = \text{S1})}\left((1 - \text{HR}_1) - \Phi\left(c_{2,\text{"S1"}} \middle| \frac{d'}{2}\right)\right)$$

Differentiating with respect to $c_{2,\text{"S1"}}$ gives

$$\frac{\mathrm{d}}{\mathrm{d}c_{2,\text{"S1"}}}\left[p\left(\text{correct}_{2,\text{"S1"}}\right)\right] = \frac{p(\text{S1})}{p(\text{resp} = \text{"S2"})}\ \varphi\left(c_{2,\text{"S1"}} \middle| -\frac{d'}{2}\right) - \frac{p(\text{S2})}{p(\text{resp} = \text{"S2"})}\ \varphi\left(c_{2,\text{"S1"}} \middle| \frac{d'}{2}\right) \tag{S22}$$

Setting the derivative equal to zero and solving for $c_{2,\text{"S1"}}$ gives

$$\frac{p(\text{S1})}{p(\text{resp} = \text{"S2"})}\ \varphi\left(c_{2,\text{"S1"}} \middle| -\frac{d'}{2}\right) = \frac{p(\text{S2})}{p(\text{resp} = \text{"S2"})}\ \varphi\left(c_{2,\text{"S1"}} \middle| \frac{d'}{2}\right)$$

$$\log\frac{p(\text{S1})}{p(\text{S2})} = \ln\frac{\varphi\left(c_{2,\text{"S1"}} \middle| \frac{d'}{2}\right)}{\varphi\left(c_{2,\text{"S1"}} \middle| -\frac{d'}{2}\right)} = c_{2,\text{"S1"}}d'$$

$$c_{2,\text{"S1"}} = \frac{\ln\frac{p(\text{S1})}{p(\text{S2})}}{d'} \tag{S23}$$

Enforcing the constraint that $c_{2,\text{"S1"}} \leq c_1$ yields a final solution for $c_{2,\text{"S1"}}$ as

$$c_{2,\text{"S1"}}^{\text{A}^*} = \min\left(\frac{\ln\frac{p(\text{S1})}{p(\text{S2})}}{d'}, c_1\right) \tag{S24}$$

where the A* superscript denotes that Equation S24 defines the optimal $c_{2,\text{"S1"}}$ for maximizing type 2 accuracy (see **Table 1**, main manuscript).

2. Optimizing Reward

2.1. Optimal Type 1 Criterion for Maximizing Type 1 Reward Contingencies

Suppose the observer wishes to place the type 1 criterion so as to maximize the reward earned via an environmental structure that differentially rewards different type 1 outcomes. The observer must set c_1 so as to maximize expected reward from a type 1 reward contingency table:

$$\text{E}(\text{reward}_1) = p(\text{S2})[R_{\text{hit1}}\text{HR}_1 + R_{\text{miss1}}(1 - \text{HR}_1)] + p(\text{S1})[R_{\text{CR1}}(1 - \text{FAR}_1) + R_{\text{FA1}}\text{FAR}_1] \tag{S25}$$

where R_{hit1}, R_{miss1}, R_{CR1}, and R_{FA1} correspond to the number of points gained or lost following type 1 hits, misses, correct rejections, and false alarms (see **Table 3**, main manuscript). To find the criterion c_1 that maximizes E(reward$_1$), we substitute for HR$_1$ and FAR$_1$ using Eqs. S2 and S3, set the derivative of E(reward$_1$) with respect to c_1 equal to zero, and solve for c_1. The derivative is given by

$$\begin{aligned}\frac{\text{d}}{\text{d}c_1}[\text{E}(\text{reward}_1)] = p(\text{S2})&\left[-R_{\text{hit1}}\varphi\left(c_1 \,\middle|\, \frac{d'}{2}\right) + R_{\text{miss1}}\varphi\left(c_1 \,\middle|\, \frac{d'}{2}\right)\right] \\ +p(\text{S1})&\left[R_{\text{CR1}}\varphi\left(c_1 \,\middle|\, -\frac{d'}{2}\right) - R_{\text{FA1}}\varphi\left(c_1 \,\middle|\, -\frac{d'}{2}\right)\right]\end{aligned} \tag{S26}$$

where $\varphi(x \mid \mu)$ is the probability density function of the normal distribution with mean μ and variance $\sigma = 1$ evaluated at x, as given in Eq. S5.

Setting the derivative equal to zero and solving for c_1 gives

$$p(\mathrm{S1})\left[(R_{\mathrm{CR1}}-R_{\mathrm{FA1}})\varphi\left(c_1\left|-\frac{d'}{2}\right.\right)\right]=p(\mathrm{S2})\left[(R_{\mathrm{hit1}}-R_{\mathrm{miss1}})\varphi\left(c_1\left|\frac{d'}{2}\right.\right)\right]$$

$$\ln\frac{p(\mathrm{S1})(R_{\mathrm{CR1}}-R_{\mathrm{FA1}})}{p(\mathrm{S2})(R_{\mathrm{hit1}}-R_{\mathrm{miss1}})}=\ln\frac{\varphi\left(c_1\left|\frac{d'}{2}\right.\right)}{\varphi\left(c_1\left|-\frac{d'}{2}\right.\right)}=c_1 d'$$

$$c_1^{\mathrm{R}^*}=\frac{\ln\frac{p(\mathrm{S1})}{p(\mathrm{S2})}+\ln\frac{(R_{\mathrm{CR1}}-R_{\mathrm{FA1}})}{(R_{\mathrm{hit1}}-R_{\mathrm{miss1}})}}{d'} \qquad \text{(S27)}$$

where the R* superscript denotes that Equation S27 defines the optimal c_1 for maximizing type 1 reward (see **Table 1**, main manuscript).

2.2.1. Optimal Type 2 Criterion for Maximizing Type 2 Reward Contingencies for "S2" Responses

Now suppose the observer wishes to maximize the reward earned via an environmental structure that differentially rewards different type 2 outcomes. For "S2" responses, the observer must set the type 2 criterion for "S2" responses, $c_{2,\text{"S2"}}$, so as to maximize E(reward$_{2,\text{"S2"}}$):

$$\begin{aligned}\mathrm{E}\big(\mathrm{reward}_{2,\text{"S2"}}\big)=&\ p(\mathrm{hit}_1,\mathrm{LC}|\text{"S2"})R_{\mathrm{miss2}}+p(\mathrm{hit}_1,\mathrm{HC}|\text{"S2"})R_{\mathrm{hit2}}\\&+p(\mathrm{FA}_1,\mathrm{LC}|\text{"S2"})R_{\mathrm{CR2}}+p(\mathrm{FA}_1,\mathrm{HC}|\text{"S2"})R_{\mathrm{FA2}}\end{aligned} \qquad \text{(S28)}$$

where R_{hit2}, R_{miss2}, R_{CR2}, and R_{FA2} correspond to the number of points gained or lost following type 2 hits, misses, correct rejections, and false alarms (see **Table 4**, main manuscript), and where e.g. $p(\mathrm{hit}_1$, LC | "S2") is the probability of a low confidence hit given that the observer responded "S2," or more formally,

$$\begin{aligned}p(\mathrm{hit}_1,\mathrm{LC}|\text{"S2"})&=p(\mathrm{stim}=\mathrm{S2},\mathrm{conf}=\mathrm{low}\mid\mathrm{resp}=\text{"S2"})\\&=\frac{p(\mathrm{stim}=\mathrm{S2}\ \cap\mathrm{resp}=\text{"S2"}\cap\mathrm{conf}=\mathrm{low})}{p(\mathrm{resp}=\text{"S2"})}\end{aligned}$$

This expression can be re-written as

$$p(\mathrm{hit}_1, \mathrm{LC}|"\mathrm{S2}") = \frac{p(\mathrm{S2})\ \mathrm{HR}_1\ (1 - \mathrm{HR}_{2,"\mathrm{S2}"})}{p(\mathrm{resp} = "\mathrm{S2}")} \tag{S29}$$

and similarly,

$$p(\mathrm{hit}_1, \mathrm{HC}|"\mathrm{S2}") = \frac{p(\mathrm{S2})\ \mathrm{HR}_1\ \mathrm{HR}_{2,"\mathrm{S2}"}}{p(\mathrm{resp} = "\mathrm{S2}")} \tag{S30}$$

$$p(\mathrm{FA}_1, \mathrm{LC}|"\mathrm{S2}") = \frac{p(\mathrm{S1})\ \mathrm{FAR}_1\ (1 - \mathrm{FAR}_{2,"\mathrm{S2}"})}{p(\mathrm{resp} = "\mathrm{S2}")} \tag{S31}$$

$$p(\mathrm{FA}_1, \mathrm{HC}|"\mathrm{S2}") = \frac{p(\mathrm{S1})\ \mathrm{FAR}_1\ \mathrm{FAR}_{2,"\mathrm{S2}"}}{p(\mathrm{resp} = "\mathrm{S2}")} \tag{S32}$$

Rearranging the equation for $\mathrm{E}(\mathrm{reward}_{2,"S2"})$ (Eq. S28) and using Eqs. S8-9 to substitute for the $\mathrm{HR}_{2,"S2"}$ and $\mathrm{FAR}_{2,"S2"}$ terms gives

$$\begin{aligned}
\mathrm{E}\left(\mathrm{reward}_{2,"\mathrm{S2}"}\right)
&= \frac{p(\mathrm{S2})\ \mathrm{HR}_1\ (1 - \mathrm{HR}_{2,"\mathrm{S2}"})}{p(\mathrm{resp} = "\mathrm{S2}")}\ R_{\mathrm{miss2}} + \frac{p(\mathrm{S2})\ \mathrm{HR}_1\ \mathrm{HR}_{2,"\mathrm{S2}"}}{p(\mathrm{resp} = "\mathrm{S2}")}\ R_{\mathrm{hit2}} \\
&+ \frac{p(\mathrm{S1})\ \mathrm{FAR}_1\ (1 - \mathrm{FAR}_{2,"\mathrm{S2}"})}{p(\mathrm{resp} = "\mathrm{S2}")} R_{\mathrm{CR2}} + \frac{p(\mathrm{S1})\ \mathrm{FAR}_1\ \mathrm{FAR}_{2,"\mathrm{S2}"}}{p(\mathrm{resp} = "\mathrm{S2}")}\ R_{\mathrm{FA2}}
\end{aligned}$$

$$\begin{aligned}
\mathrm{E}\left(\mathrm{reward}_{2,"\mathrm{S2}"}\right)
&= \frac{p(\mathrm{S2})\ \mathrm{HR}_1}{p(\mathrm{resp} = "\mathrm{S2}")}\ \left[(1 - \mathrm{HR}_{2,"\mathrm{S2}"})R_{\mathrm{miss2}} + \mathrm{HR}_{2,"\mathrm{S2}"}R_{\mathrm{hit2}}\right] \\
&+ \frac{p(\mathrm{S1})\ \mathrm{FAR}_1}{p(\mathrm{resp} = "\mathrm{S2}")}\ \left[(1 - \mathrm{FAR}_{2,"\mathrm{S2}"})R_{\mathrm{CR2}} + \mathrm{FAR}_{2,"\mathrm{S2}"}R_{\mathrm{FA2}}\right]
\end{aligned}$$

$$E(\text{reward}_{2,\text{"S2"}}) = \frac{p(\text{S2})\ \text{HR}_1}{p(\text{resp} = \text{"S2"})}\left[\left(1 - \left(\frac{1 - \phi\left(c_{2,\text{"S2"}}\,\middle|\,\frac{d'}{2}\right)}{\text{HR}_1}\right)\right)R_{\text{miss2}} + \frac{1 - \phi\left(c_{2,\text{"S2"}}\,\middle|\,\frac{d'}{2}\right)}{\text{HR}_1}R_{\text{hit2}}\right] + \frac{p(\text{S1})\ \text{FAR}_1}{p(\text{resp} = \text{"S2"})}\left[\left(1 - \left(\frac{1 - \phi\left(c_{2,\text{"S2"}}\,\middle|\,-\frac{d'}{2}\right)}{\text{FAR}_1}\right)\right)R_{\text{CR2}} + \frac{1 - \phi\left(c_{2,\text{"S2"}}\,\middle|\,-\frac{d'}{2}\right)}{\text{FAR}_1}R_{\text{FA2}}\right]$$

$$E(\text{reward}_{2,\text{"S2"}}) = \frac{p(\text{S2})\ \text{HR}_1}{p(\text{resp} = \text{"S2"})}\left[\frac{\text{HR}_1 - 1 + \phi\left(c_{2,\text{"S2"}}\,\middle|\,\frac{d'}{2}\right)}{\text{HR}_1}R_{\text{miss2}} + \frac{1 - \phi\left(c_{2,\text{"S2"}}\,\middle|\,\frac{d'}{2}\right)}{\text{HR}_1}R_{\text{hit2}}\right] + \frac{p(\text{S1})\ \text{FAR}_1}{p(\text{resp} = \text{"S2"})}\left[\frac{\text{FAR}_1 - 1 + \phi\left(c_{2,\text{"S2"}}\,\middle|\,-\frac{d'}{2}\right)}{\text{FAR}_1}R_{\text{CR2}} + \frac{1 - \phi\left(c_{2,\text{"S2"}}\,\middle|\,-\frac{d'}{2}\right)}{\text{FAR}_1}R_{\text{FA2}}\right]$$

$$E(\text{reward}_{2,\text{"S2"}}) = \frac{p(\text{S2})}{p(\text{resp} = \text{"S2"})}\left[\left(\text{HR}_1 - 1 + \phi\left(c_{2,\text{"S2"}}\,\middle|\,\frac{d'}{2}\right)\right)R_{\text{miss2}} + \left(1 - \phi\left(c_{2,\text{"S2"}}\,\middle|\,\frac{d'}{2}\right)\right)R_{\text{hit2}}\right] + \frac{p(\text{S1})}{p(\text{resp} = \text{"S2"})}\left[\left(\text{FAR}_1 - 1 + \phi\left(c_{2,\text{"S2"}}\,\middle|\,-\frac{d'}{2}\right)\right)R_{\text{CR2}} + \left(1 - \phi\left(c_{2,\text{"S2"}}\,\middle|\,-\frac{d'}{2}\right)\right)R_{\text{FA2}}\right]$$

Differentiating with respect to $c_{2,\text{"S2"}}$ gives

$$\frac{\mathrm{d}}{\mathrm{d}c_{2,\text{"S2"}}}\left[E(\text{reward}_{2,\text{"S2"}})\right] = \frac{p(\text{S2})}{p(\text{resp} = \text{"S2"})}\left[\varphi\left(c_{2,\text{"S2"}}\,\middle|\,\frac{d'}{2}\right)R_{\text{miss2}} - \varphi\left(c_{2,\text{"S2"}}\,\middle|\,\frac{d'}{2}\right)R_{\text{hit2}}\right] + \frac{p(\text{S1})}{p(\text{resp} = \text{"S2"})}\left[\varphi\left(c_{2,\text{"S2"}}\,\middle|\,-\frac{d'}{2}\right)R_{\text{CR2}} - \varphi\left(c_{2,\text{"S2"}}\,\middle|\,-\frac{d'}{2}\right)R_{\text{FA2}}\right] \quad \text{(S33)}$$

Setting the derivative equal to zero and solving for $c_{2,\text{"S2"}}$ gives

$$-p(\text{S2})\ \varphi\left(c_{2,\text{"S2"}}\left|\frac{d'}{2}\right.\right)(R_{\text{miss2}}-R_{\text{hit2}}) = \ p(\text{S1})\ \varphi\left(c_{2,\text{"S2"}}\left|-\frac{d'}{2}\right.\right)(R_{\text{CR2}}-R_{\text{FA2}})$$

$$\ln\frac{p(\text{S1})(R_{\text{CR2}}-R_{\text{FA2}})}{p(\text{S2})(R_{\text{hit2}}-R_{\text{miss2}})} = \ln\frac{\varphi\left(c_{2,\text{"S2"}}\left|\frac{d'}{2}\right.\right)}{\varphi\left(c_{2,\text{"S2"}}\left|-\frac{d'}{2}\right.\right)} = c_{2,\text{"S2"}}d'$$

$$c_{2,\text{"S2"}} = \frac{\ln\frac{p(\text{S1})}{p(\text{S2})}+\ln\frac{(R_{\text{CR2}}-R_{\text{FA2}})}{(R_{\text{hit2}}-R_{\text{miss2}})}}{d'} \tag{S34}$$

Enforcing the constraint that $c_{2,\text{"S2"}} \geq c_1$ yields a final solution for $c_{2,\text{"S2"}}$ as

$$c_{2,\text{"S2"}}^{\text{R}^*} = \max\left(\frac{\ln\frac{p(\text{S1})}{p(\text{S2})}+\ln\frac{(R_{\text{CR2}}-R_{\text{FA2}})}{(R_{\text{hit2}}-R_{\text{miss2}})}}{d'}, c_1\right) \tag{S35}$$

where the R* superscript denotes that Equation S35 defines the optimal $c_{2,\text{"S2"}}$ for maximizing type 2 reward (see **Table 1**, main manuscript).

2.2.2. Optimal type 2 Criterion for Maximizing Type 2 Reward Contingencies for "S1" Responses

For "S1" responses, the observer must set the type 2 criterion for "S1" responses, $c_{2,\text{"S1"}}$, so as to maximize E(reward$_{2,\text{"S1"}}$):

$$\begin{aligned}\text{E}\big(\text{reward}_{2,\text{"S1"}}\big) = p(\text{CR}_1,\text{LC}|\text{"S1"})R_{\text{miss2}} + p(\text{CR}_1,\text{HC}|\text{"S1"})R_{\text{hit2}} \\ +p(\text{miss}_1,\text{LC}|\text{"S1"})R_{\text{CR2}} + p(\text{miss}_1,\text{HC}|\text{"S1"})R_{\text{FA2}}\end{aligned} \tag{S36}$$

where e.g. $p(\text{CR}_1, \text{LC} \mid \text{"S1"})$ is the probability of a low confidence correct rejection given that the observer responded "S1." By similar reasoning for the derivation of Eq. S29, we can write

$$p(\mathrm{CR}_1, \mathrm{LC}|\text{"S1"}) = \frac{p(\mathrm{S1})\,(1-\mathrm{FAR}_1)\,(1-\mathrm{HR}_{2,\text{"S1"}})}{p(\mathrm{resp}=\text{"S1"})} \tag{S37}$$

$$p(\mathrm{CR}_1, \mathrm{HC}|\text{"S1"}) = \frac{p(\mathrm{S1})\,(1-\mathrm{FAR}_1)\,\mathrm{HR}_{2,\text{"S1"}}}{p(\mathrm{resp}=\text{"S1"})} \tag{S38}$$

$$p(\mathrm{miss}_1, \mathrm{LC}|\text{"S1"}) = \frac{p(\mathrm{S2})\,(1-\mathrm{HR}_1)\,(1-\mathrm{FAR}_{2,\text{"S1"}})}{p(\mathrm{resp}=\text{"S1"})} \tag{S39}$$

$$p(\mathrm{miss}_1, \mathrm{HC}|\text{"S1"}) = \frac{p(\mathrm{S2})\,(1-\mathrm{HR}_1)\,\mathrm{FAR}_{2,\text{"S1"}}}{p(\mathrm{resp}=\text{"S1"})} \tag{S40}$$

Rearranging the equation for E(reward$_{2,\text{"S1"}}$) (Eq. S36) and using Eqs. S18-19 to substitute for the HR$_{2,\text{"S1"}}$ and FAR$_{2,\text{"S1"}}$ terms gives

$$\begin{aligned}\mathrm{E}(\mathrm{reward}_{2,\text{"S1"}}) \\ = \frac{p(\mathrm{S1})\,(1-\mathrm{FAR}_1)\,(1-\mathrm{HR}_{2,\text{"S1"}})}{p(\mathrm{resp}=\text{"S1"})} R_{\mathrm{miss2}} + \frac{p(\mathrm{S1})\,(1-\mathrm{FAR}_1)\,\mathrm{HR}_{2,\text{"S1"}}}{p(\mathrm{resp}=\text{"S1"})} R_{\mathrm{hit2}} \\ + \frac{p(\mathrm{S2})\,(1-\mathrm{HR}_1)\,(1-\mathrm{FAR}_{2,\text{"S1"}})}{p(\mathrm{resp}=\text{"S1"})} R_{\mathrm{CR2}} + \frac{p(\mathrm{S2})\,(1-\mathrm{HR}_1)\,\mathrm{FAR}_{2,\text{"S1"}}}{p(\mathrm{resp}=\text{"S1"})} R_{\mathrm{FA2}}\end{aligned}$$

$$\begin{aligned}\mathrm{E}(\mathrm{reward}_{2,\text{"S1"}}) \\ = \frac{p(\mathrm{S1})\,(1-\mathrm{FAR}_1)}{p(\mathrm{resp}=\text{"S1"})}\left[(1-\mathrm{HR}_{2,\text{"S1"}})R_{\mathrm{miss2}} + \mathrm{HR}_{2,\text{"S1"}}R_{\mathrm{hit2}}\right] \\ + \frac{p(\mathrm{S2})\,(1-\mathrm{HR}_1)}{p(\mathrm{resp}=\text{"S1"})}\left[(1-\mathrm{FAR}_{2,\text{"S1"}})R_{\mathrm{CR2}} + \mathrm{FAR}_{2,\text{"S1"}}R_{\mathrm{FA2}}\right]\end{aligned}$$

$$E(\text{reward}_{2,\text{"S1"}}) = \frac{p(\text{S1})\,(1-\text{FAR}_1)}{p(\text{resp}=\text{"S1"})}\left[\left(1-\left(\frac{\phi\left(c_{2,\text{"S1"}}\,\middle|\,-\frac{d'}{2}\right)}{(1-\text{FAR}_1)}\right)\right)R_{\text{miss2}} + \frac{\phi\left(c_{2,\text{"S1"}}\,\middle|\,-\frac{d'}{2}\right)}{(1-\text{FAR}_1)}R_{\text{hit2}}\right] + \frac{p(\text{S2})\,(1-\text{HR}_1)}{p(\text{resp}=\text{"S1"})}\left[\left(1-\left(\frac{\phi\left(c_{2,\text{"S1"}}\,\middle|\,\frac{d'}{2}\right)}{(1-\text{HR}_1)}\right)\right)R_{\text{CR2}} + \frac{\phi\left(c_{2,\text{"S1"}}\,\middle|\,\frac{d'}{2}\right)}{(1-\text{HR}_1)}R_{\text{FA2}}\right]$$

$$E(\text{reward}_{2,\text{"S1"}}) = \frac{p(\text{S1})}{p(\text{resp}=\text{"S1"})}\left[\left((1-\text{FAR}_1)-\phi\left(c_{2,\text{"S1"}}\,\middle|\,-\frac{d'}{2}\right)\right)R_{\text{miss2}} + \phi\left(c_{2,\text{"S1"}}\,\middle|\,-\frac{d'}{2}\right)R_{\text{hit2}}\right] + \frac{p(\text{S2})}{p(\text{resp}=\text{"S1"})}\left[\left((1-\text{HR}_1)-\phi\left(c_{2,\text{"S1"}}\,\middle|\,\frac{d'}{2}\right)\right)R_{\text{CR2}} + \phi\left(c_{2,\text{"S1"}}\,\middle|\,\frac{d'}{2}\right)R_{\text{FA2}}\right]$$

Differentiating with respect to $c_{2,\text{"S1"}}$ gives

$$\frac{\mathrm{d}}{\mathrm{d}c_{2,\text{"S1"}}}\left[E(\text{reward}_{2,\text{"S1"}})\right] = \frac{p(\text{S1})}{p(\text{resp}=\text{"S1"})}\left[-\varphi\left(c_{2,\text{"S1"}}\,\middle|\,-\frac{d'}{2}\right)R_{\text{miss2}} + \varphi\left(c_{2,\text{"S1"}}\,\middle|\,-\frac{d'}{2}\right)R_{\text{hit2}}\right] + \frac{p(\text{S2})}{p(\text{resp}=\text{"S1"})}\left[-\varphi\left(c_{2,\text{"S1"}}\,\middle|\,\frac{d'}{2}\right)R_{\text{CR2}} + \varphi\left(c_{2,\text{"S1"}}\,\middle|\,\frac{d'}{2}\right)R_{\text{FA2}}\right] \quad \text{(S41)}$$

Setting the derivative equal to zero and solving for $c_{2,\text{"S1"}}$ gives

$$p(\text{S1})\,\varphi\left(c_{2,\text{"S1"}}\,\middle|\,-\frac{d'}{2}\right)(R_{\text{hit2}}-R_{\text{miss2}}) = -p(\text{S2})\,\varphi\left(c_{2,\text{"S1"}}\,\middle|\,\frac{d'}{2}\right)(R_{\text{FA2}}-R_{\text{CR2}})$$

$$\ln\frac{p(\text{S1})(R_{\text{hit2}}-R_{\text{miss2}})}{p(\text{S2})(R_{\text{CR2}}-R_{\text{FA2}})} = \ln\frac{\varphi\left(c_{2,\text{"S1"}}\,\middle|\,\frac{d'}{2}\right)}{\varphi\left(c_{2,\text{"S1"}}\,\middle|\,-\frac{d'}{2}\right)} = c_{2,\text{"S1"}}d'$$

$$c_{2,\text{"S1"}} = \frac{\ln \frac{p(\text{S1})}{p(\text{S2})} + \ln \frac{(R_{\text{hit2}} - R_{\text{miss2}})}{(R_{\text{CR2}} - R_{\text{FA2}})}}{d'} \quad \text{(S42)}$$

Enforcing the constraint that $c_{2,\text{"S1"}} \leq c_1$ yields a final solution for $c_{2,\text{"S1"}}$ as

$$c_{2,\text{"S1"}}^{\text{R}*} = \min\left(\frac{\ln \frac{p(\text{S1})}{p(\text{S2})} + \ln \frac{(R_{\text{hit2}} - R_{\text{miss2}})}{(R_{\text{CR2}} - R_{\text{FA2}})}}{d'}, c_1 \right) \quad \text{(S43)}$$

where the R* superscript denotes that Equation S43 defines the optimal $c_{2,\text{"S1"}}$ for maximizing type 2 reward (see **Table 1**, main manuscript).

3. Optimizing Calibration

(In the below we intentionally decline to include a **Section 3.1**, as optimizing type 2 calibration does not have a natural analogue in the type 1 case.)

Suppose the observer wishes to place high confidence ratings to reflect that some benchmark of type 1 accuracy has been achieved. For instance, the observer may choose to rate "high confidence" only when the estimated probability of a correct type 1 choice, $p(\text{correct}_1)$, exceeds 0.8.

More formally, let $p(\text{correct}_1)_\text{T}$ be the threshold value of accuracy needed to report high confidence (where T denotes threshold), and let $p(\text{correct}_1|x)$ be the observer's estimate of being correct on the current trial based upon the evidence sample *x*. Then the observer seeking to calibrate confidence to accuracy uses the following decision policy for rating confidence:

$$\text{confidence} = \begin{cases} \text{high,} & \text{if } p(\text{correct}_1 \mid x) > p(\text{correct}_1)_\text{T} \\ \text{low,} & \text{if } p(\text{correct}_1 \mid x) \leq \text{p}(\text{correct}_1)_\text{T} \end{cases} \quad \text{(S44)}$$

The formulae for the type 2 criteria that achieve this decision policy differ for "S1" and "S2" responses.

3.2.1. Optimal Type 2 Criterion for Calibrating "S2" Responses

On trials where the observer responds "S2," the quantity the observer must consider in order to calibrate confidence to accuracy is $p(\text{correct}_{1,\text{"S2"}}|x)$, where $p(\text{correct}_{1,\text{"S2"}})$ denotes probability of a correct type 1 response, given that the response was "S2". Since "S2" responses are correct whenever the stimulus is S2, it follows that $p(\text{correct}_{1,\text{"S2"}}|x) = p(\text{stim=S2}|x)$, where $p(\text{stim=S2}|x)$ is given by the relative likelihood that x was generated by S2 rather than S1:

$$p\left(\text{correct}_{1,\text{"S2"}} \mid x\right) = p(\text{stim} = \text{S2} \mid x) = \frac{p(\text{S2})\ \varphi\left(x \,\middle|\, \frac{d'}{2}\right)}{p(\text{S1})\ \varphi\left(x \,\middle|\, -\frac{d'}{2}\right) + p(\text{S2})\ \varphi\left(x \,\middle|\, \frac{d'}{2}\right)} \quad \text{(S45)}$$

Following Eq. S44, the observer must report high confidence for "S2" responses when $p(\text{correct}_{1,\text{"S2"}}|x) > p(\text{correct}_1)_\text{T}$. This objective can be accomplished by setting the type 2 criterion $c_{2,\text{"S2"}}$ at the location of the decision axis value x where $p(\text{correct}_{1,\text{"S2"}}|x) = p(\text{correct}_1)_\text{T}$:

$$p(\text{correct}_1)_\text{T} = \frac{p(\text{S2})\ \varphi\left(c_{2,\text{"S2"}} \,\middle|\, \frac{d'}{2}\right)}{p(\text{S1})\ \varphi\left(c_{2,\text{"S2"}} \,\middle|\, -\frac{d'}{2}\right) + p(\text{S2})\ \varphi\left(c_{2,\text{"S2"}} \,\middle|\, \frac{d'}{2}\right)} \quad \text{(S46)}$$

Solving for $c_{2,\text{"S2"}}$,

$$\frac{1}{p(\text{correct}_1)_\text{T}} = \frac{p(\text{S1})\ \varphi\left(c_{2,\text{"S2"}} \,\middle|\, -\frac{d'}{2}\right) + p(\text{S2})\ \varphi\left(c_{2,\text{"S2"}} \,\middle|\, \frac{d'}{2}\right)}{p(\text{S2})\ \varphi\left(c_{2,\text{"S2"}} \,\middle|\, \frac{d'}{2}\right)} = \frac{p(\text{S1})\ \varphi\left(c_{2,\text{"S2"}} \,\middle|\, -\frac{d'}{2}\right)}{p(\text{S2})\ \varphi\left(c_{2,\text{"S2"}} \,\middle|\, \frac{d'}{2}\right)} + 1$$

$$\ln\frac{1 - p(\text{correct}_1)_\text{T}}{p(\text{correct}_1)_\text{T}} = \ln\frac{p(\text{S1})\ \varphi\left(c_{2,\text{"S2"}} \,\middle|\, -\frac{d'}{2}\right)}{p(\text{S2})\ \varphi\left(c_{2,\text{"S2"}} \,\middle|\, \frac{d'}{2}\right)} = \ln\frac{p(\text{S1})}{p(\text{S2})} + \ln\frac{\varphi\left(c_{2,\text{"S2"}} \,\middle|\, -\frac{d'}{2}\right)}{\varphi\left(c_{2,\text{"S2"}} \,\middle|\, \frac{d'}{2}\right)}$$

$$\ln\frac{p(\text{S1})}{p(\text{S2})} + \ln\frac{p(\text{correct}_1)_\text{T}}{1 - p(\text{correct}_1)_\text{T}} = c_{2,\text{"S2"}}d'$$

$$c_{2,\text{"S2"}} = \frac{\ln\frac{p(\text{S1})}{p(\text{S2})} + \ln\frac{p(\text{correct}_1)_\text{T}}{1 - p(\text{correct}_1)_\text{T}}}{d'} \quad \text{(S47)}$$

Enforcing the constraint that $c_{2,"S2"} \geq c_1$ yields a final solution for $c_{2,"S2"}$ as

$$c^{C^*}_{2,"S2"} = \max\left(\frac{\ln\frac{p(\mathrm{S1})}{p(\mathrm{S2})} + \ln\frac{p(\mathrm{correct}_1)_\mathrm{T}}{1-p(\mathrm{correct}_1)_\mathrm{T}}}{d'}, c_1\right) \tag{S48}$$

where the C* superscript denotes that Equation S48 defines the optimal $c_{2,"S2"}$ for maximizing type 2 calibration (see **Table 1**, main manuscript).

3.2.2. Optimal Type 2 Criterion for Calibrating "S1" Responses

Following similar logic as for "S2" responses, the probability of being correct given a particular evidence value *x* for an "S1" response is given by

$$p(\mathrm{correct}_{1,"S1"} \mid x) = p(\mathrm{stim} = \mathrm{S1} \mid x) = \frac{p(\mathrm{S1})\ \varphi\left(x \middle| -\frac{d'}{2}\right)}{p(\mathrm{S1})\ \varphi\left(x \middle| -\frac{d'}{2}\right) + p(\mathrm{S2})\ \varphi\left(x \middle| \frac{d'}{2}\right)} \tag{S49}$$

Following Eq. S44, the observer must report high confidence for "S1" responses when $p(\mathrm{correct}_{1,"S1"}|x) > p(\mathrm{correct}_1)_\mathrm{T}$. This objective can be accomplished by setting the type 2 criterion $c_{2,"S1"}$ at the location of the decision axis value *x* where $p(\mathrm{correct}_{1,"S1"}|x) = p(\mathrm{correct}_1)_\mathrm{T}$:

$$p(\mathrm{correct}_1)_\mathrm{T} = \frac{p(\mathrm{S1})\ \varphi\left(c_{2,"S1"} \middle| -\frac{d'}{2}\right)}{p(\mathrm{S1})\ \varphi\left(c_{2,"S1"} \middle| -\frac{d'}{2}\right) + p(\mathrm{S2})\ \varphi\left(c_{2,"S1"} \middle| \frac{d'}{2}\right)} \tag{S50}$$

Solving for $c_{2,"S1"}$,

$$\frac{1}{p(\mathrm{correct}_1)_\mathrm{T}} = \frac{p(\mathrm{S1})\ \varphi\left(c_{2,"S1"} \middle| -\frac{d'}{2}\right) + p(\mathrm{S2})\ \varphi\left(c_{2,"S1"} \middle| \frac{d'}{2}\right)}{p(\mathrm{S1})\ \varphi\left(c_{2,"S1"} \middle| -\frac{d'}{2}\right)} = \frac{p(\mathrm{S2})\ \varphi\left(c_{2,"S1"} \middle| \frac{d'}{2}\right)}{p(\mathrm{S1})\ \varphi\left(c_{2,"S1"} \middle| -\frac{d'}{2}\right)} + 1$$

$$\ln\frac{1-p(\text{correct}_1)_\text{T}}{p(\text{correct}_1)_\text{T}} = \ln\frac{p(\text{S2})\,\varphi\left(c_{2,\text{"S1"}}\,\middle|\,\frac{d'}{2}\right)}{p(\text{S1})\,\varphi\left(c_{2,\text{"S1"}}\,\middle|\,-\frac{d'}{2}\right)} = \ln\frac{p(\text{S2})}{p(\text{S1})} + \ln\frac{\varphi\left(c_{2,\text{"S1"}}\,\middle|\,\frac{d'}{2}\right)}{\varphi\left(c_{2,\text{"S1"}}\,\middle|\,-\frac{d'}{2}\right)}$$

$$\ln\frac{p(\text{S1})}{p(\text{S2})} + \ln\frac{1-p(\text{correct}_1)_\text{T}}{p(\text{correct}_1)_\text{T}} = c_{2,\text{"S1"}}d'$$

$$c_{2,\text{"S1"}} = \frac{\ln\frac{p(\text{S1})}{p(\text{S2})} + \ln\frac{1-p(\text{correct}_1)_\text{T}}{p(\text{correct}_1)_\text{T}}}{d'} \qquad \text{(S51)}$$

Enforcing the constraint that $c_{2,\text{"S1"}} \leq c_1$ yields a final solution for $c_{2,\text{"S1"}}$ as

$$c_{2,\text{"S1"}}^{\text{C}^*} = \min\left(\frac{\ln\frac{p(\text{S1})}{p(\text{S2})} + \ln\frac{1-p(\text{correct}_1)_\text{T}}{p(\text{correct}_1)_\text{T}}}{d'}, c_1\right) \qquad \text{(S52)}$$

where the C* superscript denotes that Equation S52 defines the optimal $c_{2,\text{"S1"}}$ for maximizing type 2 calibration (see **Table 1**, main manuscript).

4. Optimizing HR – FAR

4.1. Optimal Type 1 Criterion for Maximizing HR_1 – FAR_1

Suppose the observer has the objective of maximizing the difference between type 1 hit rate and type 1 false alarm rate:

$$D_1 = \text{HR}_1 - \text{FAR}_1 \qquad \text{(S53)}$$

where HR_1 and FAR_1 are defined as in Eqs. S2 and S3, respectively. Thus, D_1 can be written

$$D_1 = 1 - \phi\left(c_1 \middle| \frac{d'}{2}\right) - \left(1 - \phi\left(c_1 \middle| -\frac{d'}{2}\right)\right) = -\phi\left(c_1 \middle| \frac{d'}{2}\right) + \phi\left(c_1 \middle| -\frac{d'}{2}\right)$$

Differentiating with respect to c_1 gives

$$\frac{\mathrm{d}}{\mathrm{d}c_1}[D_1] = -\varphi\left(c_1 \middle| \frac{d'}{2}\right) + \varphi\left(c_1 \middle| -\frac{d'}{2}\right) \tag{S54}$$

Setting the derivative equal to zero and solving for c_1 gives

$$\varphi\left(c_1 \middle| \frac{d'}{2}\right) = \varphi\left(c_1 \middle| -\frac{d'}{2}\right)$$

$$\ln \frac{\varphi\left(c_1 \middle| \frac{d'}{2}\right)}{\varphi\left(c_1 \middle| -\frac{d'}{2}\right)} = \ln 1$$

$$c_1 d' = 0$$

$$c_1^{\mathrm{HF}^*} = 0 \tag{S55}$$

where the HF* superscript denotes that Equation S55 defines the optimal c_1 for maximizing the difference between type 1 hit rate and type 1 false alarm rate (see **Table 1**, main manuscript).

4.2.1. Optimal Type 2 Criterion for Maximizing HR_2 – FAR_2 for "S2" Responses

Suppose the observer has the objective of maximizing the difference between type 2 hit rate and type 2 false alarm rate:

$$D_2 = \mathrm{HR}_2 - \mathrm{FAR}_2 \tag{S56}$$

For “S2” responses, the observer must set the type 2 criterion for “S2” responses, $c_{2,\text{"S2"}}$, so as to maximize $D_{2,\text{"S2"}}$:

$$D_{2,\text{"S2"}} = \mathrm{HR}_{2,\text{"S2"}} - \mathrm{FAR}_{2,\text{"S2"}} \tag{S57}$$

where $\mathrm{HR}_{2,\text{"S2"}}$ and $\mathrm{FAR}_{2,\text{"S2"}}$ are defined as in Eqs. S8 and S9, respectively. Thus, $D_{2,\text{"S2"}}$ can be written

$$D_{2,\text{"S2"}} = \frac{1 - \phi\left(c_{2,\text{"S2"}} \,\middle|\, \frac{d'}{2}\right)}{\mathrm{HR}_1} - \frac{1 - \phi\left(c_{2,\text{"S2"}} \,\middle|\, -\frac{d'}{2}\right)}{\mathrm{FAR}_1} \tag{S58}$$

Differentiating with respect to $c_{2,\text{"S2"}}$ gives

$$\frac{\mathrm{d}}{\mathrm{d}c_{2,\text{"S2"}}}\left[D_{2,\text{"S2"}}\right] = -\frac{1}{\mathrm{HR}_1}\varphi\left(c_{2,\text{"S2"}} \,\middle|\, \frac{d'}{2}\right) + \frac{1}{\mathrm{FAR}_1}\varphi\left(c_{2,\text{"S2"}} \,\middle|\, -\frac{d'}{2}\right) \tag{S59}$$

Setting the derivative equal to zero and solving for $c_{2,\text{"S2"}}$ gives

$$\frac{1}{\mathrm{HR}_1}\varphi\left(c_{2,\text{"S2"}} \,\middle|\, \frac{d'}{2}\right) = \frac{1}{\mathrm{FAR}_1}\varphi\left(c_{2,\text{"S2"}} \,\middle|\, -\frac{d'}{2}\right)$$

$$\ln\frac{\mathrm{HR}_1}{\mathrm{FAR}_1} = \ln\frac{\varphi\left(c_{2,\text{"S2"}} \,\middle|\, \frac{d'}{2}\right)}{\varphi\left(c_{2,\text{"S2"}} \,\middle|\, -\frac{d'}{2}\right)} = c_{2,\text{"S2"}}d'$$

$$c_{2,\text{"S2"}} = \frac{\ln\frac{\mathrm{HR}_1}{\mathrm{FAR}_1}}{d'} \tag{S60}$$

Enforcing the constraint that $c_{2,\text{"S2"}} \geq c_1$ yields a final solution for $c_{2,\text{"S2"}}$ as

$$c_{2,\text{"S2"}}^{\text{HF*}} = \max\left(\frac{\ln\frac{\text{HR}_1}{\text{FAR}_1}}{d'}, c_1\right) \tag{S61}$$

where the HF* superscript denotes that Equation S61 defines the optimal $c_{2,\text{"S2"}}$ for maximizing the difference between type 2 hit rate and type 2 false alarm rate (see **Table 1**, main manuscript).

4.2.2. Optimal Type 2 Criterion for Maximizing HR_2 – FAR_2 for "S1" Responses

For "S1" responses, the observer must set the type 2 criterion for "S1" responses, $c_{2,\text{"S1"}}$, so as to maximize $D_{2,\text{"S1"}}$:

$$D_{2,\text{"S1"}} = \text{HR}_{2,\text{"S1"}} - \text{FAR}_{2,\text{"S1"}} \tag{S62}$$

where $\text{HR}_{2,\text{"S1"}}$ and $\text{FAR}_{2,\text{"S1"}}$ are defined as in Eqs. S18 and S19, respectively. Thus, $D_{2,\text{"S2"}}$ can be written

$$D_{2,\text{"S1"}} = \frac{\Phi\left(c_{2,\text{"S1"}} \middle| -\frac{d'}{2}\right)}{(1 - \text{FAR}_1)} - \frac{\Phi\left(c_{2,\text{"S1"}} \middle| \frac{d'}{2}\right)}{(1 - \text{HR}_1)} \tag{S63}$$

Differentiating with respect $c_{2,\text{"S1"}}$ gives

$$\frac{\text{d}}{\text{d}c_{2,\text{"S1"}}}\left[D_{2,\text{"S1"}}\right] = \frac{1}{(1 - \text{FAR}_1)}\varphi\left(c_{2,\text{"S1"}} \middle| -\frac{d'}{2}\right) - \frac{1}{(1 - \text{HR}_1)}\varphi\left(c_{2,\text{"S1"}} \middle| \frac{d'}{2}\right) \tag{S64}$$

Setting the derivative equal to zero and solving for $c_{2,\text{"S1"}}$ gives

$$\frac{1}{(1 - \text{FAR}_1)}\varphi\left(c_{2,\text{"S1"}} \middle| -\frac{d'}{2}\right) = \frac{1}{(1 - \text{HR}_1)}\varphi\left(c_{2,\text{"S1"}} \middle| \frac{d'}{2}\right)$$

$$\ln\frac{(1-\mathrm{HR}_1)}{(1-\mathrm{FAR}_1)} = \ln\frac{\varphi\left(c_{2,\text{"S1"}}\,\middle|\,\frac{d'}{2}\right)}{\varphi\left(c_{2,\text{"S1"}}\,\middle|\,-\frac{d'}{2}\right)} = c_{2,\text{"S1"}}d'$$

$$c_{2,\text{"S1"}} = \frac{\ln\frac{1-\mathrm{HR}_1}{1-\mathrm{FAR}_1}}{d'} \tag{S65}$$

Enforcing the constraint that $c_{2,\text{"S1"}} \le c_1$ yields a final solution for $c_{2,\text{"S1"}}$ as

$$c_{2,\text{"S1"}}^{\mathrm{HF}^*} = \min\left(\frac{\ln\frac{1-\mathrm{HR}_1}{1-\mathrm{FAR}_1}}{d'}, c_1\right) \tag{S66}$$

where the HF* superscript denotes that Equation S66 defines the optimal $c_{2,\text{"S1"}}$ for maximizing the difference between type 2 hit rate and type 2 false alarm rate (see **Table 1**, main manuscript).

5. Expressing Criterion as Likelihood Ratio $\boldsymbol{\beta}$

An alternative way to express any decision criterion *c* is as the value of the likelihood ratio β of the probability density functions of decision axis values *x* for each stimulus, i.e. $\frac{f(x|\mathrm{S2})}{f(x|\mathrm{S1})}$, occurring at the location of the criterion (i.e. the likelihood ratio at *x* = *c*). In the equal variance signal detection theory model considered above, $f(x|\mathrm{S1}) = \varphi\left(x\,\middle|-\frac{d'}{2}\right)$ and $f(x|\mathrm{S2}) = \varphi\left(x\,\middle|\,\frac{d'}{2}\right)$, and so it follows that the likelihood ratio β occurring at *c* is given by

$$\beta = \frac{f(c|\mathrm{S2})}{f(c|\mathrm{S1})} = \frac{\varphi\left(c\,\middle|\,\frac{d'}{2}\right)}{\varphi\left(c\,\middle|-\frac{d'}{2}\right)} = e^{cd'} \tag{S67}$$

Eq. S67 can be used to re-express any of the type 1 or type 2 decision criteria derived above in terms of likelihood ratio. For instance, as discussed in the main text, the type 1 criterion that maximizes type 1 accuracy (from Eq. S6 above)

$$c_1^{A^*} = \frac{\ln \frac{p(S1)}{p(S2)}}{d'}$$

can be expressed in terms of the β value occurring at $c_1^{A^*}$ by plugging $c_1^{A^*}$ into Eq. S67:

$$\beta_1^{A^*} = \frac{f(c_1^{A^*}|S2)}{f(c_1^{A^*}|S1)} = e^{c_1^{A^*}d'} = \frac{p(S1)}{p(S2)}$$

Similarly, consider the type 2 criterion that maximizes type 2 reward for "S2" responses (from Eq. S35 above, using simplified notation where $Q_2 = \frac{(R_{CR2} - R_{FA2})}{(R_{hit2} - R_{miss2})}$ as in the main text):

$$c_{2,"S2"}^{R^*} = \max\left(\frac{\ln \frac{p(S1)}{p(S2)} + \ln Q_2}{d'}, c_1\right)$$

Re-expressing all elements of the left-hand side and right-hand side of the equation in terms of β and simplifying gives

$$\beta_{2,"S2"}^{R^*} = \max\left(\frac{p(S1)}{p(S2)} Q_2, \beta_1\right)$$

where $\beta_{2,"S2"}^{R^*}$ is the likelihood ratio at which the type 2 criterion for "S2" responses maximizes type 2 reward, and β_1 is the likelihood ratio corresponding to the (possibly suboptimal) type 1 criterion actually used by the observer.

In a similar way, all other type 1 and type 2 decision criteria derived above can be re-expressed in terms of β.
