# Supplementary material for: Optimal metacognitive decision strategies in signal detection theory
Source: Psychon Bull Rev. 2024 Nov 18;32(3):1041–69. doi: 10.3758/s13423-024-02510-7 (PMC12092500; doi:10.3758/s13423-024-02510-7)
Supplement: Supplementary file 3 — Supplementary file3 (PDF 693 KB) [file 13423_2024_2510_MOESM3_ESM.pdf]

Optimal Metacognitive Decision Strategies in Signal Detection Theory

Brian Maniscalco*, Lucie Charles*, & Megan A. K. Peters

Supplementary Material S3

Optimal type 2 criterion setting under the type 2 noise and type 2 signal loss models

1. Simulation methods

We used computational simulations to assess optimal type 2 criterion setting for the type 2 noise and type 2 signal loss models under the optimization contexts of maximizing type 2 reward and maximizing HR_2 - FAR_2. For simplicity, in all simulations we fixed $d' = 2$, $c_1 = 0$, and $p(S2) = 0.5$, and in the reward optimization simulations, we set $Q_2 = 3$. These parameter settings provided a fixed reference against which we investigated the effects of systematically varying model parameters controlling metacognitive sensitivity (σ_2 in the type 2 noise model and k in the type 2 signal loss model).

For the type 2 noise model, we performed simulations where σ_2 ranges from 0 to 2 in steps of 0.1. In the absence of type 2 noise, when $\sigma_2 = 0$, the model reduces to the standard SDT model described in **Supplementary Material S1**, and as σ_2 grows larger, metacognitive sensitivity decreases. The simulated cases span a range from $M_{\text{ratio}} = 1$ to $M_{\text{ratio}} \approx 0.3$, which more than covers the range of values of M_{ratio} typically encountered in real data.

For the type 2 signal loss model, we performed simulations where σ_2 is set to a small constant value of 0.1 and k ranges from 0 to 1 in steps of 0.02. In the absence of type 2 signal loss, when $k = 0$ and $\sigma_2 = 0.1$, the model yields slightly suboptimal metacognitive sensitivity due to the non-zero type 2 noise ($M_{\text{ratio}} = 0.99$), and as k grows larger, metacognitive sensitivity decreases. The simulated cases span a range from $M_{\text{ratio}} \approx 1$ to $M_{\text{ratio}} \approx 0$, which more than covers the range of values of M_{ratio} typically encountered in real data.

All simulations followed standard Monte Carlo approaches using 10,000,000 trials at each level of type 2 noise or signal loss tested. On each simulated trial, the parameter values of the model were used to generate evidence samples x_1 (for type 1 decisions) and x_2 (for type 2 decisions) from the presentation of either an S1 or S2 stimulus; see Eqs. 24 and 25 in the main text for formulae relating x_1 and x_2 for each model. Type 1 decision was set to “S2” when $x_1 > c_1$, and “S1” otherwise.

To explore how type 2 criterion setting influenced outcome measures, we simulated type 2 responses for a range of type 2 criteria spanning values [-4, 0] for “S1” responses and [0, 4] for “S2” responses,

in increments of 0.01. For each simulated trial, we computed the confidence responses generated by each of these type 2 criterion values based on where x_2 fell in relation to them. For a given type 2 criterion, confidence was set to “high” when $x_2 < c_{2,\text{“S1”}}$ (for “S1” responses) or when $x_2 > c_{2,\text{“S2”}}$ (for “S2” responses), and “low” otherwise. To determine optimal type 2 criteria for a given set of parameter values, we found the values of $c_{2,\text{“S1”}}$ and $c_{2,\text{“S2”}}$ that yielded the maximum type 2 outcome measure (e.g. maximum type 2 reward) across all simulated trials. Since parameter values were chosen so as to make outcomes for “S1” and “S2” responses symmetrical, for simplicity we present only data from simulated trials where the response was “S2” in the discussion below. Simulations were performed in Matlab (version 2017a).

We also used the simulated data to examine how model parameters controlling metacognitive sensitivity (σ_2 and k in the type 2 noise and type 2 signal loss models, respectively) were related to the SDT-based measure of metacognitive sensitivity, meta-*d′* (Maniscalco & Lau, 2012, 2014). To compute meta-*d′* for a given set of model parameters, we defined 9 type 2 criteria on either side of the type 1 criterion with values evenly spaced over the range $c_1 \pm [.1, 4]$. On each simulated trial, the x_2 evidence sample was compared to these criteria to derive a confidence rating on the scale of 1 - 10. We chose these settings because using a large rating scale with evenly spaced type 2 criteria allows for high-fidelity approximation of the type 2 ROC curves used for fitting meta-*d′*. Using these simulated confidence ratings in conjunction with other simulated data, we computed meta-*d′* using MLE fitting to response-specific type 2 ROC curves (Maniscalco & Lau, 2014). In presenting these simulation results (**Figure 9** in the main text), we summarize metacognitive efficiency as M_{ratio} = meta-*d′* / *d′*.

2. Type 2 noise model simulations

In **Figure S3.1A** we plot expected type 2 reward as a function of c_2 position for “S2” responses when σ_2 = 0.2 (top), 0.8 (middle), or 1.2 (bottom), using a similar format to **Figure 8C** in the main text. (Reminder: Since parameter values were chosen so as to make outcomes for “S1” and “S2” responses symmetrical, here we only plot “S2” responses for simplicity.) As in **Figure 8C,** it can be seen that as σ_2 increases, the reward function flattens out and its peaks move inwards towards the type 1 criterion, with the consequence that the optimal c_2 becomes more liberal.

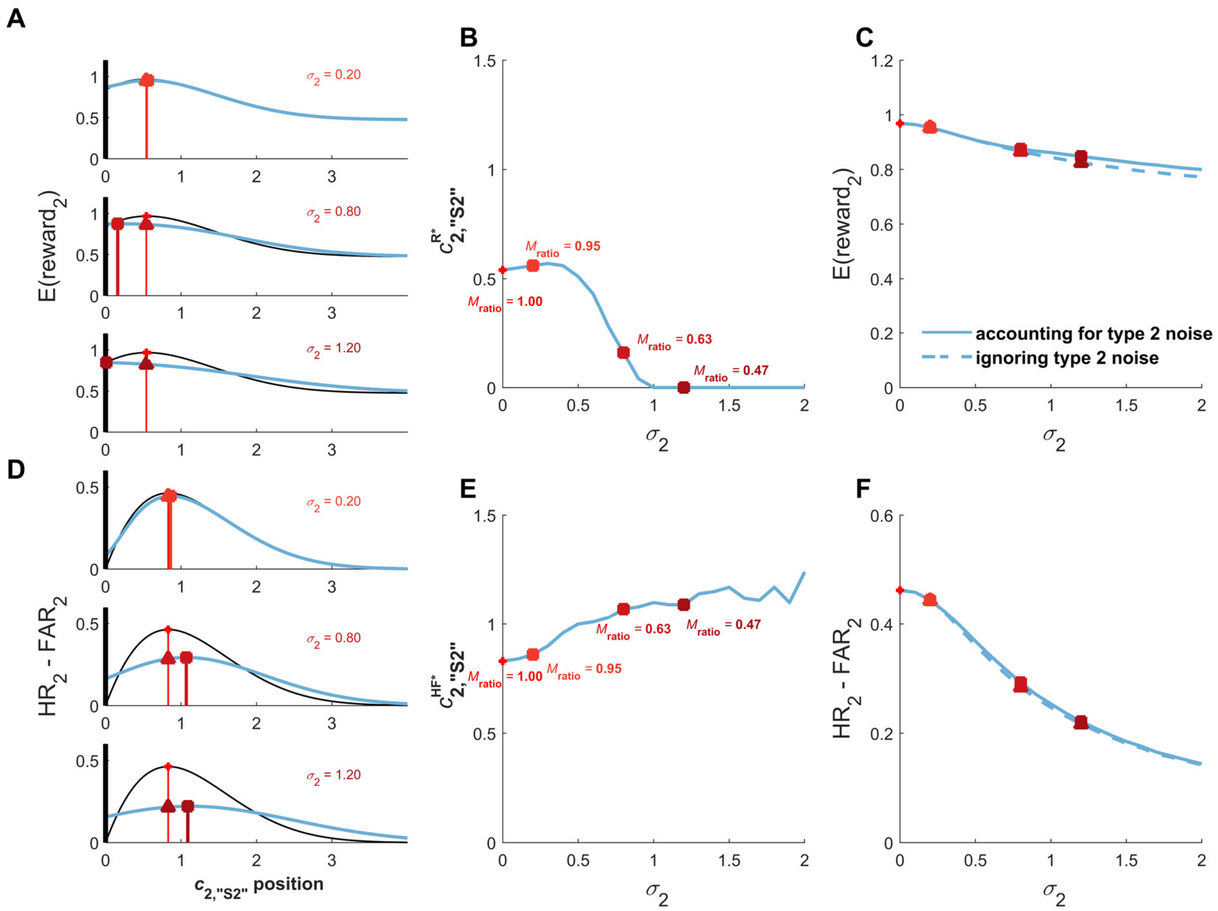

Figure S3.1. Simulation of the effect of type 2 noise on the optimal type 2 criterion for "S2" responses when optimizing type 2 reward **(A-C)** or HR_2 - FAR_2 **(D-F)**.

(A&D) Effect of different values of type 2 noise (from top to bottom, σ_2 = 0.2, 0.8 , or 1.2) on the expected reward (**A**, thick blue line) and on the type 2 hit and false alarm rate difference (**D**, thick blue line) according to the position of $c_{2,\text{"S2"}}$. The maxima of these outcome functions in the presence of type 2 noise are marked by red squares, and so the thicker red lines denoting the x-axis position of the red squares correspond to the optimal type 2 criteria $c^*_{2,\text{"S2"}}$ under type 2 noise. For comparison, the outcome functions in the *absence* of type 2 noise are displayed as thin black lines, with corresponding maxima and optimal type 2 criteria indicated by small red dots and connected thin red lines, respectively. Red triangles mark the value of the outcome functions *given* type 2 noise (blue lines) at the type 2 criteria values that are optimal under *no* type 2 noise (red lines indicating the maxima of the black outcome functions); thus, the red triangles indicate the outcome achieved if type 2 noise is present but not accounted for in type 2 criterion setting that is otherwise optimal.

(B&E) Value of the optimal type 2 criterion $c^*_{2,\text{"S2"}}$ as a function of type 2 noise σ_2 when optimizing type 2 reward **(B)** or HR_2 - FAR_2 **(E)**. Optimal type 2 criteria for the values of type 2 noise simulated in **(A)** and **(D)** are highlighted by red squares, and the corresponding values of M_{ratio} are indicated.

(C&F) Expected reward **(C)** and HR_2 - FAR_2 **(F)** achieved at the optimal type 2 criterion, plotted as a function of type 2 noise, separately for when type 2 noise is taken into account (solid blue lines) or ignored (dashed blue lines) in the estimation of the optimal type 2 criterion. When type 2 noise is taken into account (solid blue lines), the optimal type 2 criterion is set to the value that maximizes the

outcome function under type 2 noise, yielding the optimal outcome (e.g. as illustrated for three noise levels in panels **(A)** and **(D)** by red squares). Red squares in **(C)** and **(F)** (partially occluded by red triangles in these plots) indicate the same values as red squares in panels **(A)** and **(D)**. When type 2 noise is present but ignored (dashed blue lines), the type 2 criterion is set to the value that maximizes the outcome function when erroneously assuming type 2 noise is zero, yielding a suboptimal outcome (e.g. as illustrated for three noise levels in panels **(A)** and **(D)** by red triangles). Red triangles in **(C)** and **(F)** (partially occluded by red squares in these plots) indicate the same values as red triangles in panels **(A)** and **(D)**. Notably, there is minimal benefit of adjusting $c_{2,\text{"S2"}}$ to account for type 2 noise: the solid and dashed lines in **(C)** and **(F)** are almost entirely overlapping.

The relationship between σ_2 and optimal $c_{2,\text{"S2"}}^{\mathrm{R}^*}$ in the reward optimization context is characterized more fully in **Figure S3.1B**, which shows the optimal $c_{2,\text{"S2"}}^{\mathrm{R}^*}$ becoming increasingly liberal until it hits the most liberal possible value at $c_{2,\text{"S2"}}^{\mathrm{R}^*} = c_1$, which occurs when $\sigma_2 \approx 1$ at an M_{ratio} value of ≈ 0.5. We note, however, that increasing type 2 noise does not always make $c_{2,\text{"S2"}}^{\mathrm{R}^*}$ more liberal: If we set Q_2 = 6 instead of Q_2 = 3, increasing type 2 noise actually makes the optimal c_2 more conservative, not more liberal (data not shown). We encourage the interested reader to use the opt_t2c toolbox (https://github.com/CNClaboratory/opt_t2c) to explore the consequences of different reward scenarios in more detail.

In addition to knowing how type 2 noise affects the location of the optimal type 2 criterion, it is also very relevant to know how much these changes actually affect the magnitude of expected reward. Put simply, how much will the observer lose if they ignore the presence of type 2 noise, and instead use the optimal c_2 value for the σ_2 = 0 case? We explore this question in **Figure S3.1C**. For the same simulation parameters used above, we see that the magnitude of lost expected reward due to neglecting type 2 noise is quite small – especially when σ_2 < 1, which corresponds to M_{ratio} > 0.5, as is commonly observed in empirical studies. Thus, for these settings of type 1 performance and type 2 reward contingencies (as summarized in the measure Q_2), an observer with typical metacognitive efficiency (M_{ratio} > 0.5) would not suffer much loss of expected type 2 reward by simply neglecting the effect of type 2 noise on type 2 criterion setting.

Finally, we see qualitatively similar results when using the difference between type 2 hit and false alarm rate (HR_2 - FAR_2) as the measure to be optimized. First, as above, in **Figure S3.1D** we plot the expected difference between type 2 hit and false alarm rates as a function of c_2 when σ_2 = 0.2 (**Figure S3.1D,** top), 0.8 (**Figure S3.1D,** middle), or 1.2 (**Figure S3.1D,** bottom). We can see that type 2 noise has a modest effect on placement of the optimal type 2 criterion (**Figure S3.1E**), but in practice the observer can simply neglect the effect of type 2 noise without experiencing an appreciable loss in type 2 performance (**Figure S3.1F**). As previously illustrated in **Figure 8** of the main text, comparing panels **A**, **B**, **D**, and **E** of **Figure S3.1** shows that type 2 noise can make the optimal type 2 criterion more liberal or conservative depending on the measure to be optimized and the parameter settings of the model.

3. Type 2 signal loss model simulations

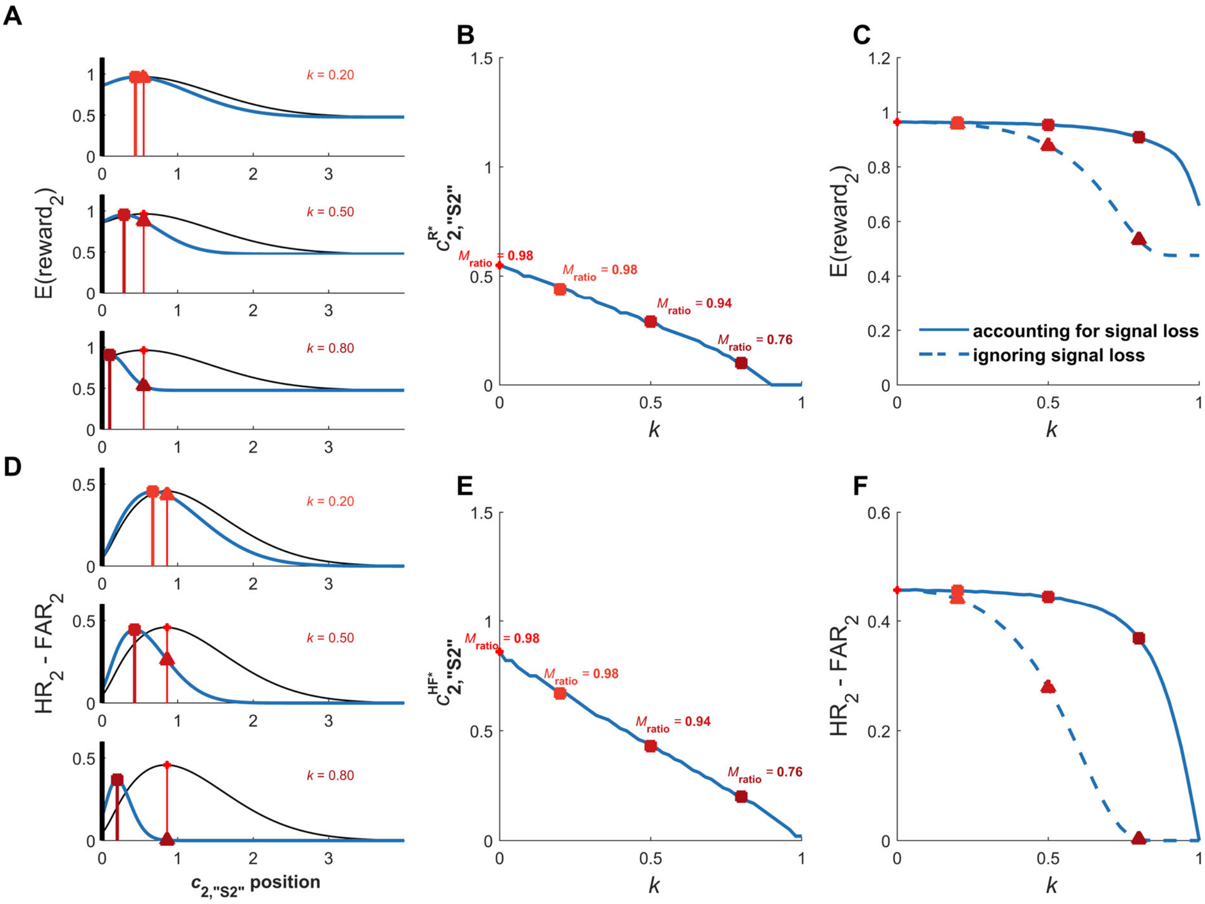

Figure S3.2. Simulation of the effect of type 2 signal loss (k) on the optimal type 2 criterion for "S2" responses when optimizing type 2 reward **(A-C)** or HR_2 - FAR_2 **(D-F)**.

(A&D) Effect of different values of type 2 signal loss k (from top to bottom, k = 0.2, 0.5, or 0.8) on the expected reward (**A**, thick blue line) and on HR_2 - FAR_2 (**B**, thick blue line) according to the position of $c_{2,\text{"S2"}}$, given a small amount of type 2 noise (σ_2 = 0.1). The maxima of these outcome functions in the presence of type 2 signal loss are marked by red squares, and so the thicker red lines denoting the x-axis position of the red squares correspond to the optimal type 2 criteria $c^*_{2,\text{"S2"}}$ under type 2 signal loss. For comparison, the outcome functions in the *absence* of type 2 signal loss but *with* a small amount of type 2 noise (k = 0 and σ_2 = 0.1) are displayed as thin black lines, with corresponding maxima and optimal type 2 criteria indicated by small red dots and connected thin red lines, respectively. Red triangles mark the value of the outcome functions *given* type 2 signal loss (blue lines) at the type 2 criteria values that are optimal under *no* type 2 signal loss (red lines indicating the maxima of the black outcome functions); thus, the red triangles indicate the outcome achieved if type 2 signal loss is present but not accounted for in type 2 criterion setting that is otherwise optimal.

(B&E) Value of the optimal type 2 criterion $c^*_{2,\text{"S2"}}$ as a function of type 2 signal loss k when optimizing type 2 reward **(B)** or HR_2 - FAR_2 **(E)**. Optimal type 2 criteria for the values of type 2 signal loss simulated in **(A)** and **(D)** are highlighted by red squares, and the corresponding values of M_{ratio} are indicated.

(C&F) Expected reward **(C)** and HR_2 - FAR_2 **(F)** achieved at the optimal type 2 criterion, plotted as a function of type 2 signal loss, separately for when type 2 signal loss is taken into account (solid blue line) or ignored (dashed blue line) in the estimation of the optimal type 2 criterion. When type 2 signal loss is taken into account (solid blue lines), the optimal type 2 criterion is set to the value that maximizes the outcome function under type 2 signal loss, yielding the optimal outcome (e.g. as illustrated for three signal loss levels in panels **(A)** and **(D)** by red squares). Red squares in **(C)** and **(F)** indicate the same values as red squares in panels **(A)** and **(D)**. When type 2 signal loss is present but ignored (dashed blue lines), the type 2 criterion is set to the value that maximizes the outcome function when erroneously assuming type 2 signal loss (and type 2 noise) is zero, yielding a suboptimal outcome (e.g. as illustrated for three signal loss levels in panels **(A)** and **(D)** by red triangles). Red triangles in **(C)** and **(F)** indicate the same values as red triangles in panels **(A)** and **(D)**. Unlike under the type 2 noise model, there is an appreciable benefit from adjusting $c_{2,\text{“S2”}}$ to account for signal loss, especially at higher levels of signal loss: the solid and dashed lines in **(C)** and **(F)** diverge as *k* increases.

In **Figure S3.2** we conduct a similar analysis as that shown in **Figure S3.1**, but now applied to the type 2 signal loss model. In **Figure S3.2A** we plot expected type 2 reward as a function of c_2 position for “S2” responses when k = 0.2 (top), 0.5 (middle), or 0.8 (bottom). In **Figure S3.2B** we then plot the effect of *k* on optimal type 2 criterion setting. Similar to the results for the type 2 noise simulation (**Figure S3.1B**), we see that as *k* increases and M_{ratio} decreases, the optimal c_2 becomes increasingly liberal. We also see in **Figure S3.2C** that in this model, neglecting type 2 signal degradation has more severe consequences for loss in expected reward than neglecting type 2 noise did in **Figure S3.1C**, even though the simulations span similar ranges of M_{ratio} and use identical values for *d'*, c_1, *p*(S2), and Q_2.

Lastly, we can examine the results of type 2 signal loss on the optimal c_2 for maximizing the difference between type 2 hits and false alarms, HR_2 - FAR_2. As above, in **Figure S3.2D** we plot HR_2 - FAR_2 as a function of c_2 when k = 0.2 (top), 0.5 (middle), or 0.8 (bottom). As *k* increases and M_{ratio} decreases, the optimal c_2 becomes increasingly liberal (**Figure S3.2E**), in contrast to the type 2 noise model where the optimal c_2 becomes increasingly conservative as σ_2 increases and M_{ratio} decreases in this optimization context (**Figure S3.1E**). Similar to the results for maximizing reward (**Figure S3.2C**), the penalty in HR_2 - FAR_2 incurred by neglecting to take type 2 signal loss into account in computing the optimal c_2 is quite salient (**Figure S3.2F**).

References

Maniscalco, B., & Lau, H. (2012). A signal detection theoretic approach for estimating metacognitive sensitivity from confidence ratings. *Consciousness and Cognition*, *21*(1), 422–430.

Maniscalco, B., & Lau, H. (2014). *Signal detection theory analysis of type 1 and type 2 data: meta-d', response-specific meta-d', and the unequal variance SDT mode* (S. M. Fleming & C. D. Frith

(eds.); pp. 25–66). Springer.
